# The value of universally available raw NMR data for transparency, reproducibility, and integrity in natural product research†
†Electronic supplementary information (ESI) available: Original NMR data (FIDs) of many cases discussed in this review are made available at DOI: http://dx.doi.org/10.7910/DVN/WB0DHJ. See DOI: 10.1039/c7np00064b


**DOI:** 10.1039/c7np00064b

**Published:** 2018-07-13

**Authors:** James B. McAlpine, Shao-Nong Chen, Andrei Kutateladze, John B. MacMillan, Giovanni Appendino, Andersson Barison, Mehdi A. Beniddir, Maique W. Biavatti, Stefan Bluml, Asmaa Boufridi, Mark S. Butler, Robert J. Capon, Young H. Choi, David Coppage, Phillip Crews, Michael T. Crimmins, Marie Csete, Pradeep Dewapriya, Joseph M. Egan, Mary J. Garson, Grégory Genta-Jouve, William H. Gerwick, Harald Gross, Mary Kay Harper, Precilia Hermanto, James M. Hook, Luke Hunter, Damien Jeannerat, Nai-Yun Ji, Tyler A. Johnson, David G. I. Kingston, Hiroyuki Koshino, Hsiau-Wei Lee, Guy Lewin, Jie Li, Roger G. Linington, Miaomiao Liu, Kerry L. McPhail, Tadeusz F. Molinski, Bradley S. Moore, Joo-Won Nam, Ram P. Neupane, Matthias Niemitz, Jean-Marc Nuzillard, Nicholas H. Oberlies, Fernanda M. M. Ocampos, Guohui Pan, Ronald J. Quinn, D. Sai Reddy, Jean-Hugues Renault, José Rivera-Chávez, Wolfgang Robien, Carla M. Saunders, Thomas J. Schmidt, Christoph Seger, Ben Shen, Christoph Steinbeck, Hermann Stuppner, Sonja Sturm, Orazio Taglialatela-Scafati, Dean J. Tantillo, Robert Verpoorte, Bin-Gui Wang, Craig M. Williams, Philip G. Williams, Julien Wist, Jian-Min Yue, Chen Zhang, Zhengren Xu, Charlotte Simmler, David C. Lankin, Jonathan Bisson, Guido F. Pauli

**Affiliations:** a Center for Natural Product Technologies (CENAPT) , Program for Collaborative Research in the Pharmaceutical Sciences (PCRPS) , Department of Medicinal Chemistry and Pharmacognosy , College of Pharmacy , University of Illinois at Chicago , 833 S. Wood St. , Chicago , IL 60612 , USA . Email: gfp@uic.edu, mcalpine@uic.edu; b Department of Chemistry and Biochemistry , University of Denver , Denver , CO 80210 , USA; c Department of Chemistry and Biochemistry , University of California , Santa Cruz , CA 95064 , USA; d Dipartimento di Scienze Chimiche , Alimentari, Farmaceutiche e Farmacologiche , Universita` del Piemonte Orientale , Via Bovio 6 , 28100 Novara , Italy; e NMR Center , Federal University of Paraná , Curitiba , Brazil; f Équipe “Pharmacognosie-Chimie des Substances Naturelles” BioCIS , Univ. Paris-Sud , CNRS , Université Paris-Saclay , 5 rue J.-B. Clément, 92290 Châtenay-Malabry , France; g Department of Pharmaceutical Sciences , Federal University of Santa Catarina , Florianópolis , Brazil; h University of Southern California , Keck School of Medicine , Los Angeles , CA 90089 , USA; i Griffith Institute for Drug Discovery , Griffith University , Brisbane , QLD 4111 , Australia; j Institute for Molecular Bioscience , The University of Queensland , St. Lucia , QLD 4072 , Australia; k Division of Pharmacognosy , Section Metabolomics , Institute of Biology , Leiden University , P.O. Box 9502 , 2300 RA Leiden , The Netherlands; l Kenan and Caudill Laboratories of Chemistry , University of North Carolina at Chapel Hill , Chapel Hill , NC 27599 , USA; m University of Southern California , Huntington Medical Research Institutes , 99 N. El Molino Ave. , Pasadena , CA 91101 , USA; n Department of Chemistry , Simon Fraser University , Burnaby , BC V5A 1S6 , Canada; o School of Chemistry and Molecular Sciences , University of Queensland , St. Lucia , QLD 4072 , Australia; p C-TAC , UMR 8638 CNRS , Faculté de Pharmacie de Paris , Paris-Descartes University , Sorbonne, Paris Cité, 4, Aveue de l’Observatoire , 75006 Paris , France; q Skaggs School of Pharmacy and Pharmaceutical Sciences , University of California , La Jolla , San Diego , CA 92093 , USA; r Center for Marine Biotechnology and Biomedicine , Scripps Institution of Oceanography , La Jolla , CA 92093 , USA; s Pharmaceutical Institute , Department of Pharmaceutical Biology , Eberhard Karls University of Tübingen , Auf der Morgenstelle 8, 72076 Tübingen , Germany; t Department of Medicinal Chemistry , University of Utah , Salt Lake City , UT 84112 , USA; u NMR Facility , Mark Wainwright Analytical Centre , University of New South Wales , Sydney , NSW 2052 , Australia; v University of Geneva , Department of Organic Chemistry , 30 quai E. Ansermet , CH 1211 Geneva 4 , Switzerland; w Yantai Institute of Coastal Zone Research , Chinese Academy of Sciences , Chunhui Road 17 , Yantai 264003 , People's Republic of China; x Department of Chemistry , M/C 0212 , Virginia Polytechnic Institute and State University , Blacksburg , VA 24061 , USA; y RIKEN Center for Sustainable Resource Science , Wako , Saitama , 351-0198 , Japan; z Department of Pharmaceutical Sciences , College of Pharmacy , Oregon State University , Corvallis , OR 97331 , USA; aa Department of Chemistry and Biochemistry and Skaggs School of Pharmacy and Pharmaceutical Sciences , University of California , San Diego , 9500 Gilman Drive MC-0358, La Jolla , CA 92093 , USA; ab College of Pharmacy , Yeungnam University , 280 Daehak-ro , Gyeongsan , Gyeongbuk 38541 , Republic of Korea; ac Department of Chemistry , University of Hawaii at Manoa , 2545 McCarthy Mall , Honolulu , HI 96822 , USA; ad NMR Solutions Limited , Puijonkatu 24B5 , 70110 , Kuopio , Finland; ae FRE CNRS 2715 , IFR 53 , Université de Reims Champagne-Ardenne , Bât. 18, Moulin de la Housse, BP 1039 , 51687 Reims , Cedex 2 , France; af Department of Chemistry and Biochemistry , University of North Carolina at Greensboro , Greensboro , NC 27402 , USA; ag Department of Chemistry , Department of Molecular Medicine , and Natural Products Library Initiative at the Scripps Research Institute , Jupiter , FL 33458 , USA; ah Instituto de Química , Universidad Nacional Autónoma de México , Ciudad de México 04510 , Mexico; ai University of Vienna , Department of Organic Chemistry , Währingerstrasse 38 , A-1090 Vienna , Austria; aj Department of Chemistry , University of California , Davis, One Shields Avenue , Davis , CA 95616 , USA; ak Institute of Pharmaceutical Biology and Phytochemistry (IPBP) , University of Münster , Pharma Campus , Corrensstrasse 48 , D-48149 Münster , Germany; al Institute of Pharmacy , Pharmacognosy , Member of CMBI , University of Innsbruck , Innrain 80-82 , 6020 Innsbruck , Austria; am Institute of Inorganic and Analytical Chemistry , Friedrich-Schiller-University , D-07743 Jena , Germany; an Dipartimento di Farmacia , Università; di Napoli Federico II , Via Montesano 49 , 80131 Napoli , Italy; ao Laboratory of Marine Biology and Biotechnology , Qingdao National Laboratory for Marine Science and Technology , Key Laboratory of Experimental Marine Biology , Institute of Oceanology , Chinese Academy of Sciences , Nanhai Road 7 , Qingdao 266071 , People's Republic of China; ap Departamento de Química , Universidad del Valle , AA 25360 , Cali , Colombia; aq State Key Laboratory of Drug Research , Shanghai Institute of Materia Medica , Chinese Academy of Sciences , 555 Zu Chong Zhi Road, Zhangjiang Hi-Tech Park , Shanghai 201203 , People's Republic of China; ar Department of Nanoengineering , University of California , La Jolla , San Diego , CA 92093 , USA

## Abstract

Covering: up to 2018

With contributions from the global natural product (NP) research community, and continuing the Raw Data Initiative, this review collects a comprehensive demonstration of the immense scientific value of disseminating raw nuclear magnetic resonance (NMR) data, independently of, and in parallel with, classical publishing outlets. A comprehensive compilation of historic to present-day cases as well as contemporary and future applications show that addressing the urgent need for a repository of publicly accessible raw NMR data has the potential to transform natural products (NPs) and associated fields of chemical and biomedical research. The call for advancing open sharing mechanisms for raw data is intended to enhance the transparency of experimental protocols, augment the reproducibility of reported outcomes, including biological studies, become a regular component of responsible research, and thereby enrich the integrity of NP research and related fields.

## Abstract 2

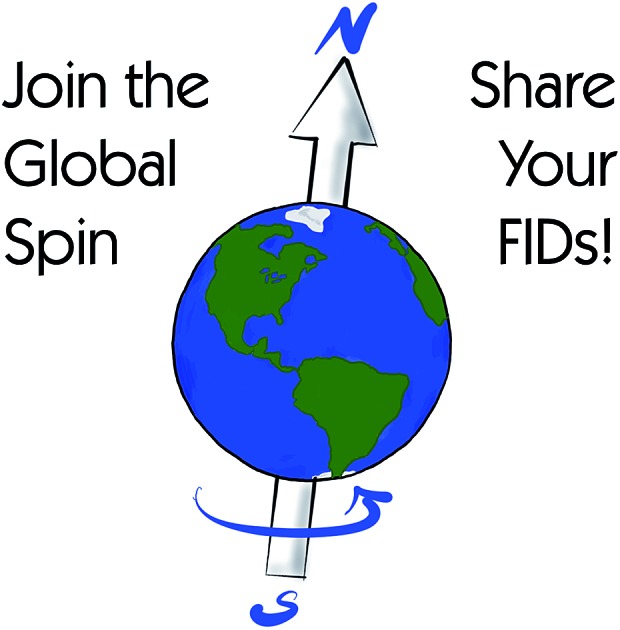
With contributions from the global natural product (NP) research community, and continuing the Raw Data Initiative, this review collects a comprehensive demonstration of the immense scientific value of disseminating raw nuclear magnetic resonance (NMR) data, independently of, and in parallel with, classical publishing outlets.

## 1 Introduction

### 1.1 Preamble

**Fig. 1:**
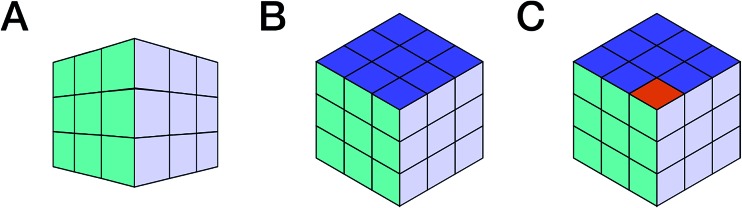
The rigor and integrity of structure elucidation and chemical identity depend not only on the type of data used to build the evidence, but importantly also on the point of view from which they are analyzed. This can be symbolized by looking at Rubik's cube from various viewpoints: perspective (A) may lead to the conclusion that the cube is solved. The two other projections, (B) and (C), are both compatible with (A) and isometric. Both increase the amount of visible information, but while B confirms the original hypothesis derived from (A), (C) refutes it. Following this analogy, the availability of raw (NMR) data enables researchers to view the entire “cube of evidence” from the same and/or from different angles. Thus, raw (NMR) data is an important means of enhancing transparency, reproducibility, and integrity, and even empowers investigators to use existing evidence to generate new scientific insights.

Throughout organic chemistry, and especially in natural products (NPs), where new bioactive metabolites are frequently isolated in minute, often sub-milligram quantities, nuclear magnetic resonance (NMR) has become the primary tool for structure determination. Typically, practitioners “extract” the structural information from NMR spectra that were generated *via* Fourier Transformation (FT) of free induction decays (FIDs), which represent the actual (raw) spectroscopic data from the excited nuclear spins in the NMR experiment (“spin choreography”). The deduction of structural information entails not only human interpretation and viewpoints (Fig. 1), but commonly also involves a significant loss of information (*e.g.*, signal phase, peak shape, and signal multiplicity in tabulated representations), which leads to the inability to reprocess the spectra *ab initio* and/or employ computational tools to derive additional information from the same experimental data. For example, extracting the complete information contained in the FID of the most basic and sensitive NMR experiment, 1D ^1^H NMR, can avoid the ubiquitous nondescript designation of “multiplet” and exemplifies the concept of exploiting raw NMR data for additional information (*e.g.*, Section 3 Structure Revision). The importance of extracting all of the information contained in an experimental data set is exemplified by the simple analogy presented in (Section 1.2 Dimensionality and Completeness).

This community-driven review calls for a re-examination of NMR-based structural analysis of NPs and represents the logical next step in the NMR Raw Data Initiative that commenced in 2016.^1^ The seven major rationales used to organize this text evolve from the urgent need for raw NMR data dissemination and are explained in Section 2 Introduction to the Organization of this Review. This led to the separation of the material into sections that cover chemical structure (Sections 3–5), analytical methodology (Sections 4–7), followed by applications and future perspectives (Sections 8–10) of raw NMR data. Located at the heart of the intent to promote the free dissemination of raw NMR data, Section 10 Conclusions & Outlook should be of particular interest to scientists increasing the use of NMR in NP research.

### 1.2 Dimensionality and completeness

Consider a picture of a Rubik's cube: the full 3D object cannot be captured by a single 2D picture, as it only provides a projection of the original object. The reduced dimensionality makes the representation incomplete, as observed in Fig. 1, and the incompleteness may lead to false conclusions. *E.g.*, projection A (Fig. 1) does not permit conclusions on solving the puzzle. No faithful conclusion is possible until at least five faces have been examined, which requires at least two projections since no more than three faces may be observed at once. A single projection may lead to an erroneous conclusion. Further projections increase the amount of available information, which may either confirm the original hypothesis or refute it (B *vs.* C, respectively, in Fig. 1).

Now consider a molecule. Each NMR experiment can be seen as a projection of the original spin system. The structural elucidation may require several projections/experiments to reconstruct the full picture, *i.e.*, approach the complete Hamiltonian as closely as possible. Note that, for the Rubik's cube, five of the total of six faces is sufficient for absolute certainty. In chemistry, however, structures are sometimes postulated on the basis of a single ^1^H NMR spectrum, often erroneously. Moreover, it is not possible to predict how many experiments will be required. Instead, the researcher will perform experiments based on budget, time, and the possibly the expectation that the analysis is complete once the first possible solution that matches all the available constraints (*e.g.*, chemical shifts, multiplicity, and correlations) has been found. Often, solutions are proposed based on previous results obtained for similar molecules; yet other solutions may exist and further experiments be required to single out the correct structure. Thus, an“elucidated” structure can be viewed as a possible solution that fits the available experimental data.

While other factors may contribute to erroneous structural assignments, the urge to stop after an apparent solution and failure to recognize that more than one structure can be equally or more consistent with the experimental data is likely the root cause of the errors. Computer-Aided Structure Elucidation (CASE) software^2^ is invaluable for overcoming this limitation by finding all structures which are consistent with the available data. Moreover, CASE tools are capable of ranking candidate structures by comparison of experimental and empirically predicted ^1^H and ^13^C chemical shifts, and remaining ambiguities can be resolved by inclusion of DFT calculations.^3^

Once an incorrect structure has been detected, the correct structure may still not be obvious, particularly if the structure is unusual.^4^ In such cases, CASE software can be valuable by providing probable structures for further consideration. While this can potentially be done using the tabulated correlation data, access to the raw NMR data it is valuable or even essential for this process. Collectively, the uncertainty inherent to structure elucidation is significant. Moreover, new structures are published daily without their corresponding experimental support, or with the compressed molecular formula strings (*e.g.*, Simplified Molecular Input Line Entry System [SMILES]), making peer-review a difficult or an almost impossible task. In this context it is safe to assume that the literature may contain erroneous structures and that a strategy is needed to deal with this issue.

### 1.3 Human and machine processing of NMR data

Progress in cheminformatics permitted the building of tools to help validate assignments and, thus, unveil incorrect structures.^5^–^8^ Indeed, computers may calculate all the solutions allowed by a potentially incomplete set of constraints. Software already exists that can handle all aspects of interpretation of NMR spectra, from peak-picking and chemical shift prediction^9^,^10^ to assignment and elucidation.^6^,^7^,^11^–^14^ The last two heavily rely on the accuracy of the chemical shift prediction, which in turn heavily relies on the quality and amount of known structure assignments available for training algorithms. As a consequence, most automatic spectral interpretation programs rely on large databases of previously assigned spectra; tools such as LSD [; http://www.univ-reims.fr/LSD] or CCASA^15^ developed by Nuzillard *et al.* are notable exceptions. Ensuring that these data are correctly assigned is essential to avoid continual propagation of structural errors. Therefore, even with the assistance of cheminformatics, the challenge of peer-reviewing published spectral interpretations still remains. But there may be another approach.

Acknowledging the fact that several signals can be assigned from integration and correlation constraints alone^11^,^12^ paves the way for unsupervised self-learning procedures that interpret spectra completely from scratch.^13^ During the first iteration, the procedure tries to assign as many atoms’-signal pairs as possible without the help of chemical shift constraints. In other words, assignment is performed based on signal area, multiplicity and correlations, and only unambiguous assignments are stored. These assignments link the observed chemical shifts to the assigned substructures, providing new knowledge to the chemical shift predictor. In a second iteration, the algorithm will reassign the same data, but this time using chemical shift constraints inferred from the knowledge just acquired. Iterations continue until a steady state is reached, *i.e.*, no new atom-NMR signal pairs can be assigned. When new data is submitted, the system assigns it and may run a new iteration. Hence, the algorithm builds its own database of assigned spectra without any human intervention.

Peak-picking should be implemented as part of this self-learning loop also. Indeed, modified data must be considered a representation of the original. A missing signal because of low signal to noise ratio or an additional signal from a poorly identified impurity are common errors that affect the outcomes of such a system. Although assignment is performed on peak-picked data, automatic peak-picking itself should be seen and implemented as an iterative process that ends when a successful assignment is found. Having brought assignment, prediction and peak-picking into a self-learning loop allowed the demonstration that a program may be conceived to avoid any human assumptions and faithfully generate all the solutions to the assignment problem. A similar approach can be implemented that applies CASE^2^ strategies and DFT calculations^3^ to generate all possible solutions to the elucidation problem and verify them. Such a program would see all possibilities allowed by the visible faces of the cube and allow thorough review of published assignments. That is, as long as the full, raw, unprocessed and unassigned data are published.

Hence, artificial intelligence may be applied to automatic structure elucidation. However, any operation performed on the truly raw, original NMR data (FID and associated information), as saved initially by the NMR spectrometer, can alter the final representation of the spectrum and may introduce errors. Consequently, any modification of the raw data should be considered part of the elucidation procedure and regarded as a process that can be improved. For this reason, only raw data must be input into the learning procedure of the automatic structure elucidator. Thus, developing new tools to assist researchers in their daily task requires large sets of high quality data stored in a correct manner. This goal can only be reached if the dissemination of original data becomes a standard component, if not a requirement, of established publication mechanisms.

### 1.4 Molecular transparency

Traceability and reliability of analytical results (detailed knowledge of total error and method specificity) as well as analytical data comparability are of utmost importance to make science transparent on a global level. This holds especially true if such results are key in decision making, as in medical diagnosis, food and feed safety, environmental pollution tracking, and many more areas. Even in the 21^st^ century, the scientific base of such undertakings is often not transparent, albeit that peer reviewed publications are daily business in applied and basic science. Lacking or incomplete information on the technologies used, or unclear declaration of utilized reference materials, hampers not only scientific progress, but also complicates the transfer from science to routine applications. Once an analytical strategy is applied in, and validated for, routine use, vagueness in the basic cornerstones of an assay, including (1) lack of information on identity and purity of reference materials, (2) a poorly documented chain of traceability in calibrator materials, and (3) missing clear-cut communicated measurement conditions, can all lead to unnecessary platform bias and an overall increase in inter-laboratory data scattering and inconsistency. As many scientists are involved with the establishment and execution of LC-/MS-driven assays for routine analysis, the importance of NMR in the total analytical process is unclear or unknown. However, NMR specialists are already aware of the power of “their” methodology.

Aside from X-ray crystallography, NMR spectroscopy is still the only spectroscopic method accepted for an unambiguous structure elucidation (not only for identification) of a molecular scaffold, especially in the realm of organic compounds. Today, high-resolution ^1^H and ^13^C NMR spectra become more widely recognized as being “molecular fingerprints”, which can even be predicted computationally. While two-dimensional ^1^H-detected experiments allow the transformation of ^1^H and ^13^C NMR resonances into molecular scaffolds, contemporary technologies still do not automate this process. Finally, while carbon–carbon connectivity mapping would complete NMR based molecular cartography, and despite recent progress with these experiments,^16^–^18^ this approach is limited by sensitivity and not used widely.

### 1.5 Molecular topography

By analogy, it is well known that modern terrestrial cartography has changed dramatically recently. Traditionally, the painstaking work started with planes doing analogue aerial photography and technicians deriving a (finally digital) terrain model thereof. This model still is a framework for detailed and accurate maps filled by information derived from the photographs or from terrestrial reconnaissance, often by foot. Such maps, used by almost everyone moving through the environment, have been replaced by highly automated processes relying on space technology based surveying by the “shuttle radar topography mission“ (SRTM) data gathered by the space shuttle Endeavour in 2000. Users who lack detailed knowledge of the involved technologies rely on the assumption that the “maps” involved are reliable. It is assumed that they are comparable and demand that the presented information is representing “the true” environment. However, in reality these claims are quite often not met. Traveling distances do vary, road conditions encountered are discrepant to mapped ones, and hiking maps are too often lacking detailed terrain visualization. Whenever “maps” are involved in legal processes, *e.g.*, when we use cadastral maps as planning tool, it is assumed that certain mapping products are accurate and precise two-dimensional presentations of the three-dimensional open space. It must not be overlooked, that these assumptions are made because the production of such maps is traceable to an agreed digital terrain model, the technological process of the 3D to 2D transformation is well described and its error margins are understood and communicated.

NMR spectroscopy is also a “mapping tool”, just on a molecular scale level. It is based on scientific inventions and breakthrough processes made 50+ years ago; its modern digital version, the FT NMR technology, has been on the market for more than four decades. Due to its technological complexity and costs, access to NMR spectroscopy has been limited to a very small number of practitioners. The latest “soft revolution” in the application of NMR spectroscopy reached the public about twenty years ago, meanwhile very successful first attempts have been made to transfer the NMR data interpretation from UNIX or Linux operated work station environments to desktop computers integrating NMR data into the everyday office. Now, for this type of software the Gardner hype cycle “trough of disillusionment” (which was very shallow) has been successfully transversed and a stable, productive working environment has been achieved.

Parallel to the development of NMR technologies, the interpretation of the NMR data is also experiencing constant change. Beginning from reporting selected NMR signals with molecular position annotations based on increment rules and similar estimation tools relying on conclusion by analogy, the introduction of high-resolution cryogenic magnets and the Nobel prize winning innovation of FT-NMR based 2D NMR spectra, changed the situation remarkably. Complete correlation of NMR signals and molecular positions became a must in describing a novel compound. Especially in NP science, comprehensive data representation was understood as mandatory whenever new NPs were claimed. In organic synthesis, standards were kept lower for significant periods of time, some prominent and well-ranked journals did not even request molecular position assignments of any of the NMR signals in spectral data. About a decade ago, Nicolaou and Synder^19^ showed in a comprehensive study that, in the process of NMR-based structure elucidation, erroneous structures resulted with noticeable frequency and ultimately reflected inadequate structure elucidation efforts.

Very recently, Wolfgang Robien affirmed this postulate by running the ^13^C NMR database CSEARCH against recently published structures. He again was able to show that erroneous assumptions in the structure elucidation process (*e.g.*, lacking spectral evidence, no 2D methods performed) were leading to incorrect structures.^20^

## 2 Introduction to the organization of this review

The numerous scientific rationales that support the urgency of public dissemination of raw NMR data fall into the following groups:

### 2.1 Rationale 1 – structure revisions

This represents the largest group and many cases can be grouped into sub-categories, the largest comprises structures originally proposed with an incorrect ring closure. Another, somewhat embarrassing subgroup, consists of structures which are blatantly incorrect or where, even with a cursory examination, of available data never should have been proposed. In these cases, the raw data would have allowed a reviewer to recommend changes and/or detect issues. A final set involves other types of revisions.

### 2.2 Rationale 2 – impurity detection and quantification

For several decades, the majority of NP research has been fueled by the search for bioactive compounds, drugs (human and veterinary), herbicides and other pesticides. This quest was focused on the use of bioactivity-guided fractionation. Here, a purity assessment of the final product assigned the bioactivity is critical, as high potency minor impurities invalidate the conclusions. Hence, both quantification and identity of impurities are critical.

### 2.3 Rationale 3 – dereplication

The bane of most NP chemists' endeavors is the “rediscovery” of a known compound. The schemes and protocols developed to avoid, or at least minimize, this occurrence have often been complex and varied. They have aimed at detecting known compounds as early in the discovery process as possible. However, none has ever had claims of sterling success. The fact that 1D ^1^H NMR and ^13^C spectra can serve as unique fingerprints of a given compound (for ^1^H methodology, see Sections 3.3 and 5.1) makes NMR a highly specific tool for dereplication, and whenever this can be applied early during fractionation (see Section 5.3), it provides a quantum leap in discovery.

### 2.4 Rationale 4 – enabling new methodology

Science advances with the development and use of new approaches and methods. This section features recently developed and utilized methods, which can provide the scientist with valuable tools to interpret spectra from raw data.

### 2.5 Rationale 5 – other nuclei

This section adds the perspective of ^19^F, ^15^N, and ^31^P NMR spectroscopy. Although fluorine occurs rarely in NPs, it is frequently introduced into derivatives to improve drug pharmacokinetics. Its high magnetic moment, broad chemical shift dispersion, and extensive coupling make ^19^F NMR spectroscopy almost a sub-specialty. Similar considerations apply to phosphorus, and the raw data from these spectra are every bit as data-intensive as those from a ^1^H NMR spectrum. Nitrogen is an important heteronucleus in many NPs, but ^15^N sensitivity has restrained a more widespread application to date. Raw data can play an important role to overcome this limitation by expanding the utility of valuable existing ^15^N NMR data with regard to structural interpretation.

### 2.6 Rationale 6 – data repositories

Raw NMR data only reaches its maximum potential if it is universally accessible. Unfortunately, chemists have fallen behind the geneticists in the establishment and general acceptance of a universal database. Although, several laudatory efforts have assembled databases, with some described here, the amount of NMR data generated around the world makes the compiling of a single database for each nucleus a growing, and already gargantuan, task, discussed further in the conclusions.

### 2.7 Rationale 7 – clinical applications

Most readers of this review will probably find this section alien to their everyday interests. However, those who have had need to take advantage of this foray of physics into the medical field will surely appreciate its capabilities and enjoy reading of how the raw data has its role here also, and the optimistic view anticipating quantum leaps forward in medicine from progress in this area.

## 3 Structure revision

Structural revision can occur at three points of scientific discovery, preferably prior to publication, either in the originating laboratory or at the manuscript review process, or less ideally post publication. One example which was only published after an initial misassignment was discovered in house is represented by the neolignan from *Magnolia grandiflora* L.^21^ This is an excellent example of the Rubik's cube philosophy discussed above. The structure, **1**, originally proposed on the basis of HRMS, ^1^H NMR and ^13^C NMR was questioned on the basis of biosynthetic considerations. A further examination of 2D NMR, specifically one-bond and long-range correlations from HMBC and HSQC experiments, respectively led to a revision to structure **2**,^21^ but this revision would not have been possible from the 1D data alone. In most cases of Structure Revision that see the light of day, the initial incorrect structure is not corrected in-house but published as such, and correction comes when another group isolates and/or studies the same compound. While one can only speculate about the likelihood of a published structure being incorrect, recent systematic studies employing relatively fast parametric/DFT hybrid computational methods have found substantial mismatches between predicted and published data.^22^–^24^ For a series of nearly 100 sesquiterpenes, discrepancies occurred for as many as 14% of the published structures and indicated the need for substantial structural revision.
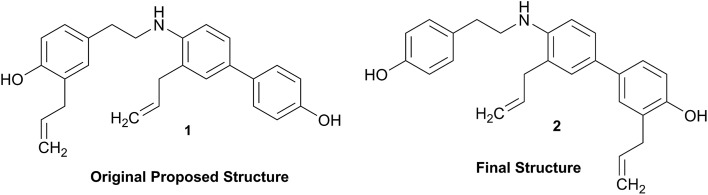


Moreover, concerns were expressed as early as in the mid/late 1970s by Zimmerman and co-workers (see footnotes 12 in ref. 25 4 and in ref. 26) regarding the exclusive use of spectroscopic structure elucidation methods while not including more classical approaches involving chemical synthesis and/or chemical degradation together with bulk analytical methods such as elemental analysis for a more thorough approach to structure elucidation. Similar concerns regarding the integration of chemical and spectroscopic structural analysis were expressed by Faulkner (page 1433 in ref. 27) and Robinson (in a letter to Chavrarti, as referred to in ref. 28). Following some (undocumented) statistical analyses, Zimmerman raised the potential apprehension that relying on spectroscopic evidence alone carried with it a substantial probability of structural misassignment. While a classical approach involving total synthesis may not be feasible within a reasonable time frame in NP research, it is of interest to compare Zimmerman's predicted probabilities of erroneous structures of 10–22% with the *ca.* 14% incidence rate found very recently by Kutateladze and co-workers.^22^–^24^ These findings confirm the validity of the cautionary notes raised 40+ years ago,^25^,^26^ and demonstrate the importance of purity and residual complexity^29^ in both analytical and NP chemistry: classical bulk analysis methods such as microanalytical and (mixed) melting point determinations are more sensitive to minor impurities than many of the contemporary spectroscopic methods. Notably, the demand for purity of bioactive NPs and other chemicals is essential for rigor and reproducibility of research outcomes.

Here, raw NMR data plays important roles in documentation by enabling the retrospective determination of the purity of previously investigated materials. Notably, the need for re-assignment of NMR spectra and/or achievement of a complete assignment of at least the full chemical shifts and coupling constants of the ^1^H and ^13^C framework, can be estimated to be much greater. Reflecting on the general gap in the assignment of the relatively complex ^1^H NMR signal patterns, this consideration affects the scientific context of structural correctness, the resulting reproducibility of downstream research, intellectual property issues, and their collective economic impact. The role of (raw) NMR data in the structural revision of NPs has been highlighted prominently in a recent review by Kubanek and co-workers.^30^

### 3.1 Incorrect ring closures: furan *vs.* pyrone ring systems

**Fig. 2:**
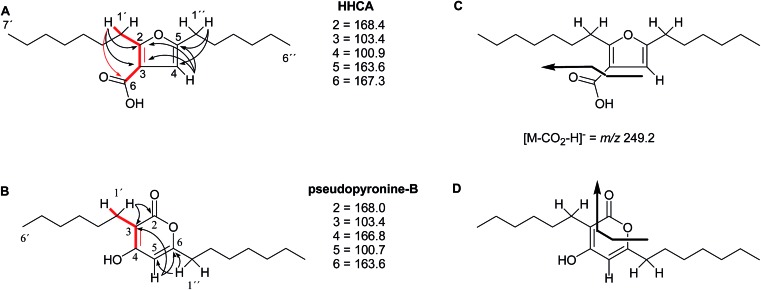
The putative (A) and revised (B) structure of 2-heptyl-5-hexylfuran-3-carboxylic acid (HHCA; **3**), which was reported as pseudopyronine-B. Arrows in A and B indicate ^1^H–^13^C HMBC correlations; red color indicates ^4^*J*_H,H_ coupling of interest. Panel C shows the putative explanation of the MS/MS fragmentation of HHCA in negative mode; fragmentation of the pseudomolecular ion [M – H]^–^ = *m*/*z* 293.2. Panel D provides the correct true explanation for the observed MS/MS fragment. The arrow with the solid line in (C) and (D) directly shows the decarboxylation process.

The putative new compound 2-heptyl-5-hexylfuran-3-carboxylic acid (HHCA; CAS 1256499-01-0, compound **3** in Fig. 2A) is produced by the rhizosphere bacterium *Pseudomonas* sp. strain SJT25.^31^ HHCA exhibits broad antifungal activity against several phytopathogens and was considered a new promising biopesticide. This led to further fermentation studies^32^ and a patent being filed and granted in 2012.^33^ However, biosynthetic considerations raised doubts about the structure. With 18 carbon atoms it was assumed that HHCA was generated by nine acetate units but these units could not be lined up, by a single, or a two chain-mechanism to give upon cyclization HHCA. A database search using the molecular sum formula pointed to pseudopyronine B, an α-pyrone-based compound with an identical NMR data set, that is produced also by several *Pseudomonas* species.^34^–^37^ Indeed, the UV-absorption (208 and 290 nm) spectrum and the ^13^C NMR data of pseudopyronine B (**4**) were nearly the same as those for other 3,6-disubstituted 4-hydroxy-2*H*-pyran-2-one-based compounds.^38^–^42^ Thus, the structure of HHCA has to be revised to that of **4** (Fig. 2A/B).
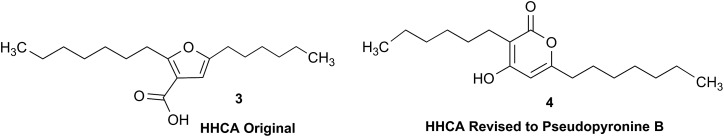


Unfortunately, the authors assigned the carbon atom C-6, resonating at 167.3 ppm in the ^13^C NMR spectrum together with a broad singlet signal at 10.31 ppm in the ^1^H NMR spectrum to a putative free carboxylic acid moiety, bound to a disubstituted furan ring. This conclusion was thought to be corroborated by IR absorption at 1635 cm^–1^ and a loss of *m*/*z* 44 (loss of the COOH group by decarboxylation, in the MS spectrum (Fig. 2C)). However, actually, the carbon atom C-6 of HHCA (*δ* 167.3 ppm) corresponds to C-4 of pseudopyronine B; and the OH group of the COOH of HHCA (*δ* 10.31 ppm) equals the OH group bonded to C-4 of pseudopyronine B. Furthermore, the observed broad IR absorptions at 1635 cm^–1^ represents an overlapping signal which is generated by the stretching frequencies of the tautomeric C=O bond^13^ and C_5_=C_6_ of the α-pyrone ring.^43^,^44^ In the MS spectrum, the loss a CO_2_ group is commonly observed from the pyrone ring system (Fig. 2D).^45^,^46^

**Fig. 3:**
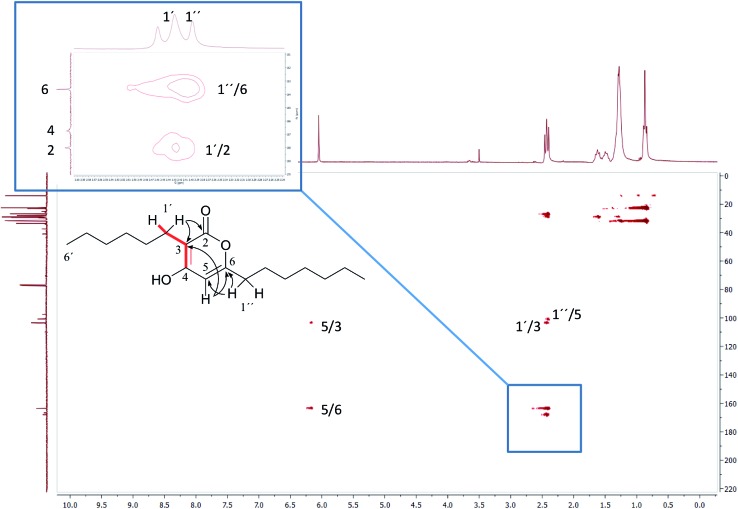
^1^H–^13^C HMBC NMR spectrum of pseudopyronine B (**4**); insert show details of the 160 ppm region.

In the original report of HHCA, the tri-substituted furan ring was deduced on the basis of ^13^C NMR shift values and HMBC correlations observed between H-4 and C-2, C-3, C-5 and C-1″, while the linkages of the alkyl chains were deduced from HMBC correlations from H_2_-1′ with C-2, C-3 and C-6 and from H_2_-1″ with C-4 and C-5. Regarding the ^1^H–^13^C HMBC correlations, the pair H_2_-1′–C-6 suggests a questionable ^4^*J*_C,H_ coupling, which indicated already that the original core was wrongly determined, because the HMBC experiment is in a standard setup optimized for 2–3 bonds. The observation of long-range coupling over four bonds is not impossible (*e.g.*, foremost in aromatic systems or as a W-coupling in planar aliphatic systems) but commonly presents a weak signal. In the case of a strong signal, it could be an indicator for a misassigned structure. The authors presented in the ESI† the HMBC map, however only a section from 0–120 ppm in the f1 dimension is shown, and the decisive range (150–170 ppm) is regrettably not visible. The availability of NMR raw data could have clarified this issue. During the course of the study of the biosynthetic origin of pseudopyronines, the Gross group re-isolated congener B (**4**) and observed no correlation between H_2_-1′ (*δ* 2.44 ppm) and C-6 (*δ* 167 ppm) from the ^1^H–^13^C HMBC NMR map (Fig. 3). It should be noted that a variety of more recent 2D NMR experiments improve the detection and/or distinction of ^2/3/4^*J*_C,H_ couplings, such as H2BC, LR-HSQMBC,^47^–^49^ and HSQMBC-COSY/TOCSY^50^ experiments (see also the review by Breton and Reynolds^51^).

Nevertheless, such a correlation can be much better rationalized by the pyrone than a furan ring structure. Finally, Gross and coworkers conducted labeling experiments employing doubly ^13^C-labeled acetate and confirmed in this way the structure by the determination and localization of intact acetate units *via* measurement of *J*_C,C_.^37^ Similarly, Reibarkh *et al.* have emphasized the utility of uniform ^13^C labeling of microbial NPs, which becomes feasible *via* the availability of uniformly ^13^C labeled glucose.^52^

### 3.2 Incorrect ring closures: the lipopeptide arthrofactin

In 1993, Imanaka and co-workers reported the isolation of the cyclic lipo-undecapeptide, arthrofactin from the bacterium *Arthrobacter* sp. MIS38. This compound possesses a high surface activity and was assigned the structure **5**.^53^ Later, the corresponding biosynthetic gene cluster was characterized.^54^ The gene cluster (*arfABC*) coded for the expected 11 NRPS modules, required for the assembly of the linear lipo-undecapeptide portion and a terminal tandem thioesterase (TE-I/TE-II). Particularly, the TE-I enzyme system is responsible for the hydrolysis and cyclization of the linear lipopeptide precursor. Nowadays, it is possible to predict the cyclization process by bioinformatics because the TE's reveal clades of enzymes that reflect the cyclization step. Bioinformatic analyses with the TE-I of ArfC led to the hypothesis that the ring closure occurred between Asp11 and Thr3 to give structure **6** instead of a lactone ring between Asp11 and the 3-hydroxy group of the fatty decanoic acid side chain as originally suggested.^55^
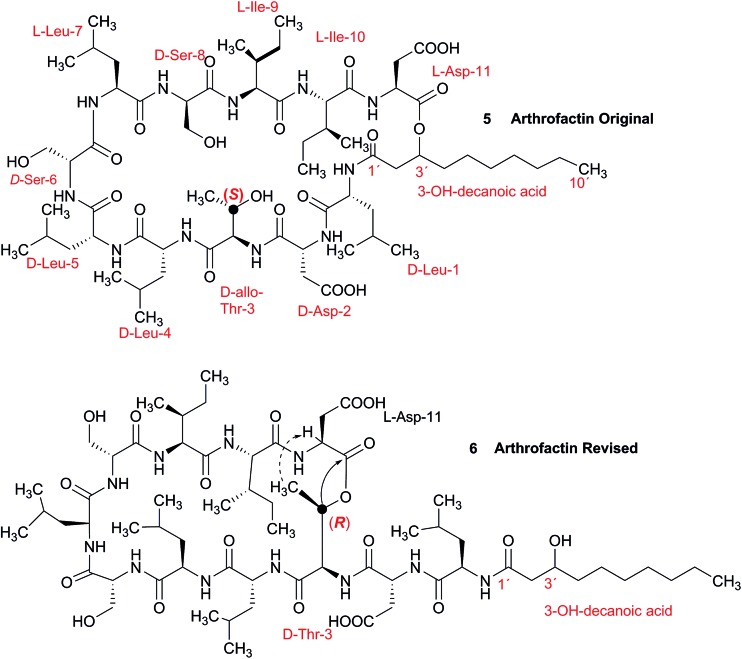


**Fig. 4:**
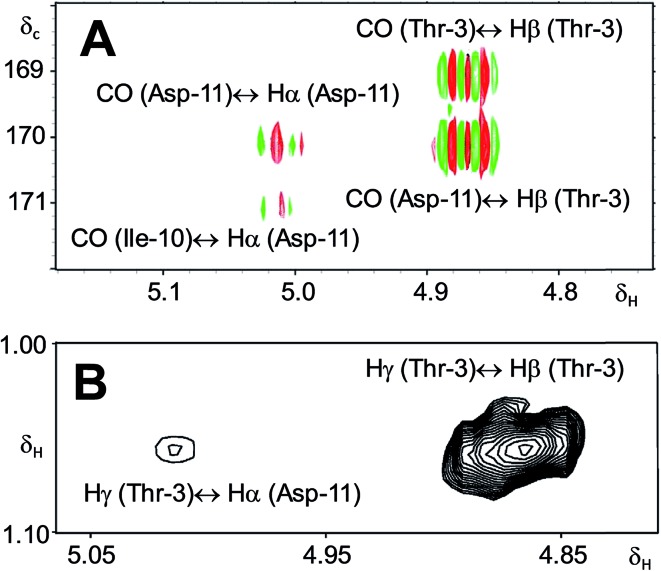
Selected regions of 2D NMR spectra of arthrofactin (**6**). (A) The ^1^H–^13^C HMBC 2D NMR spectrum indicated that both Hα of Asp11 and Hβ of Thr3 are coupled with the carbonyl of Asp11. (B) The ^1^H–^1^H NOESY spectrum exhibited key NOE correlations between Hγ of Thr3 and Hα of Asp11, indicative of the ring closure between Thr3 and Asp11.

A re-analysis of the ^1^H–^13^C HMBC correlation map and the ^1^H–^1^H NOESY correlations, enabled by the availability of the raw data, would have revealed problems with the first interpretation. The closure of the cyclic peptide between Thr3 and Asp11 was demonstrated using the following evidence: the carbonyl carbon of Asp11 shows a HMBC correlation with the Asp11 Hα and Thr3 Hβ hydrogens (Fig. 4A). Furthermore, the Thr3 Hγ shows a NOESY correlation with the Asp11 Hα (Fig. 4B). Therefore, the closure of the ring must be situated between the Asp11 carbonyl group and the Thr3 hydroxyl group.

### 3.3 Incorrect ring closures: the case of aquatolide

**Fig. 5:**
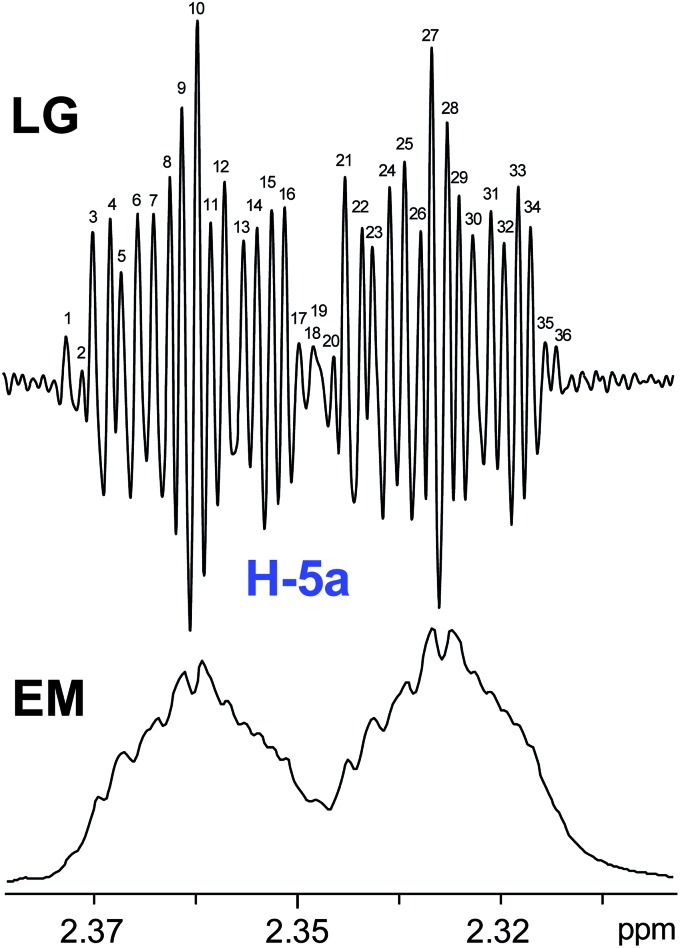
Comparison of the results of typical ^1^H NMR processing with spectrometer default settings (exponential multiplication [EM] with LB = 0.3 Hz; often the default processing scheme in NMR spectrometers) and lineshape-enhancing methods such as Gaussian–Lorentzian plus zero filling (LG) shows that raw data availability enables the analysis of what otherwise would be considered a multiplet or “br d” of H-5a in aquatolide (**8**). Representing a ddddq signal of near first order, a wealth of structural information can be extracted from raw data as simple as a 1D ^1^H NMR spectrum, for each of the hydrogen signals, yielding an almost complete structural picture of the aquatolide molecule from <200 kB of raw data.

The initial structure for the sesquiterpene aquatolide (**7**) described in *Asteriscus aquaticus*,^56^ contained an unusual bicyclo-hexane ring structure. This was revised recently to **8** by additional NMR experiments, X-ray diffraction analysis and quantum chemical computations,^4^ as well as by independent total synthesis.^57^,^58^ However, a thorough analysis of just the ^1^H NMR spectrum, enabled by the availability of the raw data, would have revealed problems with the first interpretation. The feasibility of this approach was demonstrated *via* HiFSA (^1^H iterative Full Spin Analysis) from the FIDs of the original 1D ^1^H NMR spectra,^59^ obtained with both the re-isolated natural^4^ and synthetic^57^ material. Using the PERCH software tool and an established HiFSA workflow,^60^–^62^ it was possible to extract no less than seven coupling constants from signals that had only been described as “multiplets” in the original work (see example of H-5a in Fig. 5). Some of these are surprising from either the original or the revised structure. *E.g.*, aquatolide shows a ^4^*J* coupling of 7.2 Hz through saturated carbons, but this is fully consistent with the quantum mechanical calculations from the revised structure. While being unexpectedly large and not leading to a “hidden” signal splitting, the 7.2 Hz coupling could be fully explained as being due to the spin–spin interaction between two bicyclic bridgehead hydrogens *via* two routes. It is important to note that the tabulated NMR data were/are not an adequate tool for the reader to verify the assignments, whereas the digital ^1^H NMR data provided this opportunity. NOESY and ^13^C NMR spectra were also important for differentiating between the initial and revised structures.
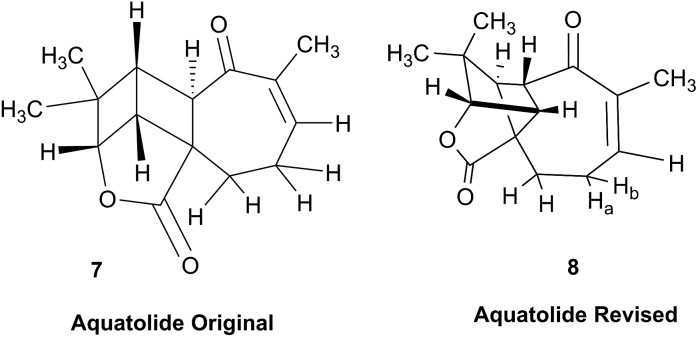


Evolving from the aquatolide study, was also the introduction of Quantum Interaction and Linkage Tables (QuILTs),^59^ which provide a checkerboard presentation rather than a classical table as a means of rapidly viewing the relationship between coupling constants and bonding proximity. The combination of available digital data and a more intuitive representation of the interpreted data, such as in QuILTs, would have pointed out the inconsistencies in the original structure that were in fact expressed in the *J*-coupling patterns and signal multiplicities. It should be noted that HiFSA profiles enable the calculation of NMR spectra at any desired resonance frequency, meaning that the NMR information extracted from a given spectrum becomes independent of the magnetic field strength. This is particularly useful for ^1^H NMR based dereplication, when reported data has used a different magnetic field. Compiling HiFSA data in the form of QuILTs has the added advantage of being a more intuitive representation for human interpretation and providing a tabular format that is closely related to the data matrices of spin simulation tools.

Although QuILTs provide a good check on the structure elucidation and a more comprehensive description of the ^1^H NMR spectra, they do have to be considered together with configurational arrangements. Chemical synthesis and X-ray crystallography will remain the final arbiter of structure determination. However, the former in particular will be greatly simplified by starting with the correct structure, and the initial structure is almost invariable the outcome of spectral analysis. The aquatolide case exemplifies the need for thorough and complete analysis of NMR spectra, and the need to go beyond first order visual analysis of a processed ^1^H NMR spectrum. It also reminds researchers of the illustrious quote the astronomer, Carl Sagan, whereby “extraordinary claims require extraordinary evidence”, which is widely considered a variation of the principle by the Bayesian statistician, Pierre-Simon Laplace, according to which “the weight of evidence for an extraordinary claim must be proportioned to its strangeness”.^63^ Finally, the case highlights the power of advanced post-acquisition processing in structure elucidation.

### 3.4 The case of coibamide A

**Fig. 6:**
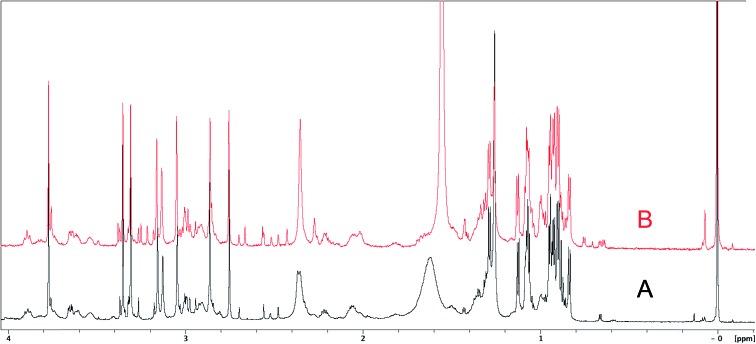
Partial ^1^H NMR spectra of the authentic natural product^64^ (**A**) and synthetic [d-Hiva^2^], [d-MeAla^11^]-coibamide^66^ (**B**).

**Fig. 7:**
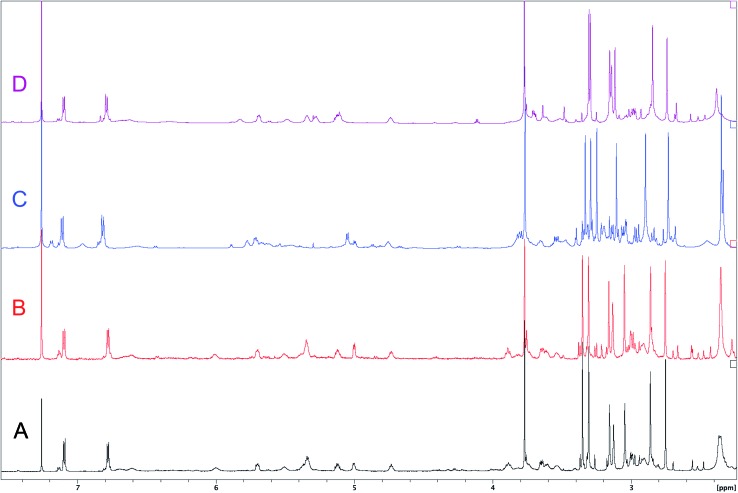
Downfield portion of the ^1^H NMR spectra of the authentic natural product (A),^64^ synthetic [d-Hiva^2^], [d-MeAla^11^]-coibamide (B),^153^ all-l-coibamide (C),^68^ and [d-MeAla^11^]-all-l-coibamide (D).^69^

The cyanobacterial coibamide A (**9**) is a highly *N*,*O*-methylated depsipeptide (1287 Da), comprising 11 residues with 13 stereogenic centers, that was originally proposed as the “all-L” diastereomer (**10**) in 2008.^64^ Ensuing attempts at total synthesis were initially plagued by inefficient coupling of the sterically hindered *N*-methyl amino acids, which promotes racemization and diketopiperazine formation,^65^ and requires tedious residue-specific optimization of coupling reagents and conditions. Ultimately, Yao *et al.*^66^ reported the configurational revision of coibamide A (**9**) in 2015, with inverted configuration of both the [Hiva] and [MeAla] residues compared to the originally assigned structure. The published ^1^H NMR spectra for this [d-Hiva^2^], [d-MeAla^11^]-coibamide A (**9**) and the NP were very similar (Fig. 6), while the ^13^C NMR spectra matched perfectly. The McPhail group collected and fully assigned comprehensive 2D NMR data for this synthetic product, confirming the match with the NP.^67^ However, the complexity of the ^1^H NMR spectrum for coibamide A, and their experience with ^1^H NMR analyses of synthesized methylated oligopeptides, highlighted the potential difficulty in discerning differences between the crowded ^1^H NMR spectra for closely related diastereomers of a NP with the size and number of stereocenters of coibamide A. Consideration of the potential for multiple *N*-methyl conformers (rotamers), and/or diastereomers arising from sluggish coupling reactions, as well as the presence of impurities, was critical in evaluating synthetic products and moving ahead with SAR studies. Before the configurational revision of coibamide A was reported, He *et al.*^68^ achieved the total synthesis in 2014 of the proposed “all-L” diastereomer **10**, which yielded ^1^H and ^13^C NMR data that clearly did not match those for the NP (Fig. 7), and was 1000-fold less cytotoxic. Notably, structure **10** also appeared to be more flexible than the NP (in CDCl_3_), as indicated by apparent *N*-methyl conformer signals, as judged by the chemical shift pattern and signal areas. Concurrently, while investigating the synthesis and SAR of coibamide A, Fujii and coworkers produced [d-MeAla]-epimer **11**,^69^ as well as several unpublished diastereomers. The latter diastereomers vary by single stereocenters and are under investigation for their variable biological activity, with potential uncoupling of cytotoxicity from their primary mechanism of action as inhibitors of cellular protein secretion^70^ involving the Sec61 translocon.
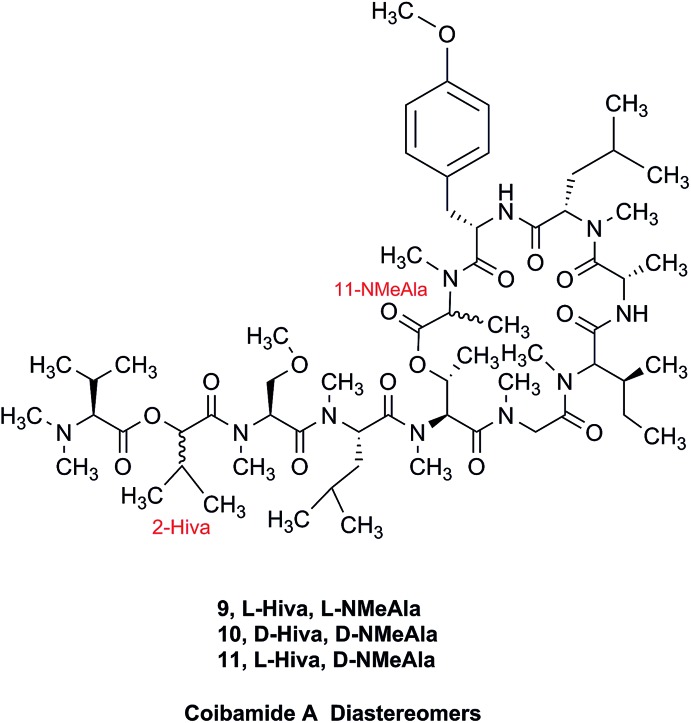


Accurate verification of the absolute structure of each synthetic product is, thus, critical. Thus far, the ^1^H NMR data for published diastereomers do show discernible differences and consistencies relevant to configuration (Fig. 7), especially when raw data is processed consistently and directly overlaid for comparison to detect slight chemical shift discrepancies and changes in signal shape of overlapped resonances. Access to raw NMR data for synthetic products has also allowed specific integration of minor and/or major signals for quantitative evaluation of the contribution of *N*-methyl conformers, diastereomers and impurities, which substantially affect the biological activity of coibamide compounds.

### 3.5 The structure of aldingenin B

The initially reported structure of aldingenin B (**12**), containing a highly unusual intramolecular ketal, was assigned based on extensive analysis of NMR spectral data (COSY, HMQC, HMBC).^71^ The reported structure was recently determined to be incorrect by total synthesis of **12** 72 An alternate five-membered hemiacetal structure (**13**), was proposed based on computational simulations of the ^1^H NMR spectrum of both the originally reported structure and the revised proposed structure with comparison to the experimental NMR data for the synthetic material corresponding to the reported structure and the original NMR spectrum of aldingenin B.^73^
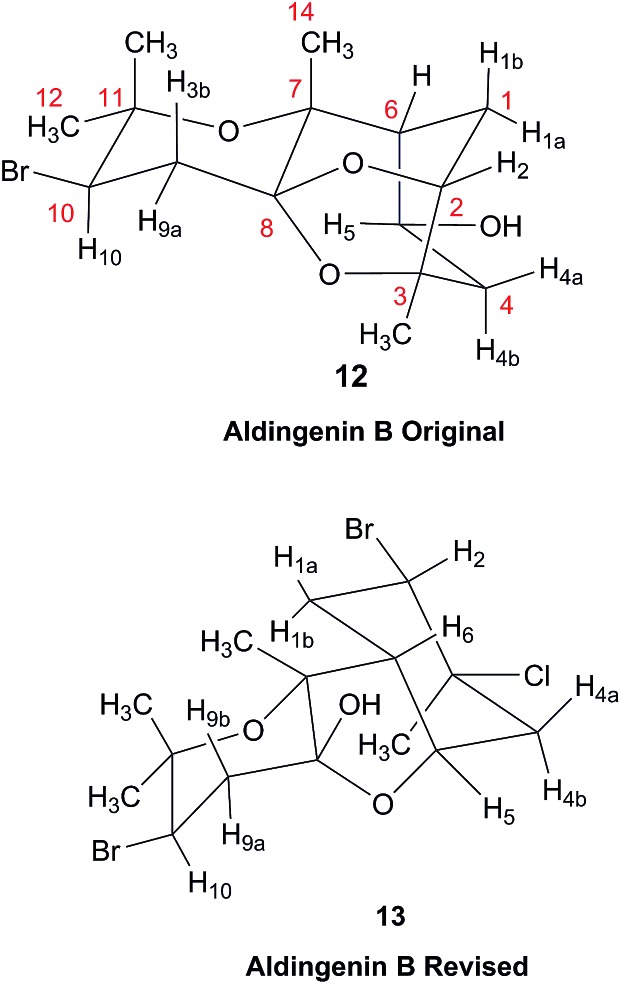


Inspection of models of the reported structure reveals the H-6–H-5 dihedral angle to be 90° (±2°); the expected coupling of such vicinally orthogonal hydrogens is <2 Hz. The natural sample displayed an 8.4 Hz coupling between these nuclei, while there was no detected coupling between H-5–H-6 in the synthetic sample. Furthermore, the reported coupling constants for the “bridgehead” hydrogens H-6 and H-2 in the natural sample were reported as 9.0, 8.4 and 9.6, 6.3 Hz respectively. The expected value of coupling constants of such bridgehead hydrogens is <4 Hz, as observed in the couplings of H-2 (*J* = 3.6, 1.8 Hz) and H-6 (br.s) in the synthetic sample and similar structures reported by Dudley.^74^ Additionally, the HMBC correlation map of the natural sample did not display an H-2–C-8 correlation, whereas this vital HMBC signal was observed in the synthetic sample.

**Fig. 8:**
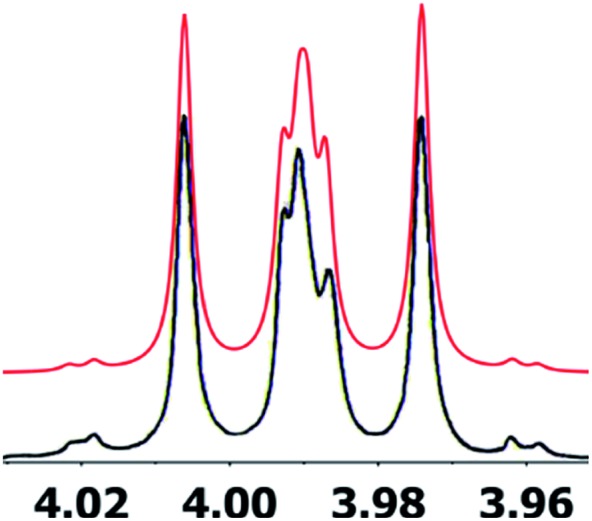
Simulation of the H2 multiplet (3.99 ppm) of aldingenin B with *J*_1a,2_ = 11.2 Hz and *J*_1b2_ = 4.8 Hz (apparent constants: 9.6 and 6.3 Hz, reported by Crimmins *et al.*^96^).

**Fig. 9:**
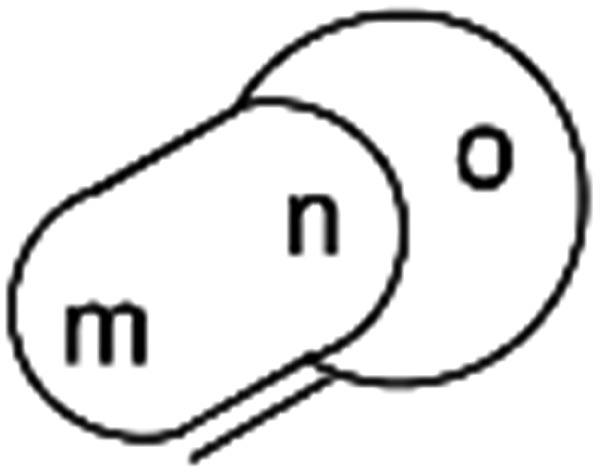
Generalisation of a caged skeleton containing a bridgehead double bond (bicyclo[m.n.o]).

A major complicating factor with analysis of the NMR data for aldingenin B was interpretation of the coupling constants for the H-1 and H-2 hydrogen signals. The H-1 signal was reported as a multiplet and the H-2 signal *J* values were misinterpreted due to their non-first-order nature. Computation of the spin–spin coupling constants for the reported structures and the proposed structure (Table 1) reveal a tight correlation of the proposed structure with the calculated values.^72^ The originally reported H-2 apparent *J*'s, 9.6 and 6.3 Hz, which are significantly different from those obtained by calculation (11.3 and 4.4 Hz), are more in line with the original bridged acetal structure, while the calculated values fit well with the proposed structure where the six membered carbocycle is more chair-like. It is noteworthy that the sum of the apparent *J'*s, 9.6 + 6.3 = 15.9 Hz, is very close to the sum of the constants obtained from the multiplet simulation (Fig. 8), 11.2 + 4.8 = 16 Hz, and that of calculated *J'*s for the proposed hemiacetal structure (11.3 + 4.4 = 15.7 Hz; Table 1; Fig. 9).

**Table 1:** Experimental and calculated ^1^H,^1^H coupling constants (*J* in Hz) of aldigenin B (**12**/**13**)^*a*^

	Match		Match	
	Exp. *J*'s (ref. 54), natural aldingenin B	DU8-calcd *J*'s hemiacetal 13	DU8-calcd *J*'s aldingenin B	Exp. *J*'s^*b*^ synthetic aldingenin B
1	m (overlap)	14.8, 8.8, 4.4	14.2, 2.5, 2.4	14.5, 2.4, 2.2
		14.8, 11.3, 8.5	14.2, 3.7, 2.0	14.5, 3.8, 2.1
2	dd (9.6, 6.3)^*c*^	11.3, 4.4	2.5, 2.0	2.5, 2.0
	11.2, 4.8			
4	dd 14.5, 9.6	14.6, 9.6	14.1, 8.1	13.7, 7.9
	dd 14.5, 4.7	14.6, 5.2	14.1, 7.2	13.7, 7.5
5	ddd 9.6, 8.4, 4.7	9.6, 9.0, 5.2	8.1, 7.2	8.1, 7.5
6	dd 9.0, 8.4^*d*^	9.0, 8.8, 8.5	3.7, 2.4	br.s.
9	t 13.5	13.4, 12.9	13.1, 12.8	13.0, 12.6
	dd 13.5, 3.6	13.4, 4.6	12.8, 4.9	12.6, 4.6
10	dd 13.5, 3.6	12.9, 4.6	13.1, 4.9	13.0, 4.6

^*a*^Calculated *J*'s are listed in descending order with a cutoff value of 2 Hz.

^*b*^For consistency, an experimental ^1^H NMR spectrum of aldingenin B in CDCl_3_ was used.

^*c*^Second order multiplet, simulation gives 11.2, 4.8 Hz with these simulated constants, calculated *J*'s for hemiacetal **13** match the experimental with rmsd = 0.46 Hz.

^*d*^It seems that this ddd (pseudo-quartet) was misreported as dd in ref. 71.

Had the raw electronic FID been available, once the original structure was in question, a reanalysis could have revealed the incorrect interpretation of the H-1, H-2 coupling constants and significantly simplified the structural revision. This case further exemplifies the clear need for thorough and careful analysis of NMR spectra when assigning structure and highlights the need to look past first order analysis of ^1^H NMR data. This example demonstrates the continued need for synthetic (or X-ray crystallographic) verification of structure and illustrates the power of computational methods in structural assignment.

A major part of the theme of this review is the need to be able to extract all of the data pertaining to a proposed structure, especially from ^1^H NMR spectra. However, in the context of the structures discussed here, it is critical to emphasize that NMR-centric elucidation work does not exclude the need to examine other data, in particular data related to the molecular formula. It is obvious that the initial investigators^71^ did not critically consider the mass spectrum, by quoting an HR-EIMS of 346.0748 and not considering the challenges associated with the EIMS of highly halogenated compounds.

### 3.6 Clearing the literature of blatantly incorrect natural product structures

NPs present a colorful palette of functional groups, and it is indeed difficult to find totally “abiotic” combinations of atoms, at least between those unreactive with water, the milieu of life. Phosphines and azides are among the most remarkable examples, but unusual functional groups that are unprecedented or very rarely documented in synthetic compounds can occur as NPs. One such case is that of β-lactam antibiotics: at the time of their original structure elucidation, it took long to dispel the proposal of considering them being oxazole derivatives.^75^ While it is, in principle, possible that NPs could “anticipate” the existence of some functional groups or combination of functional groups overlooked by synthesis or by the known biosynthetic pathways,^76^ formulas that are chemically impossible or too unstable for isolation are still reported as NPs, despite continuous and significant advances in spectroscopic techniques.

Correction sometimes requires only basic knowledge of organic chemistry. For example, the doubling of NMR resonances in the spectra of the amide **14** was ascribed to equilibration with its “isomer”, **15**.^77^ The latter is actually a resonance form of **14**, and the equilibration process detected in the NMR is what has to be expected for the rotameric interconversion of *E*-and *Z*-amide stereoisomers. Also doubtful is the isolation of the acyl chloride **16**, since this functional group is unstable in water and unlikely to exist in Nature.^78^
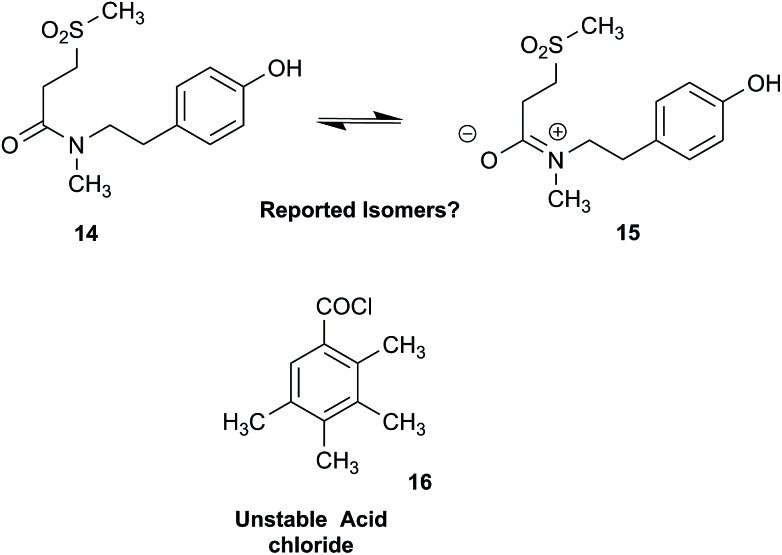


In other cases, correction can be achieved *via* re-analysis of the NMR data, which typically requires the raw NMR data to be available. Several examples exist, such as folenolide (**17**)^79^ which violates Bredt's rule; the “isoprenoid” core of the antifungal **18**,^80^ which is geometrically impossible in any isomeric form; or the *trans*-cycloheptene structure assigned to the peroxide, **19**.^81^ A re-evaluation based on the tabulated data of chemical shifts, coupling constants, and 2D correlations can lead to a successful revision.^82^ However, this kind of re-evaluation is generally difficult as documented spectroscopic assignments can be biased, as “problematic” signals might have been overlooked originally, or entire sets of signal have been misassigned. As a result, even with the availability of a synthetic version of the alleged formula, comparison of tabulated NMR spectroscopic data alone is insufficient for a structural revision, leaving the issue unsettled. The availability of the original FIDs would make such revisions possible without the need of synthesizing a non-existent NP.^19^ This would accelerate correction of wrong structures and minimize their appearance *via* peer review by making the NMR data fully transparent to peers, reviewers, editorial teams, and subsequently to readers.
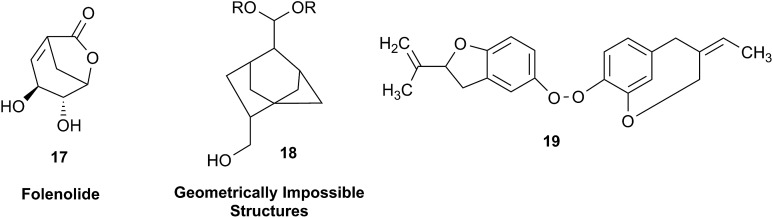


### 3.7 Bredt's rule as a check on structure correctness

Research into the structure determination of monoterpenes by Julius Bredt in the late 1800's, early 1900's, gave rise to the term Bredt's rule. This rule states that the terminus of a double bond can not exist at bridgehead positions (*i.e.*, branching position) of a bridged bicyclic system (Fig. 9).^83^–^85^ Interestingly, however, it was the physical organic chemistry community that laid out empirical guidelines for anti-Bredt systems,^86^–^91^ which became the holy grail of synthetic chemists for decades. Meanwhile, the area became somewhat foreign to the NP community. Classification of NPs with a bridgehead double bond as anti-Bredt or not was difficult, because the underlying aspect of Bredt's rule was stability and the large majority of NPs are stable.^92^ The Williams group became intrigued with a report by Cong *et al.*, reporting the isolation of neoveratrenone (**20**).^93^ The structure presented caught their attention because it contained a bicyclo[3.3.1] moiety with a bridgehead double bond. Although, the parent bicyclo[3.3.1] anti-Bredt system had been previously synthesized it was reported to be unstable. It was possible that physical properties of the entire NP skeleton enhanced stability, or the structure had been misassigned. Williams and Savchenko^82^ turned to the elucidation data, however, only ^1^H, ^13^C, HMBC and NOESY NMR data were presented in the article, with no ESI† available (*i.e.*, no additional 1D and 2D NMR data). Without the full gamut of 1D and 2D digital data, considerable detective work was required to interrogate the proposed structure. Nevertheless, they were able to reassign the structure of neoveratrenone, as **21**, based on a combination of the available data, comparison with related synthetic analogues (*e.g.*, **22**, and the co-isolation of verapatuline (**23**)) by Cong *et al.*^82^ The latter lending substantial biosynthetic support to the proposed reassigned structure **21**.
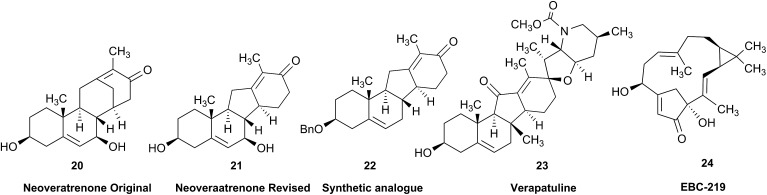


By serendipity they later isolated EBC-219 (**24**), containing a bridgehead double bond, but in a larger macrocycle.^94^ This led Krenske and Williams to develop *in silico* parameters based on olefin strain (OS) energies that now enable the NP community to cross check the validity of NPs that are proposed with bridgehead double bonds.^95^

### 3.8 Correct analysis of coupling constants

As fast and accurate computational methods become available to organic and NP chemists, there is an increasing demand for high quality NMR data available for examination and processing in different ways. The need for raw FID data is most pressing in 1D ^1^H NMR spectra, where signal overlap and second order effects often present challenges in transcribing the complexity of the spectra into neat tables. Often complex multiplets are interpreted with oversimplification. Few research groups report nuclear spin–spin coupling constants (SSCCs) with due diligence and high precision. It is common to encounter a doublet of doublets with two SSCCs differing by as much as 2 Hz or more described as a triplet with an average coupling constant reported. The accuracy of computational predictions of SSCCs has reached 0.3–0.5 Hz.^96^–^98^ Often, one faces a situation where a difference in 1–2 Hz is the only criterion for differentiating between two candidate structures. Computations may provide the answer but, without experimental data reported with appropriate accuracy, this becomes a moot point. In addition to this, typos and other errors made in the process of transcribing spectra into publication tables are inevitable, while the low quality images of these spectra in the ESI† section do not help, and serve mostly as a quality/purity control.

An example is the zoanthamine-type alkaloid 5α-iodozoanthenamine (**25**), from *Zoanthus kuroshio*.^99^ DU8+ computations^22^,^23^ of its NMR spectra identified irreconcilable differences between the computed and the experimentally reported ^1^H SSCCs, implying a misassignment. However, the predicted ^13^C NMR chemical shifts satisfactorily matched the experimental values. Closer examination of the SSCCs from a 600 MHz experiment revealed that many of them deviate from the calculated values by a factor of 1.5. For example, the constants for H-1 through H-14a needed multiplication by 1.5 to reconcile them with the computed values; H-14b did not need such correction, while most of the remaining SSCCs needed it again. As the ^1^H NMR spectra for several alkaloids reported in this paper were run at either 600 or 400 MHz, it was hypothesized that a “clerical” error had been introduced by measuring the line spacing on a hard copy spectrum and multiplying it by the wrong working frequency of the spectrometer. Revisiting the raw FID data with NMR processing software would have alleviated all problems.
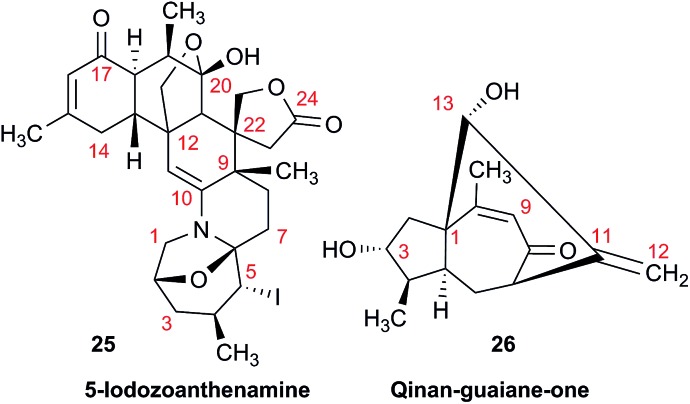


Qinan-guaiane-one, (**26**) a guaiane sesquiterpene isolated from *Aquilaria sinensis*,^100^ is another representative example where raw NMR data would have helped alleviate confusion with structure assignment. The reported geminal spin–spin coupling constant *J*_6a–6b_ = 10.3 Hz differs from the calculated value by almost 2.5 Hz (*J*_calc_ = 12.7 Hz). This error is probably not a typo, but rather it is due to the fact that the multiplets are not first order and therefore more sophisticated line fitting of the multiplets is needed to extract the actual SSCCs here. Qinan-guaiane-one is also an instructive example of the importance of accurate determination of small constants. The signal for H-13 is accurately described as a 2.3 Hz triplet. It does not have vicinal neighbors and therefore the configuration of the C-13–OH group is more difficult to assess. Luckily, the calculated allylic H-13–H-22 SSCCs for the correct (shown) stereoisomer, 2.4 and 2.1 Hz, are much closer to the reported experimental value of 2.3 Hz than the calculated allylic constants for the alternative epimer at C-13, 0.51 and 0.54 Hz. The combined evidence, together with a good match of ^13^C NMR chemical shifts (rmsd = 1.44 ppm) indicate that the originally reported qinan-guaiane-one structure is correctly assigned, but the discrepancy in the calculated and experimental values for geminal *J*_6a–6b_ is most likely due to second-order effects which are not accounted for in the authors' reporting the apparent value for this constant.

Another common problem is misinterpretation of multiplet shape in ^1^H NMR spectra. The terpene metabolite, ansellone C (**27**) was isolated from the marine sponge *Clathria gombawuiensis*.^101^ A multiplet belonging to H-19, critical for the determination of the configuration at the fusion of rings C and D, was reported as a dd 8.5 and 4.6 Hz, while the calculated values were 4.7 and 4.3 Hz. In the copy of the spectrum in the ESI,† this multiplet does not look like a dd 8.5 and 4.6 Hz, but it is virtually impossible to extract any useful information from the picture. In summary, the configuration of ansellone C (**28**) is either misassigned or the H-19 multiplet is interpreted and reported incorrectly. Raw FID data would have helped to resolve this issue.
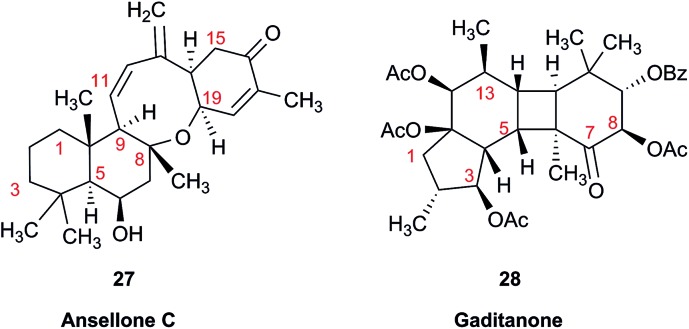


In general, ^13^C NMR spectra are less prone to the problems outlined above, but even there one sees occasional misinterpretation of an impurity signal and typos in transcribed tables of chemical shifts are plentiful. For example, a complex diterpenoid, gaditanone, (**28**) possessing an unprecedented 5/6/4/6-fused tetracyclic ring skeleton, was recently isolated and characterized by solution NMR,^102^ with its only carbonyl carbon, C-7, assigned the chemical shift value of 206.6 ppm. The DU8+ calculated value for this carbonyl carbon is 213.8 ppm, indicative of misassignment. However, a cursory look at the copy of the spectrum in the ESI† revealed an unannotated extra signal at 29–30 ppm, implying that acetone is an impurity in the sample. It is plausible that the actual carbonyl signal belonging to **28** was overlooked as it was too small. Exclusion of the carbonyl signal from the statistics improves the match of the experimental and computed ^13^C NMR chemical shifts to rmsd = 1.23 ppm. This excellent accuracy leaves no doubt that the structure of the diterpenoid is correctly assigned. It also suggests that the authors should examine the vicinity of 212–214 ppm for the actual carbonyl signal belonging to gaditanone (**28**).

### 3.9 Sulfones *vs.* sulfinates

Chemical investigation of an Australian sponge, *Aplysinella rhax*, led to the isolation of psammaplins A, I, and J.^103^ Psammaplin I (**29**) was first isolated from *Pseudoceratina purpurea* and formulated to contain a sulfone moiety, from IR data.^104^ The metabolite was later reported from a *Jaspis*/*Poecillastra* sponge association without additional comment on its structure.^105^ The first published NMR data reported the H-2 signals at 2.96 ppm (m) and 3.75 (s), and H-3 as a triplet of doublets (td) centered at 3.62 ppm with *J* values of 6.5 and 2.0 Hz. Sulfones are not normally chiral since two of the substituents attached to sulfur are oxygen, therefore each set of the methylene hydrogens at C-2 and C-3 should have been equivalent. Data acquired at 500 MHz in CD_3_OD by the Garson group revealed diastereotopic ^1^H multiplets at 2.91 and 3.01 ppm assigned to the H-2 hydrogens, and a complex two hydrogen signal centered at 3.62 ppm for the methylene hydrogens at C-3; these data supported a methyl sulfinate, as in **30**.
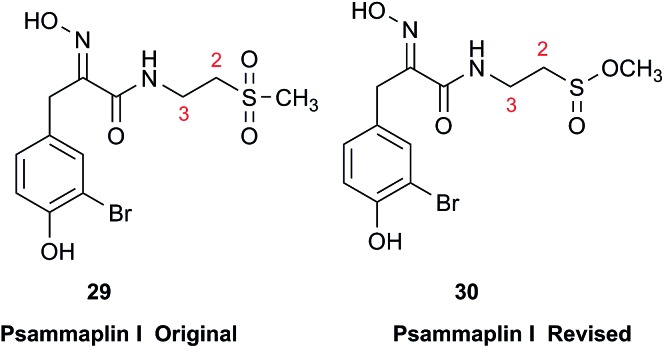


Even despite the incorrect chemical shift value originally reported for one of the H-2 signals, their data were inconsistent with a sulfone functionality. Although the (H-3)_2_ signal superficially resembled the triplet of doublets as reported, it showed ten lines on close inspection, and was best described as an AB system (3.63 and 3.61 ppm) in which each line is split into a triplet by two vicinal couplings of ∼6 Hz. Owing to signal overlap, only ten of the predicted twelve lines were resolved. Repeated acquisition of the ^1^H NMR data at 900 MHz confirmed the complexity of the H-3 and H-2 signals. At 500 MHz, the two chemical shifts for H-3 were calculated as 3.630 and 3.614 ppm with ^2^*J* = 14.8 Hz, and at 900 MHz as 3.631 and 3.615 ppm with ^2^*J* = 14.9 Hz. Detailed modeling of the H-2 and H-3 spin systems was carried out on the 900 MHz spectrum of psammaplin I (**29**). The signal at 3.75 ppm for the OMe group of the methyl sulfinate had been incorrectly assigned to H-2; however, the signal integrated for 1.8H owing to partial transesterification by the NMR solvent.

Concurrently with the above NMR study, the Ireland group independently prepared two methyl sulfinate ester derivatives of psammaplin A, one of which had spectroscopic data identical to psammaplin I.^106^ However, their ^1^H NMR data were run at 500 MHz, as were the original data,^104^ so the nonequivalence of the H-3 hydrogens that resulted from the presence of the chiral sulfur atom in psammaplin I may not have been evident.

This case study highlights the valuable role of very high field NMR in the dereplication of marine NPs. When chemical shifts and coupling constants are reported accurately, the values can be compared for a sample run at any field strength.

The prediction of chemical shift values by quantum chemical methods has provided valuable insights into NP structures, including the correction of published structures. The Garson group recently revised their published structure for acremine P, a metabolite of *Acremonium persicinum*, following a comparison of calculated and experimental NMR chemical shift data.^107^ When the originally published structure, **31**,^108^ was examined using a combination of computational approaches that provide ^13^C NMR shifts with mean absolute error (MAE) of ∼1.6 ppm, there were deviations of 20.4 ppm for the alkene carbon (C-2) and –23.0 ppm for the hydroxymethine carbon (C-7). Re-evaluation suggested the signal at 95.0 ppm (C-7) had been incorrectly assigned to a secondary alcohol instead of an acetal or lactol. Furthermore, the alkene carbon signals (102.4 and 162.5 ppm) indicated a polarized double bond, likely enolised given the number of oxygen atoms in the molecule. HMBC correlations of both the lactol hydrogen at 5.83 ppm (d) and the signal at 4.15 ppm (s) for the hydroxymethine hydrogen H-8 to the acetal carbon at 99.0 ppm supported the revised planar structure, **32**.
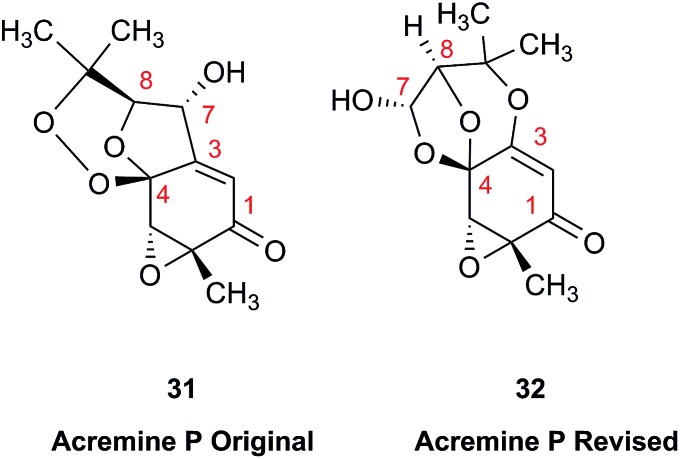


DFT computations did not safely distinguish between four proposed diastereomers of acremine P owing to the close similarity of the calculated ^13^C NMR shift values. The calculated chemical shifts were further examined using the DP4+ computational approach developed by Sarotti *et al.*^109^ to assign the most probable diastereomer.^109^ Using the ^13^C NMR data alone, the probability was 99.7% that **32** was the correct diastereomer. Coupling information, notably the zero coupling between the vicinal lactol and hydroxymethine hydrogens, as well as *J*_H7–H8_ couplings calculated for each stereoisomer using the methods of Kutateladze *et al.*,^98^ together with NOE data further supported the relative configuration shown.

Garson *et al.* had earlier reported that hydrogenation of acremine P yielded acremine A as the sole product;^108^ clearly structure **31** could not be correct as the dioxolane ring of the revised structure was incompatible with the tetrahydrofuran ring previously ascribed to acremine P. The revision of the structure of acremine P highlights the valuable role of computational studies in evaluating the structures and configuration of complex NPs. In each of these cases, the original FIDs of both the ^1^H and ^13^C spectra can provide a basis for quantum mechanical analysis and a rapid resolution of the structural assignment problems.

### 3.10 Methylene signal assignments in the structural revision of aromin to montanacin D

The originally proposed structure of aromin (**33**)^110^ an Annonaceous acetogenin,^111^,^112^ was revised recently to be montanacin D (**34**) by total synthesis of the proposed structure of aromin,^113^ and re-examination of NMR data of synthetic montancin D^114^ and other related isomers,^115^ especially ^13^C NMR data using CAST/C NMR Structure Elucidator,^116^ and MS fragmentation analysis of TMS derivatives of **33** and **34**.^113^
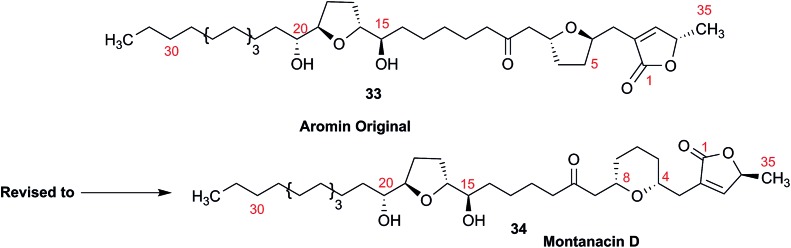


**Fig. 10:**
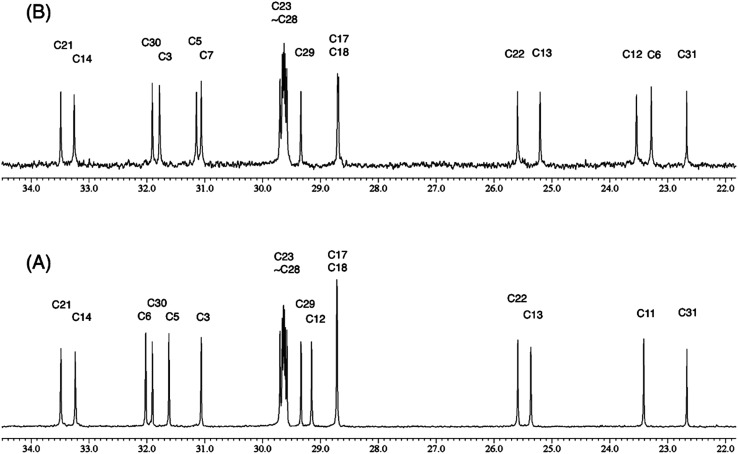
^13^C NMR spectra (150 MHz) of methylene region for (A) the originally proposed structure for aromin (**33**) and (B) montanacin D (**34**). Region for two methylenes at α-positions of ketone carbonyl group was omitted (48.74 (C8), 43.42 (C10) for **33**, and 49.13 (C9), 43.76 ppm (C11) for **34**, respectively). Spectra were measured in CDCl_3_ solution at 25 °C. Assignments were carried out by analyses of several 2D experiments including HMBC data.

When comparing originally reported NMR data and synthetic compounds, ^13^C NMR data with a tabulated ^13^C NMR chemical shifts of aromin and montanacins were insufficient because exchangeable methylene signals were lumped together in the region of 31.1–31.9 ppm for C-3, C-5, and C-6 of aromin,^110^ and in the wide range chemical shifts such as 23.4–31.9 ppm for thirteen carbon signals in the case of montanacins D and E.^117^ Complete assignments of severely overlapped methylene signals in ^1^H and also ^13^C NMR were difficult or impossible in some cases, but relevant information of exact chemical shift values, number of signals, and intensities of them are very important for comparison of NMR spectra directly among NPs and synthetic compounds. Fig. 10 shows the ^13^C NMR spectra for methylene regions of synthetic montanacin D and the proposed structure of aromin are shown. From the viewpoint of structural revision of aromin, the assignment of 23.28 ppm for C-6 of **34** is critical as the methylene signal at the γ-positions from the ether oxygen in the tetrahydropyran ring, which is absent in the spectra of the synthetic **33**. In comparison between ^13^C NMR data of **33** and **34**, assignment of 29.15 ppm for C-12 at the γ-position from the carbonyl group at C-9 and the δ-position from the hydroxyl group on C-15 will be important to determine the methylene chain length between C-9 carbonyl and C-15 hydroxyl groups. Together with these assignments, signal assignments of C-3, C-5, and C-6 for **33**, and C-3, C-5, and C-7 for **34** are important to characterize the partial structure of the tetrahydrofuran or tetrahydropyran ring system, respectively. Exact ^13^C chemical shift values could be obtained from raw NMR data readily and, *e.g.*, are useful for direct comparison and to create database queries for the CAST/C NMR system. For acetogenins, ^1^H NMR raw data of intact compounds are also useful, but also raw data of MTPA ester derivatives are very important. These are required to determine the absolute configuration of hydroxyl groups and relative configurations of separated chiral centers.^114^,^115^

### 3.11 The case of aglalactone

The NMR based elucidation process generally follows two major strategies, or a combination thereof. One strategy involves spin–spin interactions utilized to generate different spin networks, the other is focused on chemical shifts and relies on increment rules. The number of experimental data-points (chemical shifts, *J*-couplings, dipolar couplings) outnumbers the atoms present in a molecule by far. This results in a high number of degrees of freedom, and successful structure assignment requires a combination of two major thought processes in parallel: intuitive interpretation of all available data, and knowledge of NP biosynthesis. Also, wherever expert reasoning is part of a research strategy, errors are well within the realm of possibility.

When investigating the genus *Aglaia* in the late 1990s, Hofer and colleagues came across a molecular scaffold that was unusual for the Meliaceae: a benzofuranone lactone congener named aglalactone. It was determined to bear a lactone moiety and appeared to fit well into the biogenetic reasoning for far more complex compound classes such as the panellins or flavaglins.^118^,^119^ Integrated analysis of HR-MS, IR, and NMR data was straightforward and led to the assignment of structure, **35**. “Missing” NOE contacts were explained by configurational and spatial considerations. However, when re-investigating the aglalactone ^13^C NMR data by means of the CSEARCH database (see also Section 8.2),^120^,^121^ it became evident that a single ^13^C NMR shift value (a CH element resonating at about 81 ppm) showed a significant mismatch relative to the calculated value. Hence, a reinvestigation of the structure elucidation process was commenced. An alternative hypothesis was generated and a set of possible regional isomers formulated. Independent acquisition of additional spectroscopic evidence on a re-isolated analyte was key for this strategy. After time consuming procurement of the analyte, the generation of a complete NMR data set including HMBC and NOE spectra as well as a lanthanide induced shift (LIS) NMR data sets were recorded. The new data strongly supported a new structural hypothesis, **36**, which was based on “inversion” of the lactone moiety. Subsequently, the structure and the scaffold ring system were revised from a 2,3-dihydrobenzofuran-2-one to a 3*H*-isobenzofuranone.^122^ Within the past decade, the isolation of aglalactone from several sources and the discovery of an additional congener^123^–^125^ represent an independent and strong confirmation of the scaffold correction undertaken by Seger and colleagues.
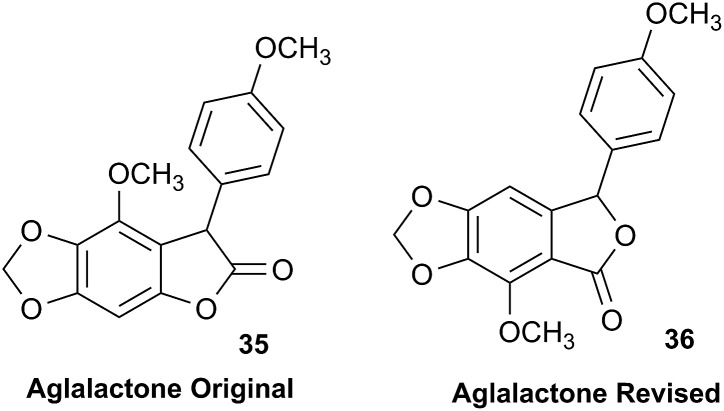


Although NMR data from the original investigation were available at the time of the aglalactone revision, the data set was deemed incomplete, as HMBC data was unavailable. While it was possible to re-isolate the compound, the group of Hofer and Greger experienced further difficulties. In one instance, a collaborative effort was necessary,^126^ in another case, only total synthesis was able^127^ to correct a structure.

Almost two decades after the structural revision, the correct structure of aglalactone is still not disseminated properly to the scientific community, including in major resources and databases. Notably, if NMR raw data sets would be available and become a routine part of deposited data, it would be straightforward to correlate the structures and (different) structural proposals *via* their fingerprint NMR spectra, independent of the limitations of spectral figures in publications and their ESI.† Furthermore, such raw data would enhance the traceability of any novel congener claims relative to the first reported congener of a given compound class. In such instances, a series of NMR data signals would typically show close matches between the congeners, thereby proving unequivocally the relationship of the compounds *via* spectral similarity. This kind of “similarity feature” can be transferred from the analogue world of expert reasoning to computer based similarity searches. The approach is already very well-known from other research fields such as the LC-MS^n^ or GC-MS/MS based general unknown screening (GUS) in toxicology^128^ or the spectral feature comparison approaches, followed by IR/NIR based applications in clinical chemistry or forensics.^129^

### 3.12 Diastereoisomers and rotamers

^1^H NMR spectra provide not only two-dimensional structural information but also very detailed configurational data on NPs, *e.g.*, epimers or rotamers, which have been many times neglected, as they were considered as signals due to impurities and are thus not reported in the final discussion on the structure elucidation. Some flavanone glycosides and flavone-8-*C*-glycosides exemplify this problem.

In the past, flavanones were believed to occur in nature as levo-rotatory (2*S*)-isomers because the enzyme catalyzing the conversion of chalcones to flavanones is highly stereospecific.^130^ However, flavanones and their glycosides are present as enantiomeric and diastereomeric mixtures, respectively. Among others due to ring-opening of flavanones under basic conditions^131^ or instability and rapidly recyclization to flavanones in a non-stereospecific manner.^132^ In the case of the aglycone of a flavanone, naringin, the presence of stereoisomers cannot be observed in the ^1^H NMR spectra because naringenin has only one chiral center (C-2), so the two enantiomers have identical spectra. The attachment of a sugar yields various glycosides and these represent the most abundant form of naringin in nature. However, the introduction of one or more other enantiomeric centers results in a mixture of different diastereoisomers with different chemical properties and thus also different NMR spectra. Similar to the naringin case, the ^1^H NMR spectra of other flavanone glycosides, like hesperidin and neohesperidin are characterized by the clear presence of signals of two diastereoisomers.^133^ In the ^1^H NMR spectrum the ratio between diastereoisomers is easily calculated from the raw ^1^H NMR data. For example, in the case of neohesperidin the ^1^H NMR spectrum shows a bigger difference in the ratio of the two stereoisomers of the molecule (1 : 4 between two isomers), as compared to naringin (2 : 3 between two isomers).

Another group of isomers with different chemical properties, are the rotamers which are generated from conformational isomerism, in which the rotamers cannot easily be interconverted by rotation around a single bond. In nature, many 8-*C*-glycosides of flavonoids are often found to have rotamers due to steric hindrance at the *C*–*C* glycosyl flavone linkage.^134^ In the case of vitexin, the chemically equivalent H-2′ and H-6′ hydrogens show two broad signals due to rotamers. In the ^1^H NMR spectrum of orientin, another flavonoid 8-*C*-glycoside, signal broadening is detected around 7.5 ppm (H-2′) because of the presence of rotamers. However, the isomers isovitexin and isoorientin with *C*-glycosidic sugars at C-6 do not show the presence of rotamers.

It is generally accepted that plant metabolites are produced in a stereospecific way because of the involvement of enzymes in many biosynthetic steps. However, different stereoisomers of the same compound may exist in nature, either as side-products of an enzymatic reaction or after a chemical conversion. By neglecting minor signals in the NMR spectra of NPs, by marking them as impurities important information is lost. Not reporting the full raw data, means that later colleagues might have problems in purifying compounds as they are not aware of the extra signals due to these situations. Therefore, any paper on structure elucidation and identification of NPs, should give the full raw NMR data.

### 3.13 Data ambiguity

The marine NP, gallinamide A (**37**) was first isolated from a cyanobacterial *Schizothrix* species collected from a reef near Piedras Gallinas in 2009.^135^ The structural assignment used classical 1D and 2D NMR and mass spectrometry methods. The absolute configuration required a combination of chemical degradation and chiral chromatographic analyses. Although this was successful for most chiral centers, the absolute configuration of the terminal (*N*,*N*-dimethyl isoleucine) residue was not determined due to a lack of material, but was postulated as possessing the L configuration based on biogenetic arguments.^136^

Shortly after this initial publication, a second compound with the same planar structure was published.^136^ This new compound, symplostatin 4 (**38**), possessed the same relative configuration as gallinamide A at all the assigned chiral centers (see chemical drawings). In addition, the absolute configuration for the *N*,*N*-dimethyl isoleucine residue was determined, and reported as L. A footnote in the manuscript describing the discovery of symplostatin 4 stated that the NMR data between symplostatin 4 and gallinamide A differed significantly in the *N*,*N*-dimethyl isoleucine region, and suggested that the two compounds were therefore, logically, diastereomeric.
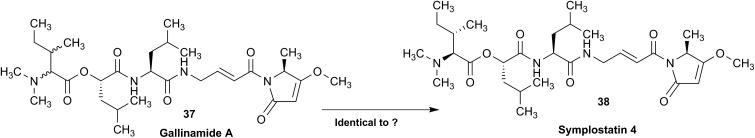


**Fig. 11:**
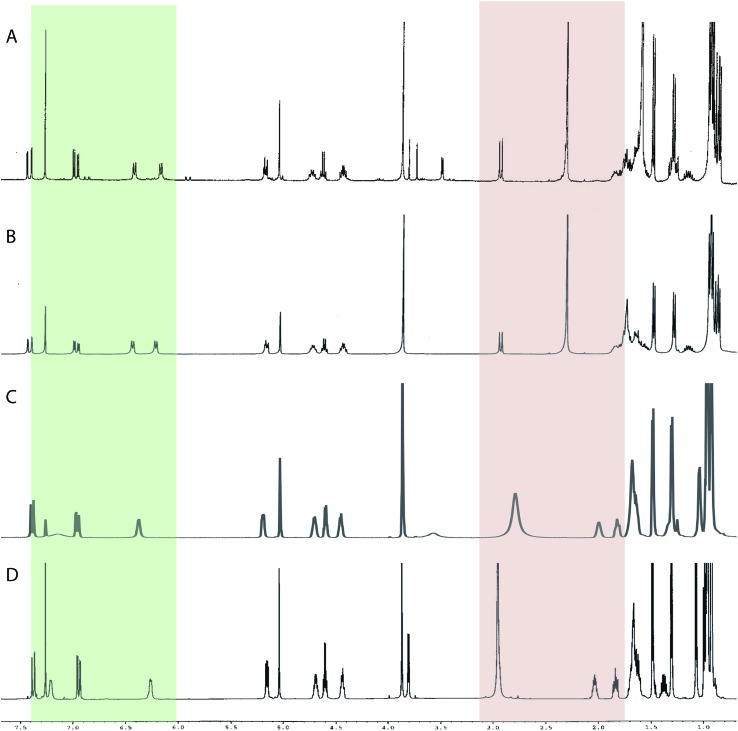
NMR profiles of (A) gallinamide A (**37**), as reported and adopted from;^135^ (B) synthetic gallinamide A (**37**) as reported and adopted from;^138^ (C) symplostatin 4 (**38**) as isolated and adopted from;^136^ and (D) synthetic symplostatin 4 (**38**) as reported and adopted from.^139^ Variations in the spectra signals in the isoleucine region (1.0 to 3.0) led to speculation that the compounds were diastereomers. Further studies showed this was not the case after investigation and direct comparison of the region (highlighted red) by Conroy *et al.*^139^ Variations in pH and/or concentration give rise to other spectral differences, such as those seen in the NH region (highlighted green). The construction of this figure demonstrates the challenge of reporting high quality, scalable comparison data without access to the original files.

Subsequently, several groups have pursued total syntheses of these structures.^137^–^140^ The first, published in 2010 reported the synthesis of symplostatin 4 and presented NMR data that differed significantly from those reported for gallinamide A, particularly in the *N*,*N*-dimethyl isoleucine region (Fig. 11, highlighted in red).^139^ Subsequently, this same group synthesized all four possible diastereomers of gallinamide A in an attempt to resolve the outstanding uncertainty about the structure of this metabolite. In collaboration with the author who originally isolated gallinamide A, all four of these compounds were subjected to full *de novo* structure elucidation, with the structures blinded to the chemist performing the structure elucidation to eliminate bias in the assignments. Surprisingly, when the resulting hydrogen and carbon chemical shift values were compared to those for the NP, the values for the l-isoleucine derivative were the only ones that matched the data from the original gallinamide A data.^138^ Although initially reported to have significant variations in the *N*,*N*-dimethyl isoleucine region, subsequent comparisons of the 1D NMR spectra in CDCl_3_ show that the variations between the spectra are minimal.^138^ Perhaps confounding the issue, the original isolation of gallinamide A was tabulated in CD_3_CN in text, but additionally provided the unannotated CDCl_3_ spectra in the ESI.† Submission of the 1D FID files would have enabled more accurate comparisons between the two spectra directly, helping to minimize ambiguity of the data (Fig. 11).

As can be seen between the spectra of symplostatin 4 from the initial and the later gallinamide A synthesis reports, and studied thoroughly in (acyclic) peptides, effects of concentration and pH have substantial impact on the spectral characteristics of compounds, even in the same solvent (Fig. 11, highlighted in green).^141^–^143^ While beyond the scope of the initial studies, providing spectra of compounds in several solvent systems and under different conditions would still enable more detailed studies into the effects of pH and concentration on the spectra of a metabolite, and provide additional tools for investigators to more accurately dereplicate compounds under a variety of conditions. Additionally, as time progresses and data processing techniques are refined, tools such as deconvolution algorithms and non-FT processing techniques could be profitably applied to retroactive analysis of existing data sets.^144^,^145^

This vignette highlights the challenges associated with determining relationships between structures from tabulated data. Had all of the original data files been available, it would have been possible to directly compare the NP samples, and relate these to the synthetic materials. Instead, exhaustive synthetic efforts demonstrated that gallinamide A possesses a structure identical to symplostatin 4 (**38**).

### 3.14 The importance of details

**Fig. 12:**
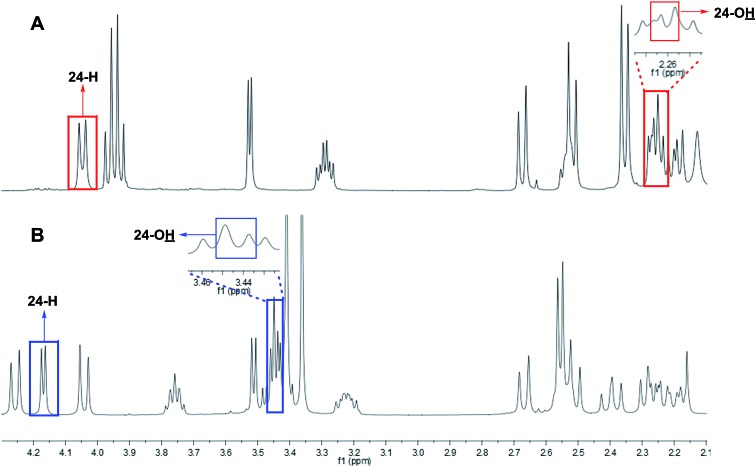
The partial ^1^H NMR spectra of phainanoid B (**39**; A) and phainanoid F (**40**; B).

**Fig. 13:**
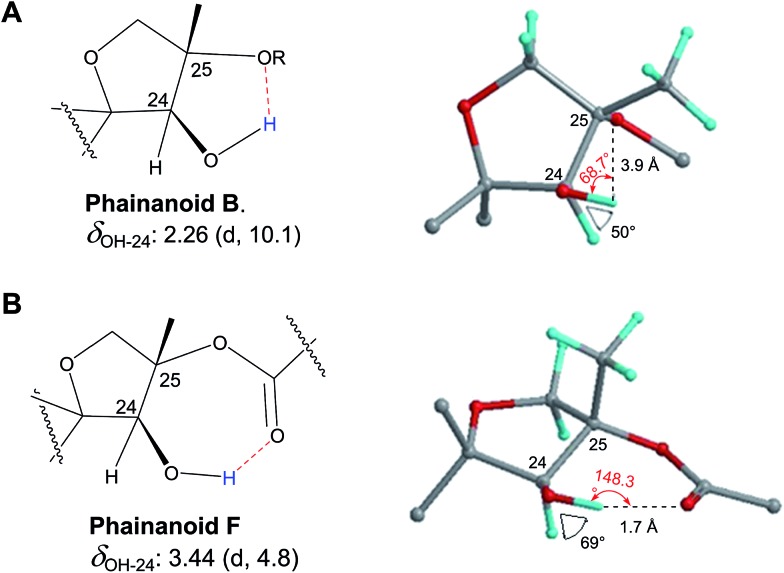
Chemical shifts (ppm) and coupling constants (Hz) of OH-24 in the phainanoids B (**39**) and F (**40**): the optimized 3D structures ((A): OMe-25 represents phainanoid B; (B) OAc-25 represents phainanoid F) generated by Hartree–Fock/3-21G showing the dihedral angles of H–C–O–H (black) and H-bond angles (red) and lengths (Å).

Detailed analysis of the chemical shifts and coupling constants can not only elucidate the fine structures of complex natural compounds, but also provide useful information to probe the formation of the different intramolecular H-bonds in very similar analogs. For instance, phainanoids B (**39**) and F (**40**), possessed similar structures except for the different substituents at C-25 (Fig. 12). However, the chemical shifts and coupling constants of the OH-24 hydrogen showed major differences (Fig. 13), which were believed to be caused by the formation of different intramolecular H-bonds with the OR-25 moieties.^146^,^147^ For phainanoid B (**39**), the OH[combining low line]-24 resonated upfield with a large coupling constant (*δ* 2.26 ppm, *d*, *J*_24,OH_ = 10.1 Hz), suggesting that the H-bond was formed between OH[combining low line]-24 and the oxygen atom of O[combining low line]H-25 in a five-membered cyclic interconnection (Fig. 13A), in which the H-bond angle and length simulated for OH-25 were ∼69° and ∼3.9 Å, respectively,^148^–^150^ and the dihedral angle between H-24 and OH[combining low line]-24 were 50° as generated by Hartree–Fock/3-21G.
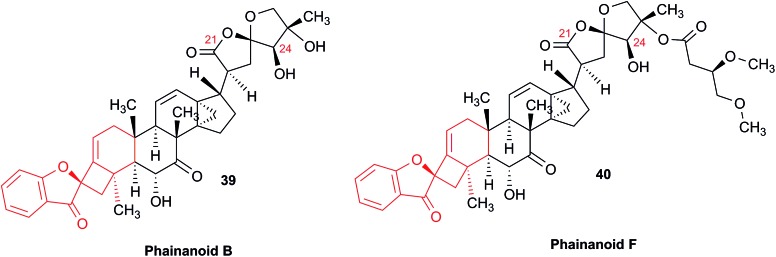


In contrast, phainanoid (**40**) showed a stronger H-bond, formed between OH[combining low line]-24 and the O-atom of the acyl carbonyl furnishing a seven-membered ring (Fig. 13B), with a more favorable H-bond angle and length of ∼148° and ∼1.7 Å, respectively.^148^–^150^ This resulted in a downfield chemical shift and a smaller coupling constant for the OH[combining low line]-24 signal (*δ* 3.44 ppm, *d*, *J*_24,OH_ = 4.8 Hz) compared with those of phainanoid B (**39**), owing to the deshielding effects of acyl group and the increased dihedral angle (∼69°). The coupling constants of H-24/OH[combining low line]-24 and the dihedral angles in the simulated conformers of **39** and **40** satisfied the Karplus equation.^151^,^152^ The other reported compounds of two subclasses with different substitution patterns at C-25 were also consistent with this interpretation.^153^ These insights became possible only *via* a full analysis of the NMR data and highlight the importance of careful analysis, especially of chemical shifts and coupling constants that together provided a useful tool for insight into the fine structures and conformations of complex NPs in solution.

### 3.15 Structural instability leads to dynamic complexity

In 2014, the Williams group disclosed the isolation of EBC-329 (**41**) and EBC-324 (**42**) from a plant collected in an Australian rainforest, together with collaborators from EcoBiotics Ltd and the QIMR Berghofer Medical Research Institute.^154^ EBC-324 (**42**) contained an unusually oxidized casbane (**43**) ring system, whereas EBC-329 (**41**) was the first example of a *seco*-casbane reported. Some years after a number of additional examples of both the *seco*-casbane [EBC-328 (**44**) and EBC-363 (**45**)],^155^ and casbane [EBC-304 (**46**) and EBC-320 (**47**)] series,^156^ were discovered.
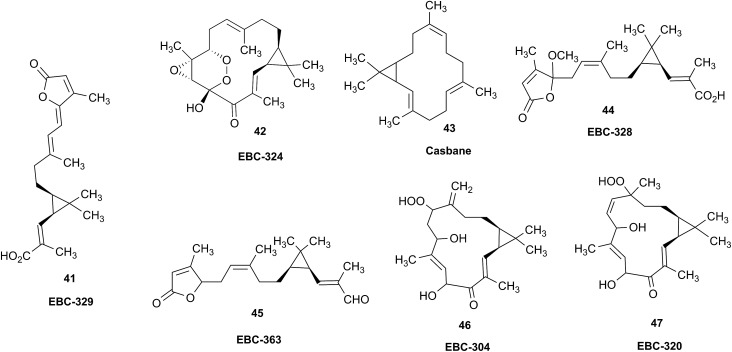


Approximately one year after reporting EBC-329 (**41**), Thombal and Jadhav described the synthesis of racemic **41** in 13 steps and 10% overall yield. However, the ^1^H NMR spectra data was inconsistent with that reported for the NP, although, the ^13^C NMR appeared to match.^157^ Unfortunately, the raw digital data was not available to analyze additional expansions that would have facilitated further understanding.

Only by chance, the Williams group was also working on the total synthesis of this molecule (*i.e.*, **41**), but lagged behind the Jadhav team by two years. However their route was superior in step count (7 steps), and was chiral, allowing the absolute configuration to be determined.^158^ It was, however, a serendipitous flaw in this route that revealed why the Jadhav *et al.*^1^H NMR spectra did not match that reported in 2014 for **41**. The deployment of the Horner–Wadsworth–Emmons olefination protocol here did not provide a high level of *E*/*Z* stereocontrol, which led to a mixture of **41** and **48**. The ^1^H NMR spectra of this mixture was a match to the Jadhav spectrum, although the ratio of **41** and **48** was different (*i.e.*, Jadhav obtained a 1 : 1 mixture).
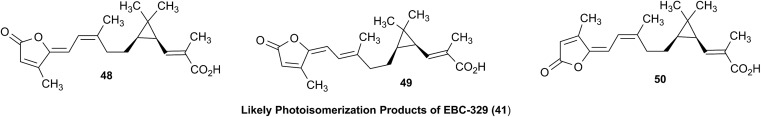


The Williams group were able to purify the target (*i.e.*, **41**) by HPLC, and discovered that the purified material photoisomerized on exposure to laboratory light, giving an isomer that matched an impurity in their spectra of an isolated sample of **41** from 2014. Although it was not possible to unambiguously determine the structure of the major impurity, it was most likely either **49** or **50**.

### 3.16 Acetogenins-the difficulty of configurational determination

Halogenated C_15_-acetogenins, containing at least 180 members, are widely present in the marine red algae of the genus *Laurencia*, and often feature one or more ether rings of different sizes.^159^ Among them, the structures of elatenyne (**51**) and its congeners, such as laurendecumenyne B (**52**), were originally assigned with a pyrano[3,2-*b*]pyran unit,^160^,^161^ but were ultimately corrected to possess a 2,2′-bifuranyl core, which contain the carbon and hydrogen connectivity of a pyrano[3,2-*b*]pyran unit.^162^ The overlapping signals in 1D and 2D NMR spectra made the structure and configuration elucidation difficult.

Elatenyne was initially isolated from *L. elata* by Hall and Reiss in 1986 and originally identified as a pyrano[3,2-*b*]pyran structure (**51**) from its NMR data.^160^ In 2007, Wang and co-workers re-isolated elatenyne as a mixture with a structurally related congener, laurendecumenyne B (**52**), from the marine red alga *L. decumbens*, and the structures and relative configurations of these two compounds were established as pyrano[3,2-*b*]pyran derivatives by referring to the original structure and NMR data of elatenyne.^161^ Later in 2010, the structures were revised to **53** and **54**, respectively, as being 2,2′-bifuranyl derivatives by Wang and co-workers,^162^ based on the total synthesis and the ^13^C NMR calculations reported by Burton and co-workers.^163^,^164^ However, the dibrominated 2,2′-bifuranyl structure, was assigned as a diastereomer of elatenyne, because the ^1^H NMR data recorded in CDCl_3_ appeared different.^162^ Later in 2011, Dias and Urban obtained elatenyne from *L. elata* and recorded its ^1^H and ^13^C NMR spectra in both CDCl_3_ and C_6_D_6_, which indicated that the originally reported ^1^H NMR signals of elatenyne in CDCl_3_ were incorrect and confirmed that the dibrominated 2,2′-bifuranyl metabolite obtained by Wang and co-workers was indeed elatenyne.^165^
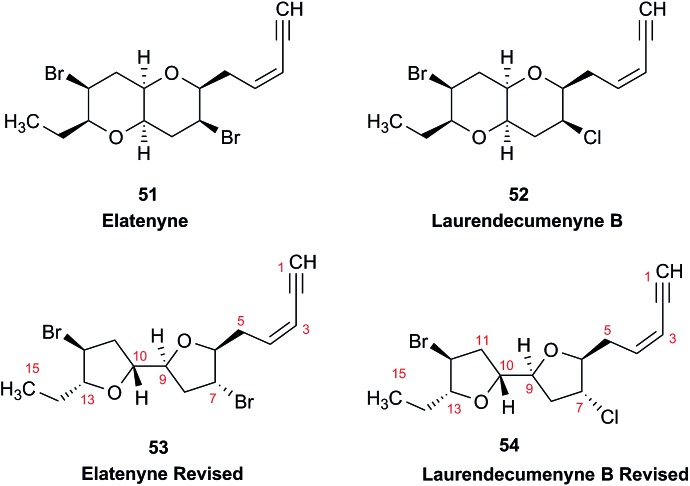


The most likely structure (**53**) for elatenyne produced by DFT calculations of GIAO ^13^C NMR and its enantiomer were totally synthesized by the Burton and Kim groups in 2012, and their NMR spectra were compared with the raw spectra of the isolated elatenyne, despite the unmatched specific optical rotation values.^166^ Simultaneously, the relative configurations of the revised laurendecumenyne B (**54**) and (*E*)-elatenyne (**55**) were also confirmed by total syntheses,^166^,^167^ and the former was further evidenced to be a stereoisomer of notoryne (**56**) that was determined by NMR, EIMS, and chemical degradation methods.^168^ The ^13^C NMR signals of synthetic elatenyne, laurendecumenyne B, and (*E*)-elatenyne (**55**) were usually in good accordance with those of corresponding isolates. However this was not always the case for the ^1^H NMR data when the reported data was carefully rechecked.^160^,^161^,^165^–^167^ The splitting patterns and coupling constants of H-9 or H-10 are key to elucidate the relative configuration between the two tetrahydrofuran rings, and they should be the same or similar in view of the identical configurations around these two positions in **53–56**. However, most of the isolates and synthetics (**53–56**) were reported to possess incongruous splitting patterns and coupling constants of H-9 or H-10, as summarized in Table 2. Thus, it is possible that either the coupling constants were calculated inaccurately or the relative configuration between the two tetrahydrofuran rings was assigned incorrectly. This is difficult to clarify with only printed ^1^H NMR data, and would be achievable with raw or at least digital shared data.
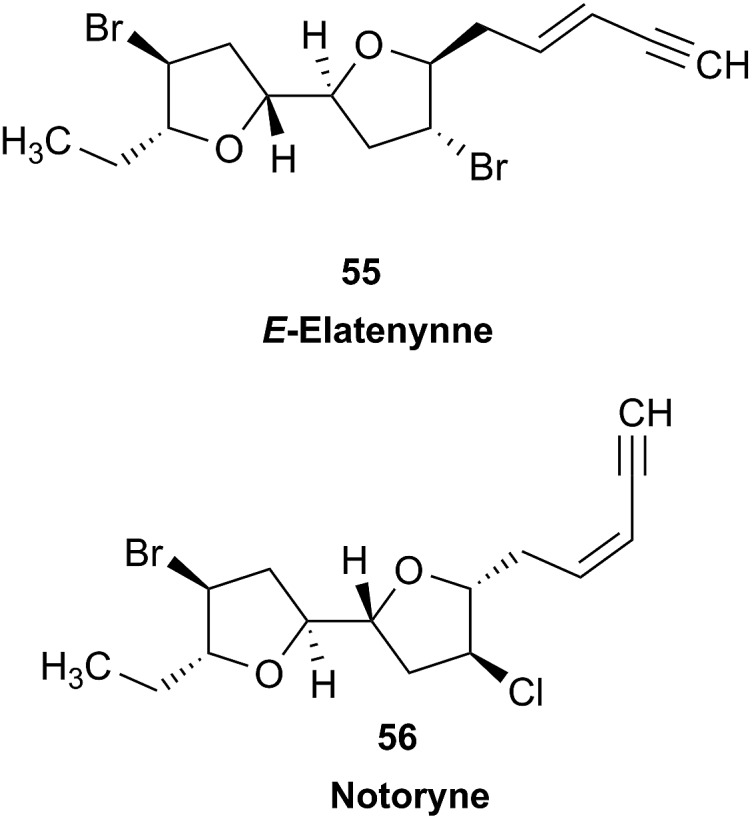


**Table 2:** The ^1^H NMR resonances of H-9 and H-10 of elatenyne (**51**/**53**) and its congeners (*δ* in ppm)

Compound	Solvent	Frequency [MHz]	*δ*_H-9_ (*J* in Hz)	*δ*_H-10_ (*J* in Hz)	Ref.
Elatenyne (**51**)	C_6_D_6_	199.5	3.84, m	3.84, m	160
**51**	CDCl_3_	500	4.15, m	4.15, m	161
**51**	C_6_D_6_	500	3.86, m	3.86, m	165
**51**	CDCl_3_	500	4.15, ddd (12.0, 7.0, 5.5)	4.15, ddd (12.0, 7.0, 5.5)	165
Elatenyne (**53**)	C_6_D_6_	500	3.84–3.93^*a*^, m	3.84–3.93^*a*^, m	166
**53**	C_6_D_6_	200	3.79–3.97^*a*^, m	3.79–3.97^*a*^, m	166
**53**	CDCl_3_	500	4.17, ddd (12.0, 6.8, 5.5)	4.17, ddd (12.0, 6.8, 5.5)	166
**53**	CDCl_3_	200	3.91–4.29^*b*^, m	3.91–4.29^*b*^, m	166
Laurendecumenyne B (**52**/**54**)	CDCl_3_	500	4.15, m	4.15, m	161
*ent*-**54**	CDCl_3_	500	4.15, m	4.15, m	166
(*E*)-Elatenyne (**55**)	C_6_D_6_	400	3.75, dddd (7.0, 6.9, 6.8, 0.6)	3.79, dddd (7.1, 7.0, 6.8, 0.6)	167
**55**	C_6_D_6_	500	3.73–3.83, m	3.73–3.83, m	166
**55**	C_6_D_6_	400	3.73–3.83, m	3.73–3.83, m	166
*ent*-**55**	C_6_D_6_	500	3.82, dddd (12.9, 12.9, 6.4, 6.4)	3.82, dddd (12.9, 12.9, 6.4, 6.4)	166
Notoryne (**56**)	CDCl_3_	400	4.26, ddd (7.3, 7.3, 5.5)	3.98, ddd (8.3, 6.8, 5.5)	168

^*a*^Overlapping signals with H-13.

^*b*^Overlapping signals with H-6, H-7, H-12, and H-13.

**Fig. 14:**
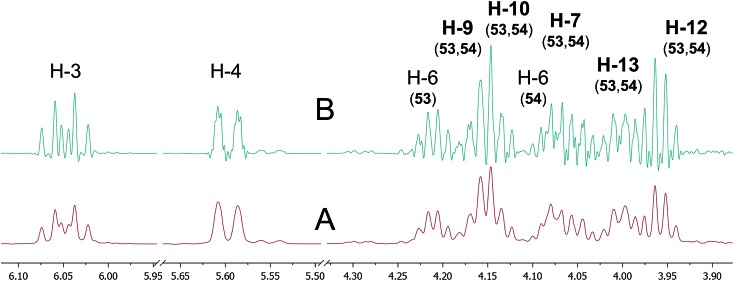
Comparison of the ^1^H NMR signal splitting patterns of a mixture of elatenyne (**53**) and laurendecumenyne B (**54**) with different post-acquisition processing. Spectrum A shows the typical “standard” processing with exponential multiplication (EM) using an LB value of 0.3 Hz. Spectrum B was generated from the same FID in two steps: reference deconvolution for a 1.0 Hz lineshape optimization, followed by Lorentzian–Gaussian windows function (LG; LB = –2.2 Hz, GB = 0.25) for resolution enhancement. Both spectra were zero filled to 128*k* real data points. The resolution enhanced spectrum B allows a more consistent assignment of multiplicities and resonance locations, in particular for the key signals of H-9 and H-10.

**Fig. 15:**
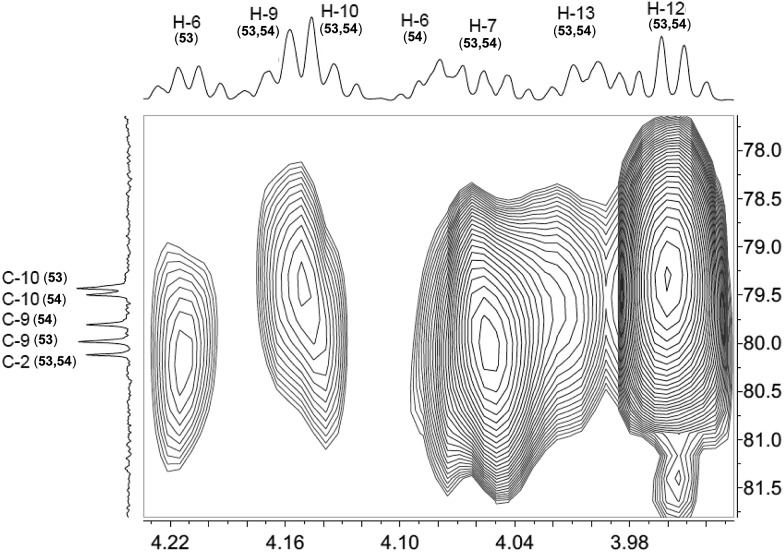
Expanded HMBC spectrum of a mixture of elatenyne (**53**) and laurendecumenyne B (**54**).

The splitting patterns of H-9 and H-10 in the ^1^H NMR spectrum of the mixture of elatenyne (**53**) and laurendecumenyne B (**54**) were originally reported as multiplets by Wang and co-workers,^161^ but when re-processing the FIDs, a distinct multiplicity was observed (Fig. 14). Even if the signals of H-9 and H-10 of **53** and **54** are completely overlapped, they should still feature the same doublet of triplets (dt) multiplicity, with coupling constants of ∼11.8 (t) and ∼5.9 (d) Hz. However, when the raw FIDs were processed with Reference Deconvolution and Lorentzian–Gaussian multiplication (LG) rather than the typical exponential multiplication (EM; Fig. 14) as window function, the multiplicities of the signal patterns were found to be more complex than one or two overlapping dt signals and appeared to be slightly asymmetric. After closer inspection, the resonances for H-9 and H-10 were recognized as being partially overlapped, resulting from A,B spin particles, and assigned to qdd (*J* = 6.8, 3.3, 1.4 Hz) and qd (*J* = 5.0, 2.8 Hz) splitting patterns, respectively. This interpretation was supported by the expanded HMBC correlations (Fig. 15). The overlap of the signals of H-9 and H-10 have also been observed by Kim and co-workers, but were assigned to identical chemical shifts by others.^160^,^161^,^165^–^167^ Notably, all the above splitting patterns exclude the structures of **51** and **52**, although it remains difficult to deduce the relative configuration between the two tetrahydrofuran rings unambiguously when relying on the re-processing and visual analysis of FIDs. Quantum mechanical full spin analysis (see Sections 3.3 and 5.1) will be required for unambiguous assignments. This also requires the availability of the raw data. On a more general note, the case of **53**/**54** provides another example, of why the ubiquitous use of the EM window function with LB = 0.3 is not a universally suitable post-acquisition processing method for ^1^H NMR spectra. The use of individually adjusted LG processing schemes typically yields additional structural information. This again speaks for the need to disseminate raw NMR data.

### 3.17 Second order coupling patterns with first order look *vs.* “multiplets”

Two prenylated chalcone antibiotics, 5′-*O*-methyl-3-hydroxyflemingin A (**57**) and 5′-*O*-methylflemingin C (**58**) occurring as enantiomeric mixtures, were isolated from the Sarawak rainforest plant, *Desmodium congestum*.^169^ The structures of **57** and **58** were determined using a combination of NMR (1D ^1^H/^13^C and appropriate 2D experiments) and HRMS.
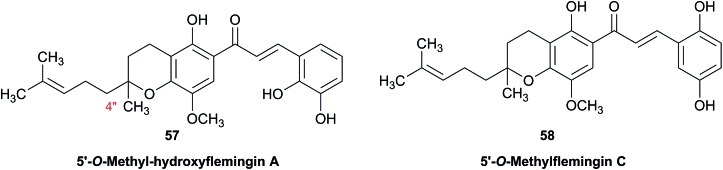


**Fig. 16:**
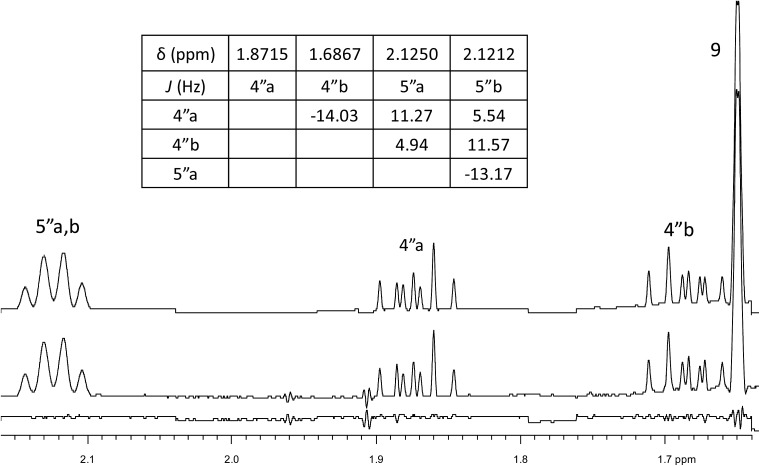
An expansion of the calculated (above) and experimental (middle) ^1^H NMR spectrum of 5′-*O*-methyl-3-hydroxyflemingin A (**57**), as well as the difference (residual; below); recorded in CDCl_3_ at 600 MHz. The table shows the relevant assignments, chemical shifts, and coupling constants.

During the ^1^H NMR assignment exercise, it was noted that the non-equivalent methylene hydrogens H-4″a + b displayed non-first order coupling patterns (Fig. 16). Although the chemical shift difference between H-4″a and H-4″b was only 0.18 ppm (∼108 Hz), one side of the multiplet for each methylene resonance “appeared” as a dd (*J* = 7.2, 10.2 Hz), while the other side “appeared” as a *t* (*J* = 8.7 Hz). This clearly indicated that the methylene signals for H-4″a and H-4″b exhibit 2^nd^ or higher order effects, and that the measured line distances (from the spectrum) are not reflective of the true *J* values. Given the relatively large difference between the methylene resonances, this second/higher order coupling pattern was unexpected and difficult to describe in terms of conventional NMR data table format. Designation of the signals as “multiplets” is common practice but not descriptive in the sense that it fails to provide any reproducible information. Retrieving raw NMR data from a repository allows for reprocessing and data analysis (spin simulation, full spin analysis) leading to a precise evaluation of *J* couplings in a second or higher order context (Fig. 16).

## 4 Impurity detection and quantification

**Fig. 17:**
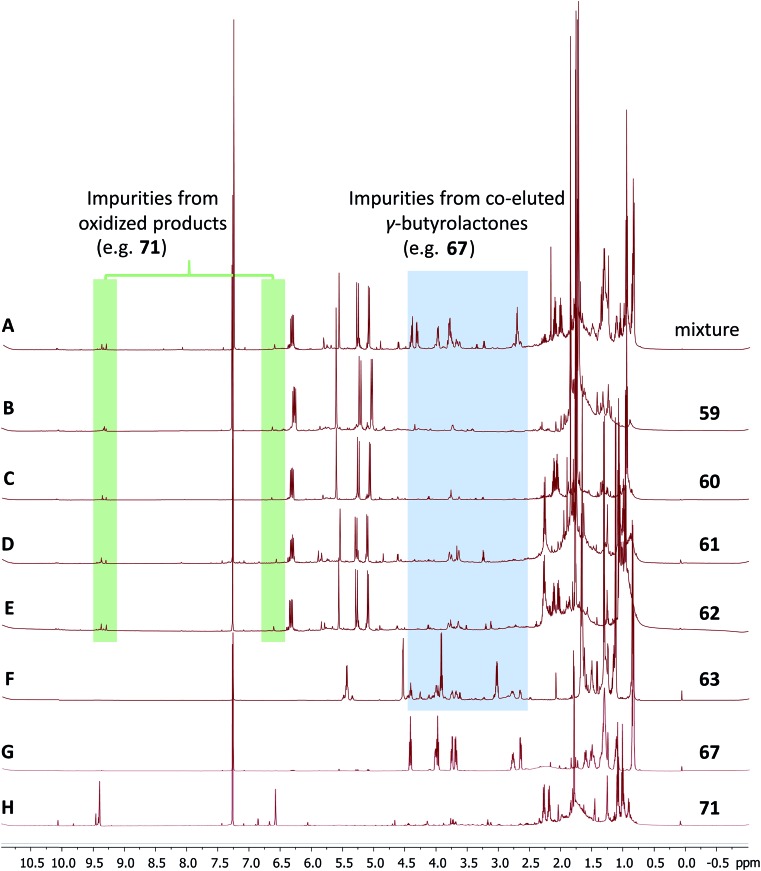
Comparison of the ^1^H NMR spectra of the target molecules to be isolated (**59–63**), the impurities contained, **67** and **71** and the mixture initially isolated (A).

Notwithstanding the above case of “multiplets”, and in addition to important but relatively straightforward structural revision, raw NMR data (FIDs) also plays an important role by enabling unambiguous reproducibility, as shown in the following cases (Fig. 17).

### 4.1 Purification of thiotetronates

A major advantage to accessing raw NMR data lies in the detection, identification, and quantitation of impurities. Impurities can be variable depending on the isolation procedures used as well as their physicochemical properties compared to the target molecules. Occasionally, certain impurity signals appear to be constantly observed in the NMR spectra of a class of compounds isolated. The impurities in these cases are often structurally and/or chemically closely related to the target molecules, likely derived from persistent co-elution or chemical transformation. Thus, having access to the raw NMR data, in combination with the increasing availability of advanced NMR processing and analysis software, can provide beneficial information about the amount and identity of this type of impurity. Such information can be used to optimize purification procedures and prevent chemical changes during the isolation of the target molecules or analogues. The impurities encountered during the isolation of thiotetronate antibiotics fall into this type, and reflect both scenarios for generating relevant impurities, *i.e.*, co-elution of structurally similar compounds hard to separate, as well as chemical changes of the target molecules during purification.

Thiotetrnate antibiotics are potent fatty acid synthase inhibitors bearing a thiolactone core structure. The isolation and structure identification of several thiotetronate antibiotics have been published.^170^,^171^ In comparison with the truncated ^1^H NMR spectra (0–9.0 ppm selected) in the ESI,† 170,171 shown in Fig. 17B–F are the full-scale ^1^H NMR spectra (–1.0 to 11.0 ppm) of five thiotetronates (**59–63**) regenerated from the raw NMR FID files. Similar impurity profiles are observed in the range 2.5–4.5 ppm of **59–63**.
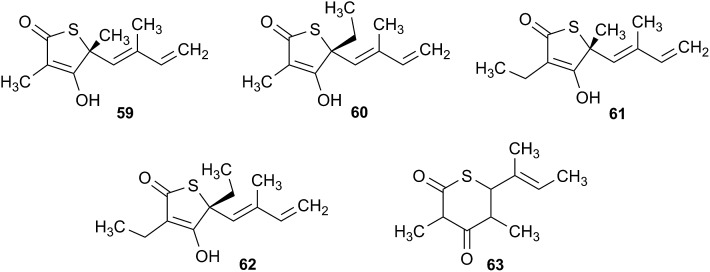


**Fig. 18:**
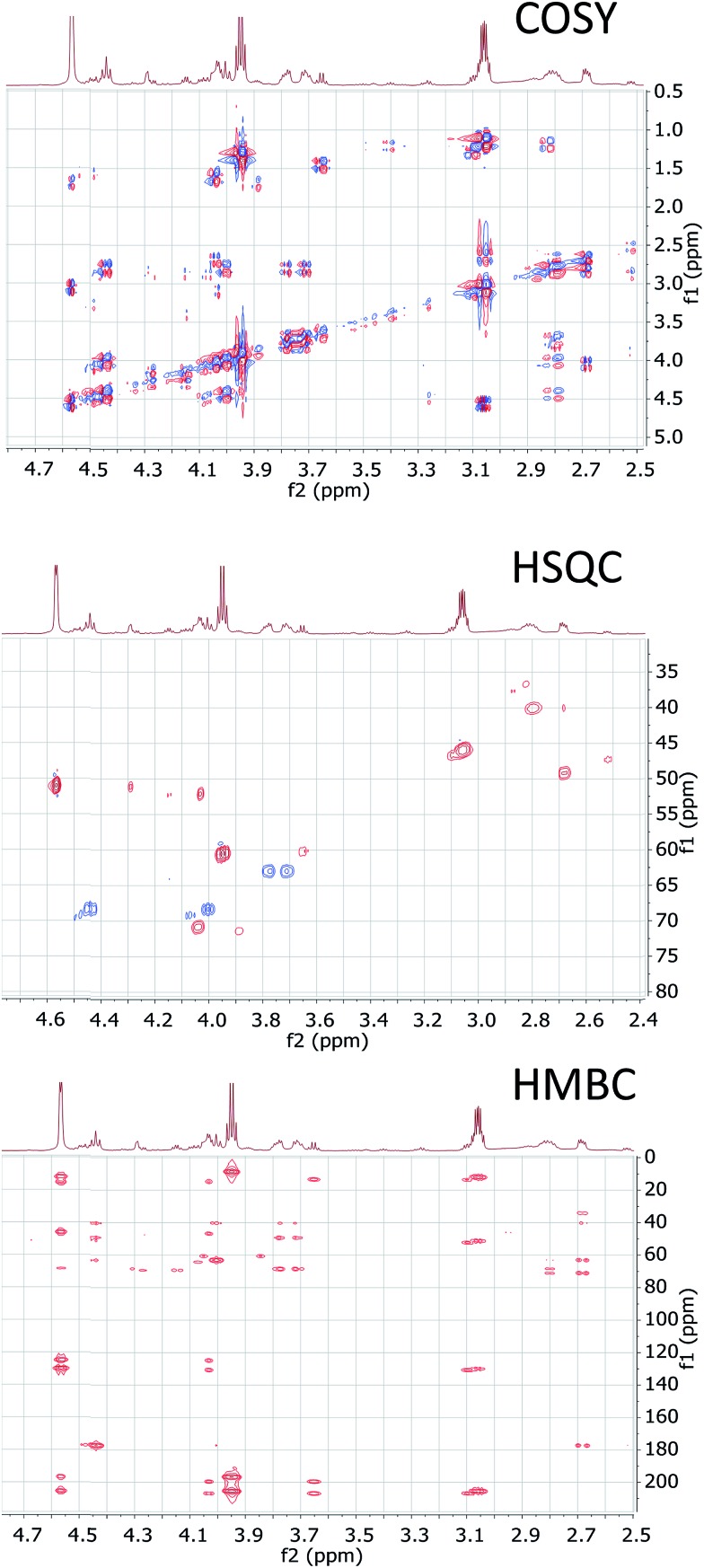
Expanded 2D NMR spectra of the thiotetronate (**63**) showing the focused region of the impurity.

Whereas the chemical shifts, integrations, and splitting patterns of these impurities are not readily recognizable in the original publications, the availability of the raw FIDs enabled a flexible, interactive, and facilitated analysis of the quantities and identities of these impurities. Taking the NMR spectra of **63** as an example: the expanded range of 2.5–4.5 ppm in the ^1^H NMR spectra (Fig. 17F) and analysis of the corresponding 2D NMR spectra (Fig. 18) pointed to the γ-butyrolactone class (*e.g.*, **64–67**) as the source of the impurity signals.
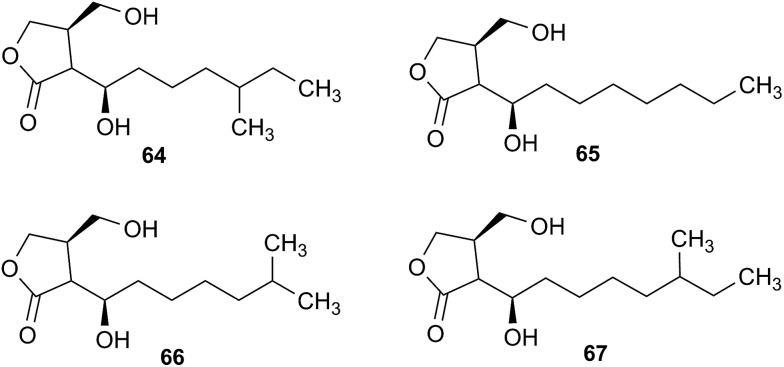


The γ-butyrolactones, common signaling molecules of the genus *Streptomyces*, share structural similarity with the thiotetronates (**59–63**) and lack obvious UV absorption. Thus, the isolation of a single symmetric signal from HPLC resulted in an initial ^1^H NMR spectrum that contained a mixture of compounds (Fig. 17A). Actually, a thorough analysis of the 1D and 2D NMR correlation map of this mixture led, not only to the identification of γ-butyrolactone as an impurity but also to the further optimization of isolation conditions. Through this optimization, the target molecules **59–63** in improved purity (Fig. 17B–F) were obtained, and a representative γ-butyrolactone **67** was also isolated for verification (Fig. 18G). This success, combined with the identification of γ-butyrolactone from the NMR spectra of **63** discussed above, exemplifies that some information about the impurities is often only accessible from the raw NMR FID files.

Furthermore, a characteristic aldehyde signal is observed around 9.5 ppm in the ^1^H NMR spectra of **59–62**, which was not included in the truncated spectra in the ESI† of the original publications.^170^,^171^ A thorough and complete analysis of the 1D and 2D NMR spectra of **59–62** containing this aldehyde impurity suggested **68–71** as candidate compounds responsible for this aldehyde signal and an associated singlet at 6.6 ppm. This hypothesis was confirmed by the isolation of a representative impurity **71** (Fig. 17H) from **62**.^172^
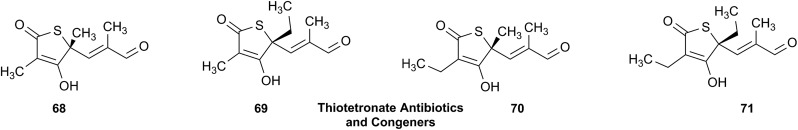


An association of the intensity of the aldehyde signal with the temperature and acidity used during the purification process suggested that they were likely the oxidation artifacts of the corresponding thiotetronates (**59–62**). Despite the unclear mechanism underlying this process, the interpretation of the aldehyde-containing impurity provided additional information about the chemical stability of the target molecules and helped to optimize the isolation procedure at the early stage of this study. Thus, access to the raw FIDs of these molecules might likewise enable others to gain more information for developing suitable purification procedures.

To sum up, the availability of raw NMR FIDs not only accurately indicates the purity of the target molecules isolated, but also provides otherwise inaccessible information about the identity of relevant impurities co-eluted with, or chemically transformed from, the target molecules.

### 4.2 Dynamic equilibria between isomers

Another example dates back more than 20 years to work on sesquiterpenoid lactones (STLs) in North American Star Anise (*Illicum*) species. From leaves and fruits of *I. floridanum* (Florida Star Anise), a variety of lactones of the *seco*-prezizaane (= *seco*-allo-cedrane) type were isolated.^173^–^176^ In addition to several new related STLs, the known pseudoanisatin (**72a**) was found, representing one of the major constituents in both the leaves and fruits. Its structure elucidation was based on NMR, whereas, the X-ray crystallographic data had been published previously by Kouno *et al.*^177^,^178^ The NMR data (pyridine-*d*_5_) of the isolated constituent were identical with those published, but the isolates (irrespective of the plant part from which they came) always contained some 10% of an impurity whose signals clearly indicated a structural relationship with the main component. However, it was not possible to solve the structure of the minor component on the basis of the available spectra. A number of attempts were made to further purify **72a**, especially since bioassays were planned. However, none of these attempts led to a diminution of the impurity but only to a loss of yield.

Later, the study on North American *Illicium* species was extended to the leaves of *I. parviflorum* from which a new lactone with an unusual and unprecedented cyclic hemiketal structure containing an oxygen bridge between C-4 and C-7 was isolated and named cycloparviflorolide **73b**.^175^ This compound was found to contain some 20% of an isomeric compound which could be identified as **73a** (parviflorolide) lacking the hemiketal ring and bearing the oxo and hydroxyl functions at C-7 and C-4 respectively, thus representing a direct analogue of **72a**. It became clear that the compound actually exists as an equilibrium mixture between the two forms, which are hence also inseparable from each other.
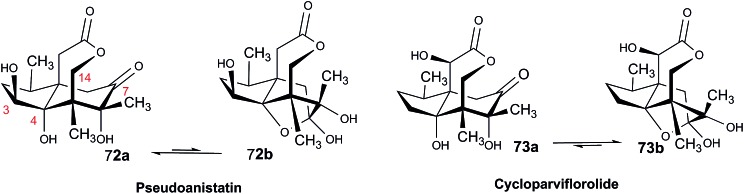


**Fig. 19:**
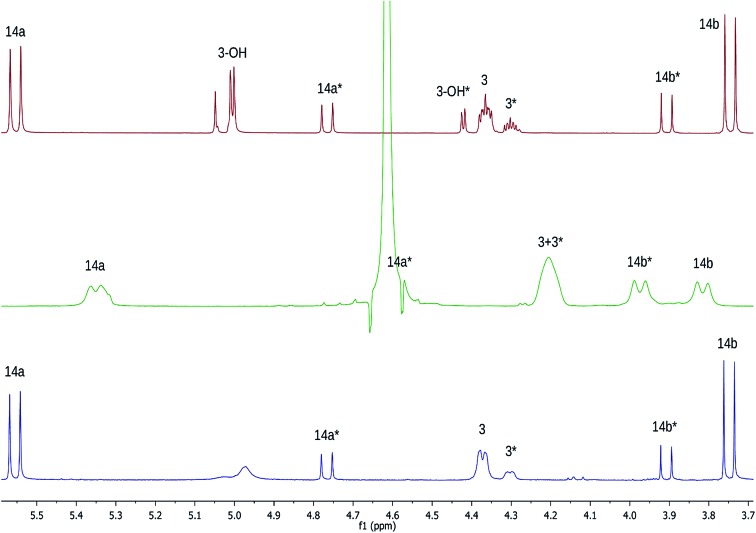
Low field region of the ^1^H NMR spectrum (500 MHz) of pseudoanisatin (**72a + 72b**). Top: spectrum in acetone-*d*_6_. Middle: spectrum of the same sample in D_2_O; bottom: spectrum of the same sample re-dissolved in acetone-*d*_6_ after the measurement in water. All assignments confirmed by 2D spectra. Signals of the cyclic hemiketal form **72b** are marked with an asterisk. It becomes obvious from the signals of H-14b that water stabilizes the latter. Full reversibility of the change in the equilibrium is demonstrated by the spectrum shown at the bottom. The change of multiplicity of the H-3 signals is due to H/D exchange.

Given the almost identical structures of **73a** and **72a** it was straightforward to expect that this type of equilibrium would exist also in the case of pseudoanisatin **72a** and a cyclic form **72b**, which should then represent the 10% “impurity”. Re-analyzing the NMR spectra of pseudoanisatin showed that the signals of the minor constituent indeed correspond to the cyclic hemiketal form, *i.e.*, cyclopseudoanisatin **72b**, and that this is actually the reason for the inseparable “impurity”. While in case of **73a**/**b**, the 4,7-cyclo-form is the major isomer (80%, spectra recorded in acetone-*d*_6_), in case of **72a**/**b** the 7-oxo-form was found to be predominant (with a ratio of approximately 80 : 20 in this solvent (Fig. 19)). It was subsequently demonstrated, based on theoretical considerations, that the respective oxo-isomers of both compounds are very likely the bioactive forms responsible for the binding to insect GABA_A_ receptors.^179^

Had the original spectra (a good copy of the 1D ^1^H and ^13^C NMR spectra would certainly have been sufficient) of pseudoanisatin been available, it would have been clear from the beginning, that the “impurity” must also have been present in the previous authors' isolate obtained from a different species, *I. anisatum*. This could have given a hint that it was not just some other STL present in minor amount but that it actually represents another form of the pseudoanisatin molecule. Much futile purification work could probably have been saved. It is simply not possible to obtain NMR spectra of more than 90% “pure” pseudoanisatin in the solvents used (pyridine-*d*_5_, acetone-*d*_6_, D_2_O) due to this equilibrium in solution. In fact it was shown later that the equilibrium composition in both cases is dependent on the solvent. It was found that water stabilizes the cyclic hemiketal isomers and shifts the equilibrium composition in this direction, leading to an approximately 1 : 1 mixture in the case of **72a** and **72b** (Fig. 19).

### 4.3 Detection of rotamers

**Fig. 20:**
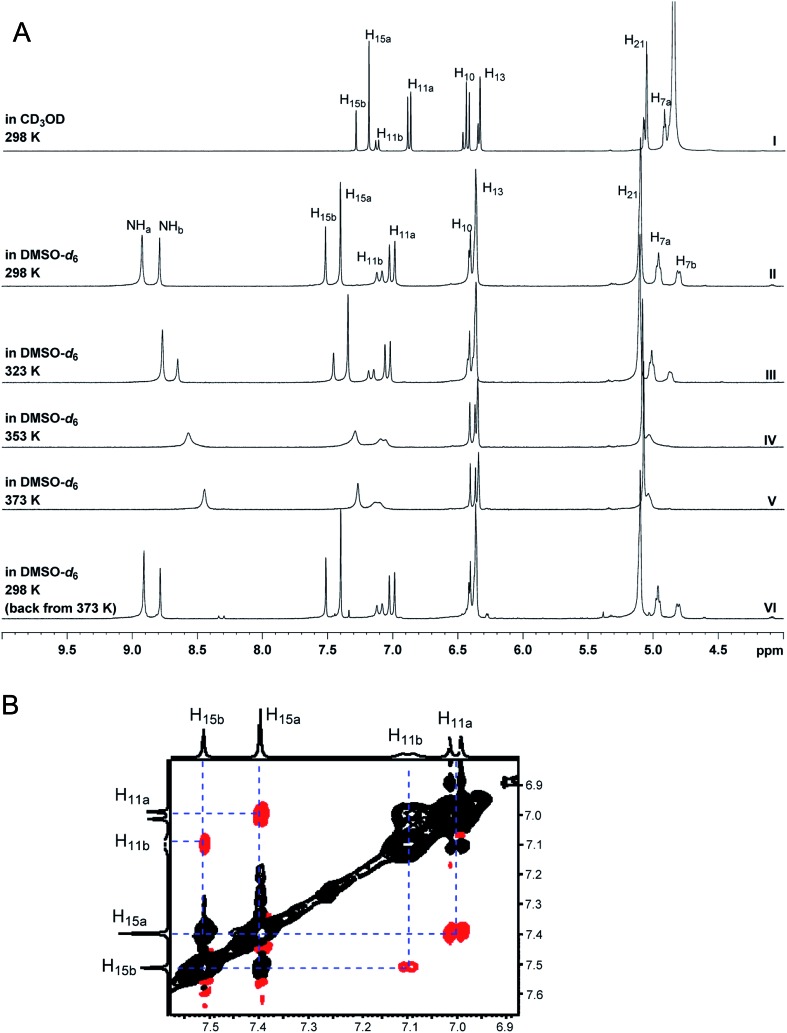
NMR techniques that facilitate the identification of natural products existing as rotamers as exemplified by the structural elucidation of guangnanmycin A (**74a**/**b**). (A) ^1^H NMR spectra of guangnanmycin A recorded in CD_3_OD (I) and DMSO-*d*_6_ at varying temperatures (II–VI). (B) ROESY spectrum of guangnanmycin A with red signals denoting normal NOE correlations and black signals denoting the exchange correlation signals between the two rotamers appearing in the opposite phase.

Guangnanmycin A (**74a**/**b**), a new member of the leinamycin family of NPs, was isolated from *Streptomyces* sp. CB01883 by the Shen group's efforts to target Nature's combinatorial biosynthetic potential for the discovery of novel NPs.^180^ Unlike the other members of this family, as exemplified by leinamycin^181^ or leinamycin E1 182 that displayed a single set of signals upon NMR analysis, **74a**/**b** afforded two sets of signals, in a ratio of ∼2 : 1, in its ^1^H NMR spectrum recorded in DMSO-*d*_6_ at 298 K (Fig. 20, panel A-II). Initially, it was not apparent if the complication of the spectrum resulted from the presence of impurities or two equilibrating rotamers, **74a** and **74b**.
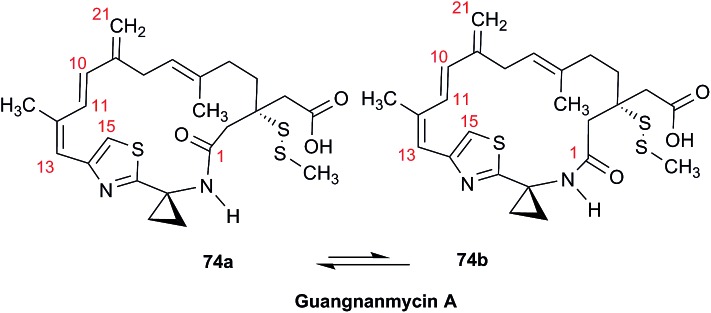


We thus analyzed the ^1^H NMR spectrum of guangnanmycin A in CD_3_OD at 298 K (Fig. 20, panel A-I), revealing that the ratio of the two sets of signals changed to ∼3 : 1, hence suggesting the presence of two rotamers rather than impurities. Other NMR technologies were employed to support the attribution of the two sets of signals to the presence of two rotamers of guangnanmycin A (Fig. 20), as exemplified by the variable-temperature NMR experiment, in which the signals of two rotamers tend to merge at elevated temperature and finally fuse to one set at 393 K (Fig. 20, panel A II–VI), and the ROESY experiment, in which the exchange cross-signals between the resonances of rotameric forms, *e.g.*, H-11 (at 7.00 and 7.10 ppm) or H-15 (at 7.40 and 7.51 ppm), appear in the opposite phase (shown in black), to that of normal NOE correlations between H-11 (at 7.00 and 7.10 ppm) and H-15 (at 7.40 and 7.51 ppm) (shown in red) (Fig. 20 panel B). While the varying NMR experiments afford ultimate confidence to the final structural assignments, analyzing the raw data of ^1^H NMR obtained in different solvents at ambient temperature requires less time, thereby highlighting its simplicity and usefulness in structure elucidation of NPs that occur as rotamers.

## 5 Dereplication

### 5.1 Structural dereplication of proanthocyanidin A1 with higher order spin systems

Comparison of basic ^1^H NMR parameters (chemical shifts, coupling constants, line widths, and signal integrals) of isolated compounds with those of already reported structures is a standard method for a rapid structural dereplication. Proanthocyanidin A1 (PCA1, **75**), is one of the most common dimeric proanthocyanidins which has one A-type doubly linked interflavanyl bond (C–C and C–O–C). In a previous study, PCA1 was isolated from the bark of *Pinus massoniana*, and the structure was identified by interpretation of 1D and 2D NMR spectroscopic data in combination with an electronic circular dichroism (ECD) experiment.^183^,^184^ The initial attempt at structural dereplication by comparison of reported ^1^H NMR data failed due to inconsistency of the reported data caused by difficulties of interpretation of higher order spin systems as shown in Table 3. In the literature, chemical shifts, coupling constants, and multiplicities continue to be described using the tabulated (depicted) method, and this can cause confusion. Two strongly coupled hydrogens (E-H-5′ and E-H-6′; split into d and dd, respectively, following first order analysis) were described inconsistently in summaries. Even if NMR data are collected in the same solvent and at the same temperature, the errors between experimental and reported data, and between the references are outside the acceptable range to be considered as the same compound. Subtle differences considered to be negligible could be from near identical but different structures.^60^,^183^–^185^ 
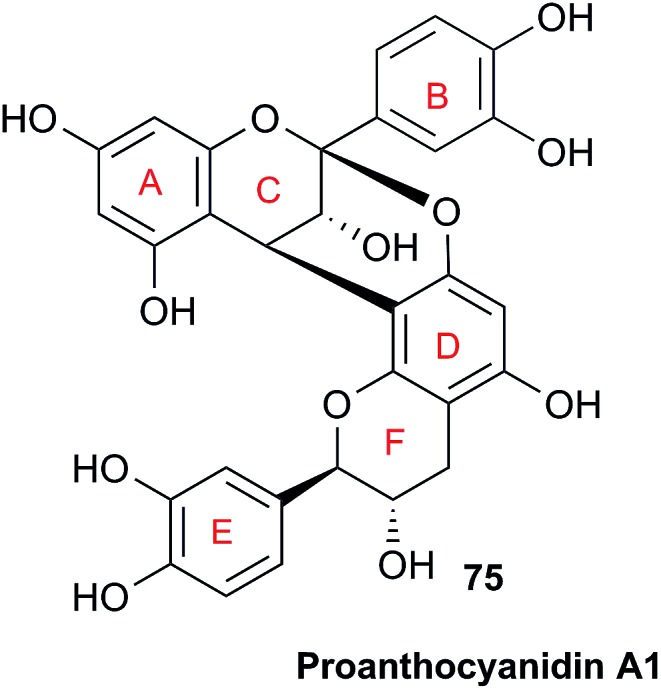


**Table 3:** Comparison of ^1^H NMR data of PCA1 (**75**) in the literature^*a*^

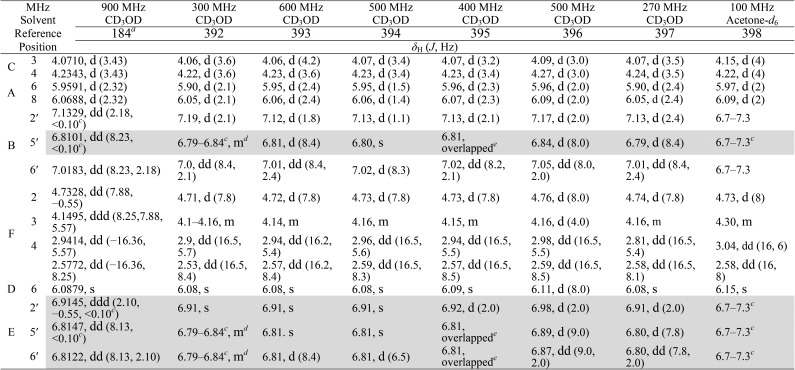

^*a*^The *δ*_H_ (ppm) and *J* (Hz) values were determined by ^1^H iterative full spin analysis (HiFSA).

^*b*^Very small couplings were detected by HiFSA and required for the overall fit. Depictions in grey colored box are inconstancy due to difficulties of interpretation of higher order spin systems, E-H-5′ and E-H-6'. Signals in this region were described as ^*c*^range, ^*d*^multiplet, and ^*e*^overlapped. This can lead to ambiguity.

**Fig. 21:**
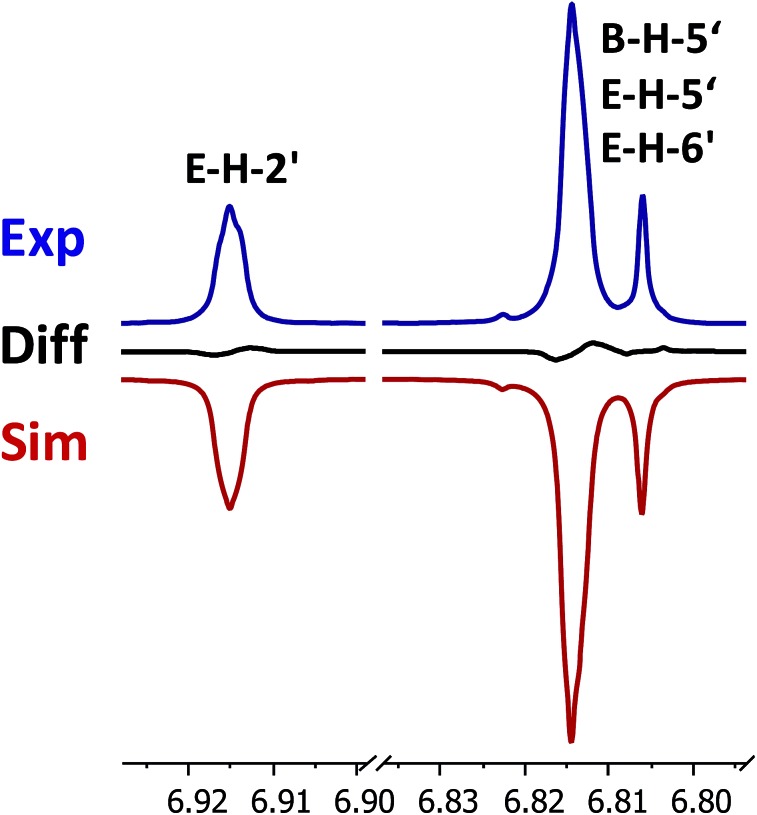
Case study of proanthocyanidin A1 (PCA1, **75**) which shows higher order effects. Quantum mechanical simulation (HiFSA) allows producing accurate NMR parameters of the experimental spectrum (Exp, in blue) and a perfectly fitted simulated spectrum (Sim, in red).

**Fig. 22:**
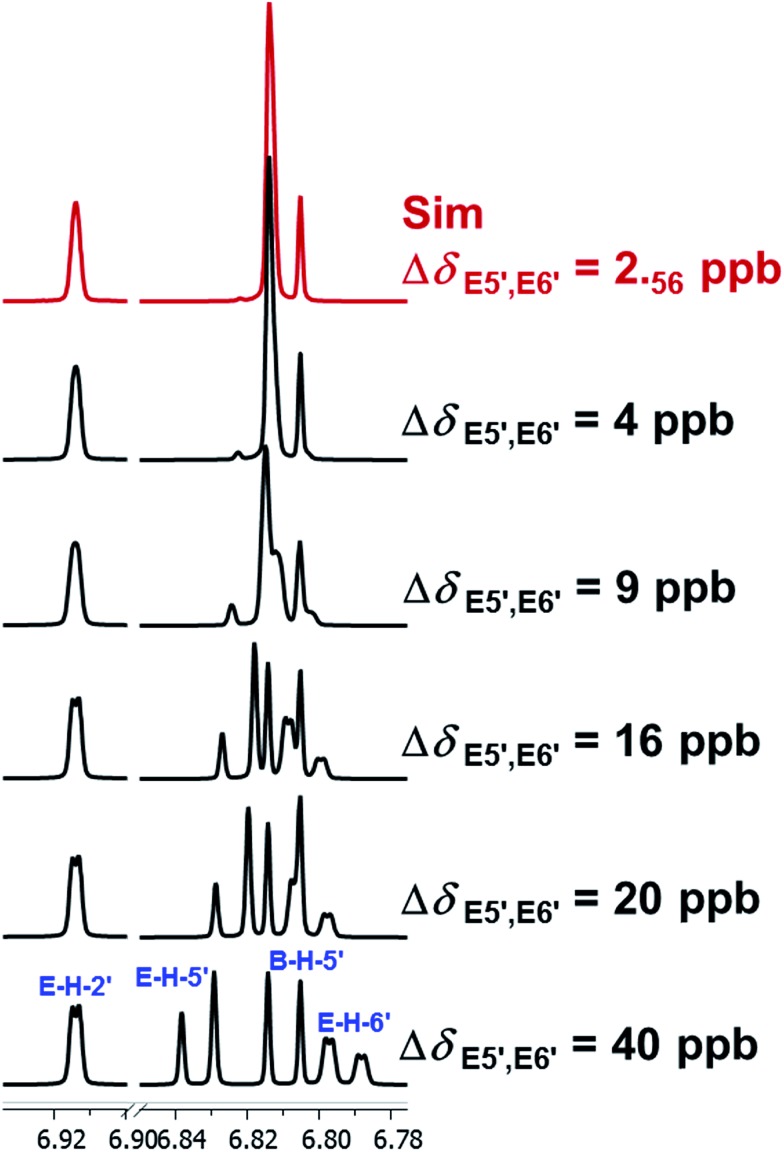
Simulation of higher order spin systems of E-H-5′ and E-H-6′ in proanthocyanidin A1 (PCA1, **75**) with various distances between two coupled-hydrogens. Simulation was performed with the PERCH software tool.

These subtle differences can be easily overlooked when the chemical shifts and coupling constants are calculated by a conventional manual measurement. In order to reduce the errors, HiFSA (^1^H iterative Full Spin Analysis) was applied to calculate the spectral parameters with high precision (*δ*_H_, 0.1 ppb; *J*, 10 mHz).^60^ HiFSA from the FID data can produce accurate NMR parameters (chemical shifts, coupling constants) for even in higher order spin systems (Table 3, Fig. 21). Fig. 22 illustrates the higher order effects as a function of the various distances between the coupled hydrogens (E-H-5′ and E-H-6′), which shows the disappearance of d and dd multiplicities upon decreasing Δ*δ* between these two hydrogens. This case study clearly emphasizes the fact that tabulated summaries can lead to repetitive spectral misinterpretation; therefore, it is necessary to provide access to raw FID data for rapid and accurate structural dereplication of previously identified compounds.

### 5.2 HSQC as a dereplication tool

A critical aspect of modern NP research is the rapid, efficient and accurate dereplication of known compounds.^186^ With some 50 000 NPs reported in AntiMarin^187^ and 139 000 in the Dictionary of Natural Products,^188^ there exists a significant likelihood that a newly isolated substance may be identical or related to a known NP. Unless re-isolation or verification is the ultimate goal, it is typically a poor utilization of laboratory and human resources to spend significant amounts of time in the isolation and complete structure determination of an NP only to find that it was previously reported. A caveat to this, however, is that isolation of a compound of novel biology can be a significant scientific contribution even if the structure is known; nevertheless, one wants to establish this as quickly as possible. A variety of techniques have been utilized effectively for this, including biological assay profiles, variations of LC-DAD-MS analysis,^189^,^190^ and NMR metabolomics.^191^ It was with this goal in mind that a method to categorize the similarities in NMR spectra between different NPs as sought, as an additional basis of automatic dereplication of known NPs and their analogues.

This contribution focuses on the ^1^H–^13^C HSQC spectrum as the critical NMR data set as the most robust yet cleanly characteristic of a given molecule, in part because of the high resolution created in the 2D NMR data set between all hydrogenated carbons and their respective hydrogens, and in part because there are fast NMR methods, such as Non-Uniform Sampling (NUS),^192^ ultrafast NMR,^193^ and Ernst angle-based signal intensity optimization methods^194^ for acquiring full 2D data sets. Further, the use of a deep Convolution Neural Network (CNN) with a Siamese architecture has a more robust ability to learn the features of different classes of images even when there are only a few images per image class, as well as to recognize patterns or objects in images even in the presence of artifacts (Fig. 22).^195^

However, to provide the deep CNN with an adequate training set, required the accumulation of a few thousand of such ^1^H–^13^C HSQC spectra, which were found in the ESI† pages of the Journal of Natural Products. While the spectra are there, they are present in many different formats, with grid lines or without, with assignment annotations, and presence of signal color for phase-edited HSQC experiments. In order to use these to teach a deep CNN, they needed to be extensively cleaned of this extraneous content. Whereas this could be achieved using post-processing image modifying software such as GIMP (GNU Image Manipulation Program; ; gimp.org), it would have been highly desirable to have direct access to the raw untransformed data, in which case it would have been possible to optimize transformation and plotting parameters to produce standardized image files of the highest comparability (*i.e.*, neat 2D HSQC spectra with a fixed scale in each dimension).

**Fig. 23:**
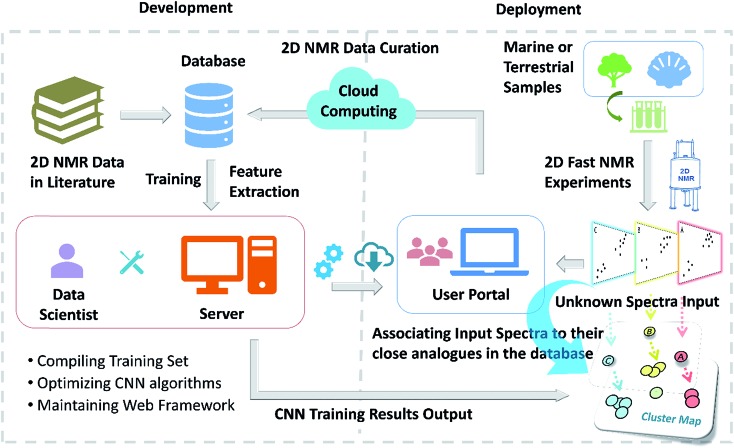
Workflow for the Web-Based Small Molecule Accurate Recognition Technology (SMART). The workflow is divided into two parts; ‘development’ and ‘deployment’. In the development section, new HSQC inputs are curated by SMART and used to train the modified deep Convolutional Neural Networks (CNN) algorithm. The training process is performed using cloud computing or a server machine. The training data set is compiled, the CNN algorithm tuned, and the web framework maintained. The training data set is compiled by merging user uploaded HSQC spectra and HSQC spectra obtained from literature publications. In the deployment section, HSQC spectra of newly isolated pure natural product molecules are automatically embedded by SMART into a cluster space near similar, previously-characterized compounds in the training data set. The resultant embedding in the cluster map is visualized in a 2D cluster map (nodes: HSQC spectra processed by SMART; node colors: compounds from the same natural product family; internode distance: a quantification of molecular structural similarity).

Nevertheless, a modified deep CNN, was populated and designated the Small Molecule Accurate Recognition Technology (SMART) platform, with these refined HSQC spectra, and then this trained system was utilized to analyze new spectra and place them in a location within the SMART map that assists in their structure identification.^196^ To demonstrate and authenticate SMART (Fig. 23), a series of molecules isolated from two different marine cyanobacteria, a *Rivularia* sp. from Vieques, Puerto Rico, and a *Moorea producens* from American Samoa, were analyzed by NMR and their HSQC spectra rapidly recorded using NUS pulse sequences. When queried by SMART, these were placed in close proximity to a couple of series of related cyanobacterial cyclic lipopeptides, namely the viequeamides^197^ and veraguamides. Ultimately, the compounds were fully characterized by a variety of spectroscopic methods, and their structures shown to be closely related to the viequeamides (Fig. 23).^198^

### 5.3 Dereplication during fractionation

The NMR spectrum of a fraction is a fingerprint of its entire chemical composition and, therefore, never lies about the composition of fractions. While ^1^H-NMR has been frequently used in metabolite fingerprinting of NPs, the advent of high field instruments together with cryoprobes and small volume tubes (3 or 1.7 mm NMR tubes) have addressed the previous limitation of low sensitivity so that NMR spectra of fractions can be directly analyzed to identify constituents. The deposition of raw data and the associated FID data will allow complete analysis of fractions. The advantages can be two-fold: to easily and quickly identify known compounds within fractions without further isolation, and to identify signals that are not within known compounds in the search for novel molecules.

The aim of this example was to develop NMR fingerprints to identify novel compounds by first demonstrating the value of NMR fingerprints of fractions to identify novel compounds from a set of 20 sponges from the order Poecilosclerida. The presence of a unique ^1^H NMR spectral pattern in only 5 of the 220 spectra allowed the isolation of the novel compound iotrochotazine A (**76**) that was shown to have phenotypic activity on cells from Parkinson's Disease patients.^199^

The NMR of an active fraction with LAT3 inhibition ensured that the four compounds in the fraction were isolated. In this case, LC-UV-MS proved to be of limited value as the compounds had little UV absorbance and the ESI mass spectrum contained mainly fragment ions. The ^1^H NMR spectrum, on the other hand, revealed the presence of multiple compounds, providing a comprehensive fingerprint of all of the small molecules contained in the fractions. This resulted in the isolation of four novel compounds, venulosides A–D (**77–80**), whose structural relatedness had the advantage of providing SAR information.^200^
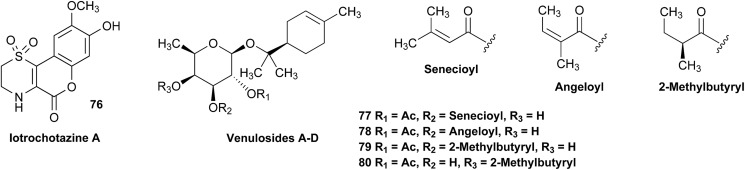


The metabolome of a termite-gut associated actinomycete using NMR fingerprints identified six new NPs, namely, the actinoglycosidines A and B (**81** and **82**), actinopolymorphol D (**83**), and the niveamycins A, B, and C (**84–86**).^201^ The metabolic fingerprinting approach in this publication reports the methodology. It consisted of the generation, through RP-HPLC, of five LLE fractions for each of the eighty-four crude extracts (21 strains/four crude extracts: OMA, LFA, RFA, and GYES) using parameters such as log *P* < 5 that permitted the retention of molecules with lead and drug-like properties.^202^,^203^ 
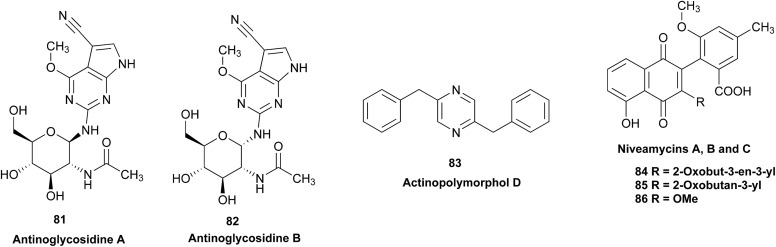


**Fig. 24:**
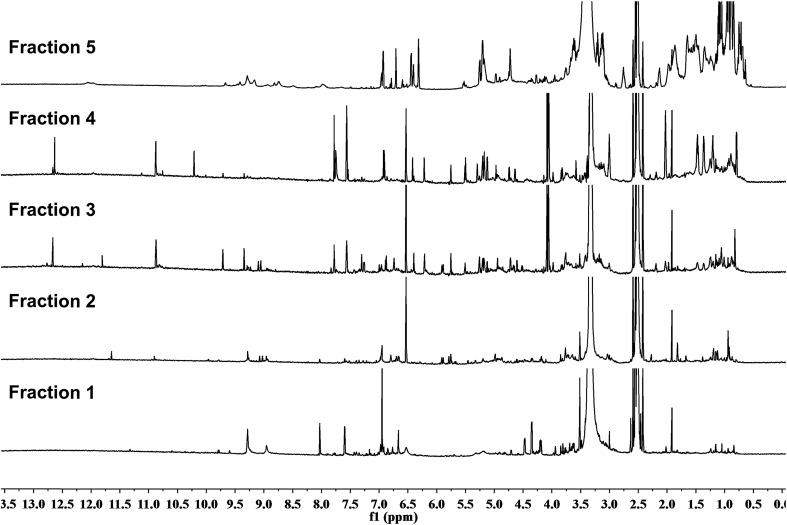
NMR spectra of five lead-like enhanced (LLE) fractions of the extract *Sauropus* sp. The fraction samples were prepared from NatureBank at the Griffith Institute for Drug Discovery (; https://www2.griffith.edu.au/institute-drug-discovery).

**Fig. 25:**
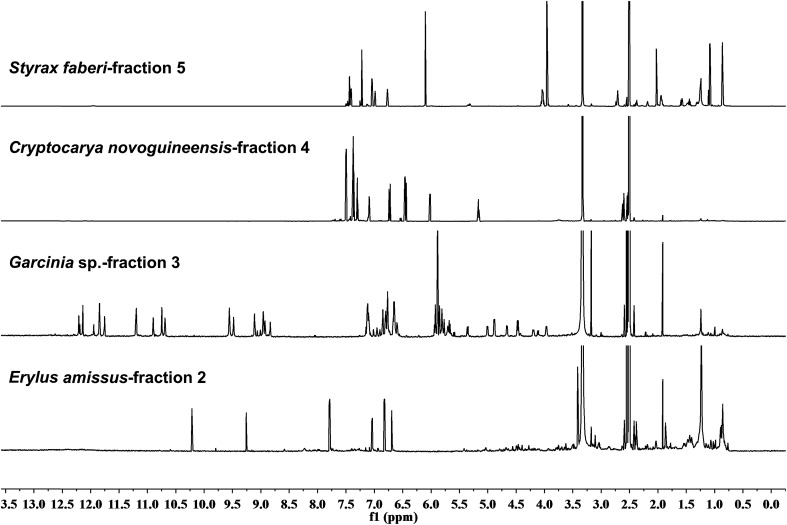
NMR fingerprints of single active fractions from four taxa, *Erylus amissus*, *Garcinia* sp., *Cryptocarya novoguineensis*, and *Styrax faberi*. The fraction samples were prepared from NatureBank at the Griffith Institute for Drug Discovery (; https://www2.griffith.edu.au/institute-drug-discovery).

NMR fingerprints allowed suppression of metabolites, induction of new metabolites, and increased production of minor compounds to be determined after treatment with *N*-acetyl-d-glucosamine in three sponge-derived actinomycetes.^204^ These examples demonstrate the need to establish a ^1^H NMR NPs database of raw data that can be freely accessible in order to focus on novel NPs. Moreover, they exemplify the need for NMR raw data to allow NMR fingerprints to become a universal tool. Typical NMR fingerprints of fractions are shown in Fig. 24 and 25, and can be analyzed using the proposed database of raw files.

### 5.4 The configuration of lanciferine

The monoterpene indole alkaloid (MIA), lanciferine (**87a**), was isolated in 1973 from the aerial parts of the New-Caledonian plant, *Alstonia boulindaensis* Boiteau (Apocynaceae)^205^ and belongs to the akuammiline family.^206^ Engendering numerous complex scaffolds, the akuammiline MIAs have received much attention by synthetic chemists owing to their molecular structures and a broad range of biological activities.^207^ The oxidized furoindoline motif in **87a** is embedded within a polycyclic framework, referred to as “indolinolid” in the original report.^205^ Although the molecular framework of **87a** was the first of its kind, the akuammiline MIAs have since been expanded by nine congeners: picranitine,^208^ alstolactines A, B, and C,^209^ alstoniascholarines L and M,^210^ as well as scholarisines K, L, and M.^211^

Research concerning the akuammilines has focused on isolation and pharmacological studies^212^ with relatively less emphasis on synthetic chemistry. However, synthetic endeavors spanning the past 30 years have resulted in the design of elegant and successful total syntheses. The asymmetric total syntheses of the three akuammiline alkaloids, aspidodasycarpine, lonicerine and the proposed structure of lanciferine (**87a**), was completed recently by Li *et al.*^213^ According to the authors, the structural reassignment of their product was hampered by the ambiguous and incomplete ^1^H NMR data disclosed in the isolation report. In addition, the ^13^C NMR data were also missing (in the mid 1970's, ^13^C NMR analysis was still very much a specialist's technique and widely inaccessible to NP research groups). However, a thorough analysis of just the ^1^H NMR spectrum, enabled by the availability of the raw data, would have revealed any inconsistencies with Ang Li *et al.*'s interpretation. Indeed, the ^1^H NMR chemical shift of C18 methyl of the synthesized compound (**87b**, 19*S*) (1.4 ppm) differed from that reported for natural lanciferine **87a** (1.2 ppm). Furthermore, for the original isolation of **87a**, the authors reported the unambiguous assignment of the configurations of all its chiral centers except that of C-19.^214^
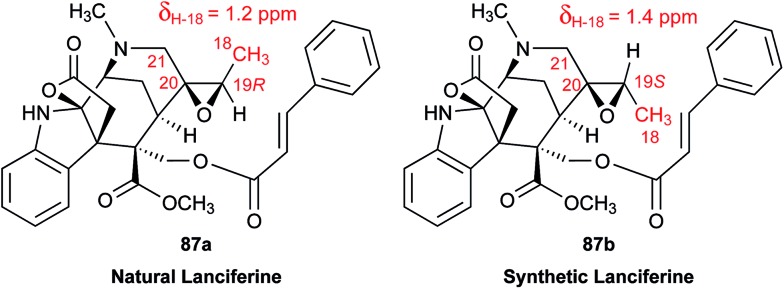


In light of these data, it would seem that Ang Li *et al.*, actually, did not synthesize **87a** but a diastereoisomer, **87b**. Continuing interest of Beniddir's group in MIA chemistry led to the development of a spectral database of a cumulative collection of alkaloids, for dereplication purposes.^215^ Hence, it was possible to retrieve the original sample of **87a** and reacquire reliable 1D and 2D NMR spectra. These data in conjunction with a detailed NMR-based computational study using the CP3 parameter^209^ shed light on the configurational assignment of lanciferine and confirmed the 19*R* and 19*S* configurations for **87a** and **87b**, respectively.^216^

In conclusion, this case of ambiguity would have been removed if the raw data (*i.e.*, FID) of the NMR of **87a** had been made accessible.^1^ Indeed, FIDs or spectra availability, would have enabled the structure verification of **87a** through computer-assisted spectral assignment approaches.^15^ Finally, this example brings out the need for new reporting standards for NMR data and more globally, NPs' spectral properties.

### 5.5 Unraveling the *J* values of mycothiazole

Mycothiazole (MYC, **88**)^217^ is a bioactive sponge-derived polyketide-nonribosomal peptide synthetase (PKS/NRPS) hybrid product of continuing interest as a lead for an anti-cancer therapeutic.^218^,^219^ 
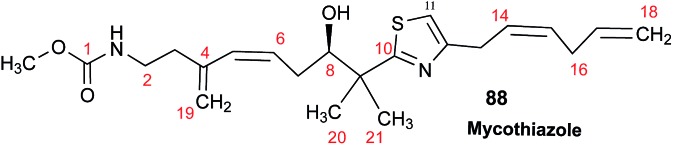


**Fig. 26:**
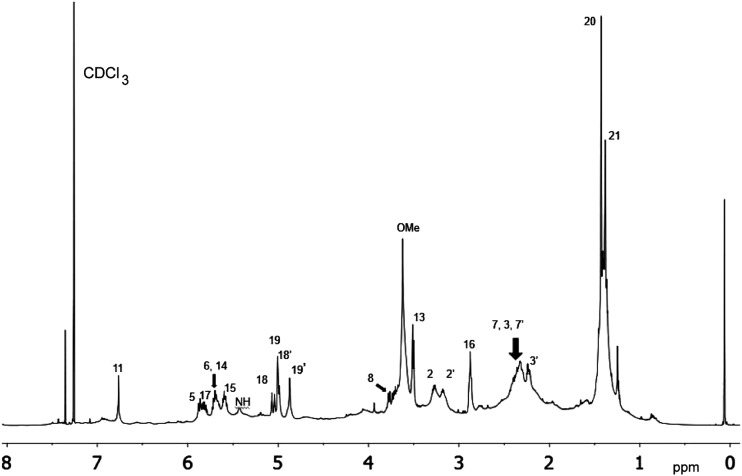
Mycothiazole (**88**) full ^1^H NMR spectra (CDCl_3_, 600 MHz) annotated with atom position numbers with output obtained by classical FID work-up.

The current understanding of the exact pharmacophore needed for its nM profile in cytotoxicity screening is incomplete and is the subject of continuing study of analogs. MYC ^1^H and ^13^C NMR data acquired at 300 MHz in CDCl_3_ were misinterpreted. A subsequent re-evaluation took place prompted by discrepancies in the ^13^C shifts and optical rotation data between natural and synthetic products.^220^,^221^ Further evaluation involved data collected at 600 MHz.^220^ Shown in Fig. 26 is that several resonances are broadened and overlapping. This confounds the task of extracting many *J* values, so many signals were listed as “m” in the original publication.^217^ The second generation analysis at 600 MHz^220^ included obtaining NOE data and remeasuring the *J* values for H-15 (5.62 ppm) as a dtt (*J* = 10.7, 7.5, 1.5 Hz) prompting the reassignment of the C-14, C-15 geometry from *E* to *Z* (Fig. 26).

**Fig. 27:**
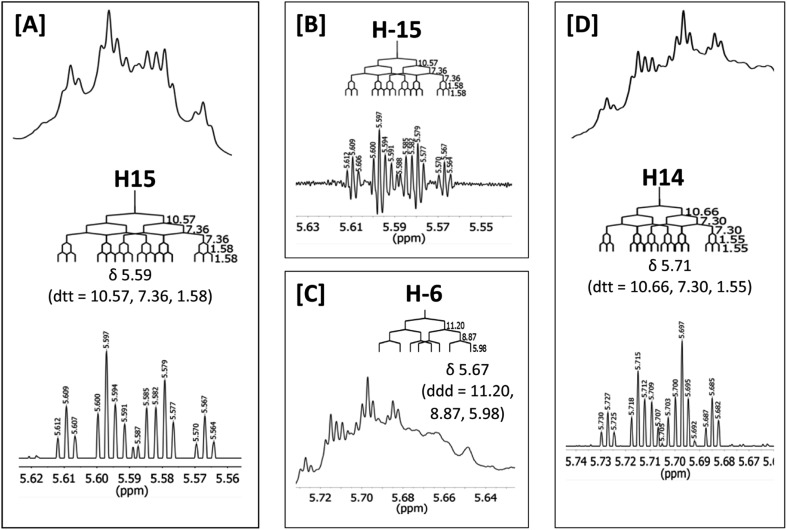
Mycothiazole (**88**) expanded ^1^H NMR spectra regions (CDCl_3_, 600 MHz) obtained from different FID processing. [A] H-15: top panel – classic FID workup, middle panel – *J* (Hz) measurements, bottom panel – FID workup using second derivative/nonlinear fitting processing. [B] H-15: top panel – *J* (Hz) measurements, bottom panel – FID reprocessing using a sign square apodization *vs.* that used for [A] bottom panel. [C] H-6: top panel – *J* (Hz) measurements, bottom panel – classic FID workup. [D] H-14: top panel – classic FID workup, middle panel – *J* (Hz) measurements, bottom panel – FID workup using second derivative/nonlinear fitting processing and suppression of H-6 resonance signals.

New FIDs have been obtained for MYC and are available as electronic information. Presented below are examples for which obtaining new FIDs enable accurate measurement of *J*_HH_ and *J*_HC_ values for the first order or non-first order multiplets. The first example involves closely overlapping resonances of olefinic hydrogens H-6, H-14 and H-15. Shown in Fig. 27 is a before-and-after data set with the new data provided by the two methods of post-acquisition processing. This allowed the accurate measurement of nine *J* values as shown in each of the panels. The principal tool used here was the second derivative/nonlinear fitting algorithm “Resolution Booster” developed by Mestrelab Research SL to reprocess the 1D NMR FID. Using this algorithm along with the post-acquisition Resolution Booster option, it was possible to clearly resolve all 16 multiplet lines of H-15 with surprising improvement of resolution without introducing artifacts or shifts in the spectrum. This enabled confident multiplet assignment along with accurate measurement of ^3^*J*_H-15–H-14_ and ^3^*J*_H-15–H-16_ data shown (Fig. 27) that differed from those reported in 2006 (see above). The data in Fig. 27C and D provide additional coupling values for H-14 and H-16 previously described simply as multiplets.^217^

**Fig. 28:**
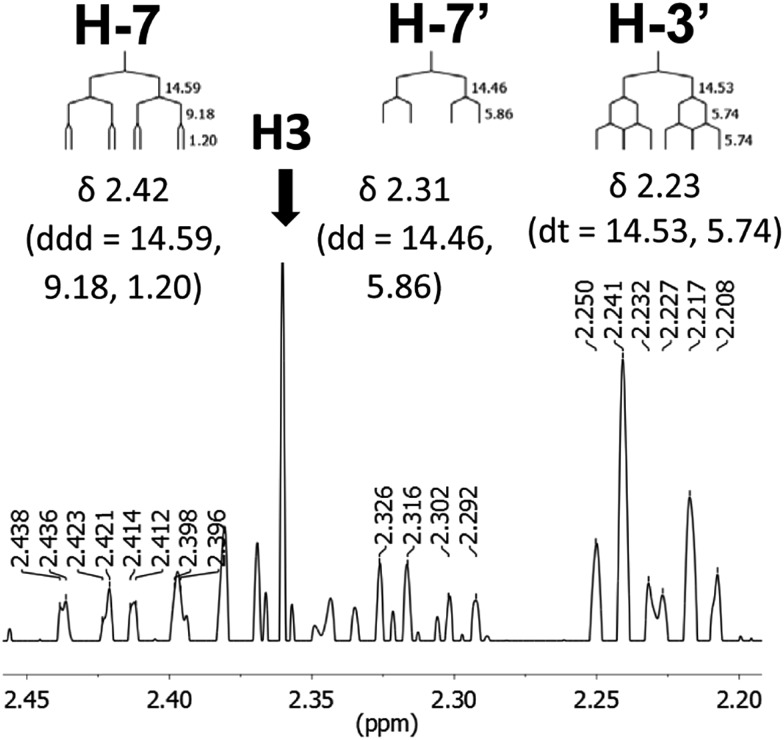
Mycothiazole (**88**) expanded ^1^H NMR spectra regions (CDCl_3_, 600 MHz) for H-7/7′ and H-3/3′ obtained from FIDs processed using second derivative/nonlinear fitting.

**Fig. 29:**
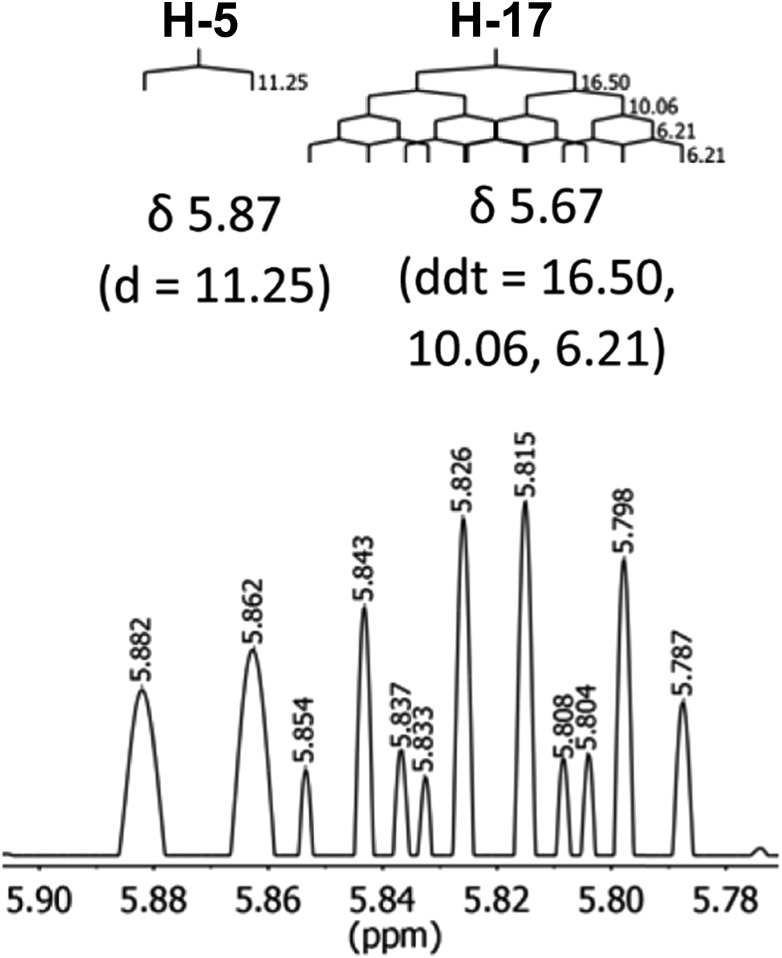
Mycothiazole (**88**) expanded ^1^H NMR spectral regions (CDCl_3_, 600 MHz) for H-5 and H-17 obtained from FIDs processed using second derivative/nonlinear fitting.

**Fig. 30:**
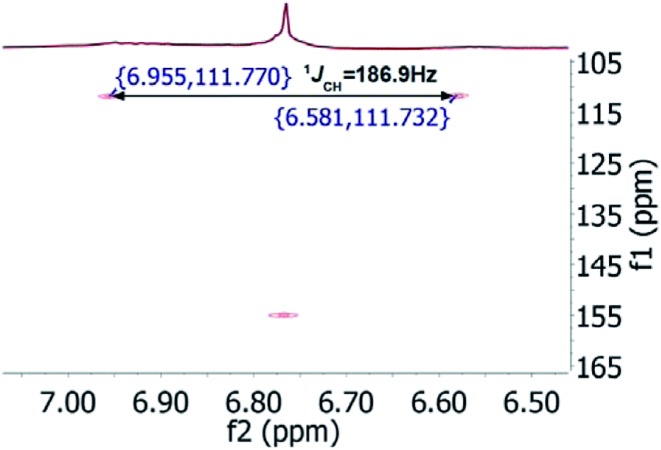
Mycothiazole(**88**) partial HMBC spectra (CDCl_3_, 500/125 MHz) obtained by classic work-up of FIDS but expanded to show the faint ‘breakthrough’ correlations used to measure ^1^*J*_C-11,H-11_ = 186.9 Hz.

Similar outcomes are shown in Fig. 28 and 29 that more accurately describe the coupling patterns of olefinic hydrogens (H-5, H-17) and aliphatic hydrogens (H-3′, H-7, H-7′). The previous data from measurement in CDCl_3_ reported most of these resonances as multiplets. Alternatively, analysis of these resonances by either first order or non-first order signal fitting accurately provided the eleven *J* values shown. These data should be useful in the future as new MYC analogues are isolated or synthesized. The value of obtaining and using HMBC-derived ^1^*J*_CH_ data to make functional group assignments for compounds possessing ratios of H/(C + Z) < 0.5 was recently demonstrated.^222^ It appears that accessing such data has become a “forgotten art”, yet the measurement shown in Fig. 30 illustrates that this process can be done accurately and rapidly when raw data is available. The coupling value shown here now provides a more accurate estimate of the ^1^*J*_C-15, H-15_ = 186.9 Hz *vs.* the published value of 194 Hz.^217^

As shown in the next section, there are other direct and indirect methods to obtain ^1^*J*_C,H_ values from reprocessed FIDs, representing another rationale for the collection and dissemination of raw NMR data.

## 6 New methodology

### 6.1 Data mining the one-bond heteronuclear coupling constant, ^1^*J*_CH_

Assembling and assigning the common ^1^H NMR data parameters is a typical prelude to linking nuclei (bond connectivity) by 2D and 3D NMR methods; a process familiar to chemists conducting integrated structure elucidation.^223^ The undisputed value of chemical shift for establishing the electronic environment of nuclei has driven the development of NMR instrumentation to higher fields to maximize dispersion. Assembling molecular structures by interpretation of HSQC and HMQC, the most widely-available heteronuclear 2D NMR experiments, gives direct bonding information of ^13^C–^1^H couplets. The latter are identified by the presence of cross correlations, but lost in the process is another powerfully informative parameter: the magnitude of ^1^*J*_CH_, itself. Out of a necessity to maintain the signal to noise (S/N) in heteronuclear correlation experiments, the latter is sacrificed by abolishing the couplets through ^13^C-broadband decoupling during acquisition of the FID. Nevertheless, ^1^*J*_CH_ can be recovered, as has been amply demonstrated through structure elucidation of numerous NPs, by the simple expedient of recording the FID with no ^13^C-broadband decoupling. The so-called coupled HSQC experiment replaces single cross correlations of each ^13^C chemical shift (or two, in the case of diastereotopic CH_2_ groups) with two component-cross-peaks, the ^1^*J*_CH_ C–H couplets, where the value of the coupling constant is revealed by their separation in Hz.

**Fig. 31:**
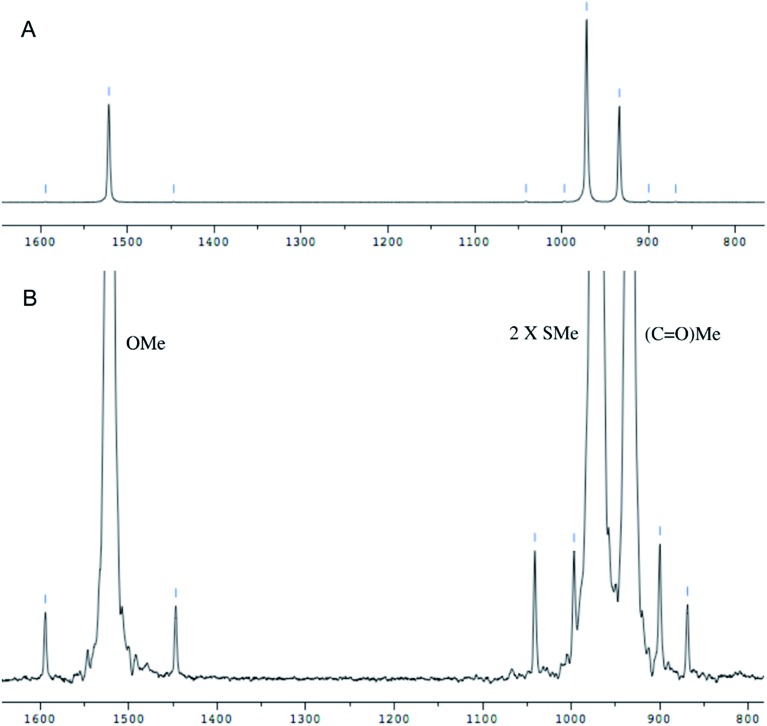
^1^H NMR spectrum of bis-(methylthio)-ester **94** (400 MHz, CDCl_3_). The *X*-scale is in Hz. (A) normalized *Y*-scale. (B) Vertical expansion of (A). Note, coincidence of the two *S*Me signals (2.43 ppm, s). Sample and spectra, courtesy of M. N. Salib (UC San Diego).

Often overlooked in ^1^H NMR spectra, is the cryptic presence of the one-bond heteronuclear coupling constants, ^1^*J*_CH_, seen as ‘^13^C-satellites’ of the ^1^H signals at the natural abundance of ^13^C, ∼1.1%. In fact, the utility of ^13^C satellites in ^1^H NMR spectra was recognized by Truner and Sheppard as early as 1959, when they analyzed the fine structure of the ^13^C satellites to determine the coupling constants of hydrogen nuclei of adjacent carbons that are chemically equivalent.^224^ Most likely, and especially for NP applications, the low abundance of the ^13^C satellite signals and the associated sensitivity challenge has been a major impediment for a broader implementation of this approach. Direct detection of ^1^*J*_CH_ from uncoupled or ‘gated-coupled’ ^13^C NMR spectra still requires inordinately large samples and/or X-nuclei direct detection cryoprobe instrument. While indirect detection of ^1^*J*_CH_ from HSQC spectra is relatively time-consuming, the ^13^C-satellites of ^1^H signals reveal heteronuclear couplings, in favorable cases, within the ^1^H NMR spectrum, requiring no special treatment beyond inspection, or facile post-acquisition processing of the FID at most. The extraordinary value of the ^1^*J*_CH_ magnitude and its application in structure elucidation is underestimated and can be summarized as follows:

(i) Hybridization at carbon. The magnitude of ^1^*J*_CH_ is directly proportional to the amount of s-orbital character (%s) in hybrid atomic orbitals (%s for sp^1^ = 50%; 33 1/3% for sp^2^; for 25% in sp^3^) that combine to form the molecular orbitals of sigma bonds. For olefins and arenes, unlike ‘normal’ aliphatic compounds, sp^2^-hybridized C have larger heteronuclear couplings (^1^*J*_CH_ ∼ 150–170 Hz), while the sp^2^-hybridized C in terminal acetylenes consistently exhibit the largest magnitudes of any ^13^C–^1^H couplets (^1^*J*_CH_ ∼ 250 Hz). For example, the terminal acetylene residue 3-hydroxy-2,2-dimethyloctynoic acid (Dhoya, first found in pitipeptolide (**89**)) from *Lyngbya majuscula*^225^ and several variants, from other cyanobacterial NRPS-PKS NPs^226^ is a group which shows an unremarkable ^1^H NMR chemical shift (1.96 ppm) due to diamagnetic shielding, but a large ^1^*J*_CH_ ∼ 250 Hz. A vexing technical issue in HSQC spectra of terminal acetylenes is the acetylenic correlation signal is often ‘missing’. This is due to the large deviation of ^1^*J*_CH_ in terminal acetylenes from the nominal value of the one-bond ‘J filter’ (^1^*J*_CH_ = 140 Hz) used in standardized parameters of the pulse sequence, but the cross-peaks can be recovered with appropriate re-parametrization. A combination of resonance energy and electronegativity effects (see below) leads to exceptionally large couplings for five-membered hetero-aromatic rings (1,3-oxazole, imidazole, thiazole, *etc.*), compared to arenes, which can be readily identified from the ^13^C-satellites of their ^1^H signals. For example, the H-5 signal (azole numbering) in each of the three 1,3-oxazole rings of the trisoxazole macrolide (**90**) from the nudibranch, *Hexabranchus sanguineus*, as well as that of the thiazole ring of jamaicensamide A (**91**) from the sponge, *Plakina jamaicensis*, have ^1^*J*_CH_ values of 198 and 190 Hz, respectively. It was no small feat that the ^1^*J*_CH_ could be measured from ^13^C-satellites of a 33 µg sample using a microcryoprobe at 600 MHz.
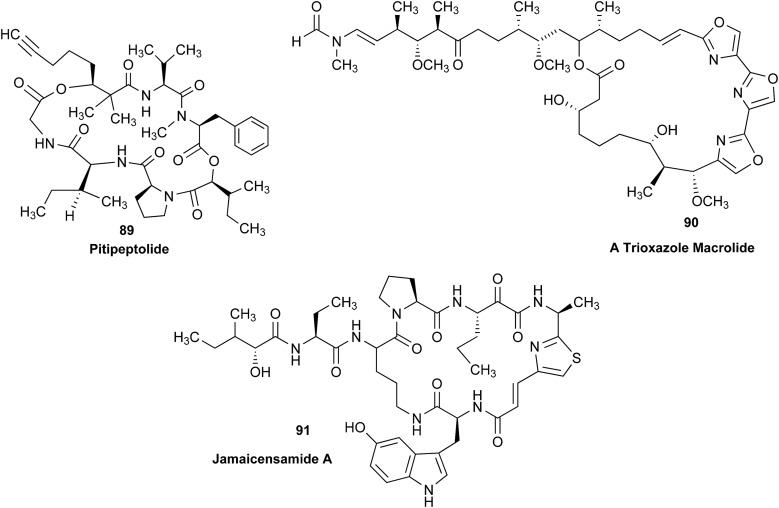


(ii) C–H groups associated with electronegative elements. Whereas the one-bond homonuclear coupling constants of unconstrained hydrocarbons and alkyl residues vary little from a nominal and almost invariant value of ^1^*J*_CH_ = 125 Hz, substitution by electronegative N, O, halogens and even the polarizable S atom, increases the magnitude to 140–150 Hz. For example, *N*-Me, *O*-Me and *S*-Me groups can be distinguished from *C*-Me groups (*e.g.*, an acetyl group, CH_3_(CO), *J* = 128 Hz) and assigned independently of the corresponding ^1^H NMR Me chemical shift in non-obvious examples where interpretation is equivocal, *e.g.*, the assignment of a methylthio group (*S*-Me) in varamines A and B, **92a**, **92b** (^1^*J*_CH_ = 140.5 Hz) and lepadines I (**93**, ^1^*J*_CH_ = 140 Hz).^227^ In the latter cases, elimination of alternative *C*-Me constitutional isomers was confounded by predictions of similar ^1^H NMR chemical shifts for the Me groups; a more common occurrence than generally assumed. An object lesson is provided by synthetic compound, **94** (Fig. 31),^228^ which has four Me groups – two attached to S, one to O and the fourth, to C. The assignment of the *O*-Me group from ^1^H NMR chemical shift, alone, is trivial (3.80, ppm), but the ^13^C-satellites also reveal the largest associated coupling constant (^1^*J*_CH_ = 147.6 Hz) of the four. The remaining three signals are clustered and not readily assigned by chemical shift, alone, however, their identities are revealed by heteronuclear coupling constants. The resonances of the two *S*-Me groups are overlapped and have essentially identical heteronuclear couplings (2.43 ppm s, 6H, ^1^*J*_CH_ = 141.3 Hz) that, incidentally, integrate for roughly twice the *O*-Me ^13^C-satellites. Therefore, the remaining Me signal, slightly more shielded group than the latter two, is associated with the smallest heteronuclear coupling, and can be assigned to the acetyl group (2.33 ppm, 3H, ^1^*J*_CH_ = 128.3 Hz).
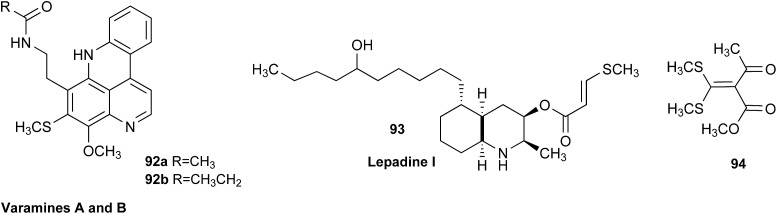


(iii) Identification and assignment strained 3-membered and 4-membered rings in monocyclic, bridged and fused polycyclic structures where, again, the coupling constants in cyclopropanes, cyclobutanes and heterocyclic small rings depart from a nominal ^1^*J*_CH_ = 125 Hz to magnitudes of up to ^1^*J*_CH_ ∼ 180 Hz in the case of a di- or tri-substituted epoxide (oxirane) found in meliatoxins A1 (**95a**) and B1 (**95b**) from *Melia azedarach*,^229^ or the oxetane ring of paclitaxel (**96**) *ex post facto* of the original X-ray structure.^230^ The latter method is particularly powerful as no other reliably and independently establishes ring size in cyclic NPs, and in many cases, can be used to resolve constitutional isomers (*e.g.*, the isomeric products of a Payne rearrangement). Finally, electronic and ring strain factors that contribute to the magnitude of ^1^*J*_CH_ are additive. For example, the ^1^H and ^13^C NMR spectra of the unique *trans*-chlorocyclopropyl ring in muironolide A (**97**), a macrolide from a Western Australian sponge, *Phorbas* sp., is associated with four large ^13^C–^1^H couplets (H-21, ^1^*J*_CH_ = 177 Hz; H-22a, ^1^*J*_CH_ = 173.4 Hz; H-22b, ^1^*J*_CH_ = 173.4 Hz; H-23, ^1^*J*_CH_ = 200 Hz)^231^ that uniquely identify strain and electron-withdrawing effects within the ring. A useful trend in the of ^1^*J*_CH_ of the diastereotopic CH_2_ group of the imidazolone ring found in the cyclic peptide, *N*,*N*′-methylenodidemnin A from the Caribbean cyanobacterium *Trididemnum solidum* observed, expanded by measurements of ^13^C-satellites in the ^1^H NMR spectra of several imidazolone and oxazolidine models.^232^ An unusual finding was that ^1^*J*_CH_ in the ^13^C–^1^H couplets of the diastereotopic CH_2_ are often non-equivalent and, therefore, dependent on relative orientation.
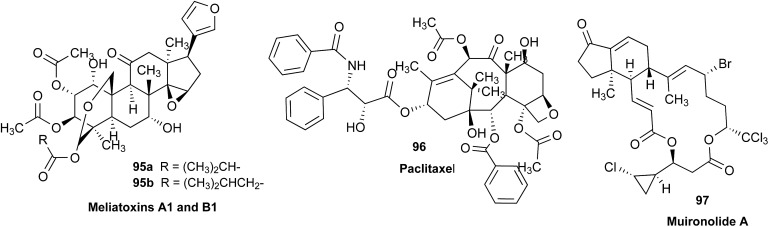


Exploitation of ^1^*J*_CH_ can be useful in alkaloid assignments; for example, the presence of a 2*H*-azirine ring (azacyclopropene) in dysidazirine (**98**),^233^ and related compounds^234^ is confirmed by observation of the exceptionally large coupling constant (^1^*J*_CH_ = 189 Hz) of the corresponding CH–C=N couplet. It is expected that the extraordinary structure of cyclopropylazetidinone (**99**), an ‘alkaloid’ obtained by Rainier and coworkers as an intermediate in the synthesis of natural pyrroloindolines and confirmed by X-ray crystal structure analysis, is expected to be associated with an unusually large ^1^*J*_CH_ for H-2 (5.85 ppm CDCl_3_),^235^ interesting to measure, to say the least (in the publication,^235^ the ^13^C-satellites [^1^H NMR, 500 MHz] are too weak to be visible in the current PDF print format of the ESI†).
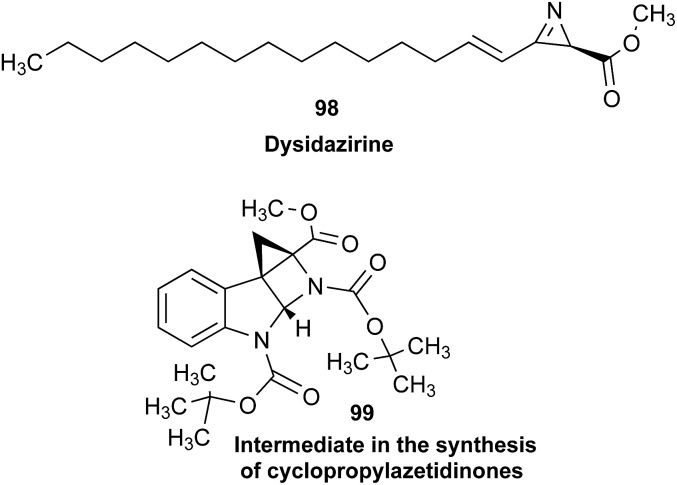


Extraction of ^1^*J*_CH_ values from ^13^C-satellites of ^1^H NMR spectra is limited by several instrumental and sample-related factors that militate against their observation. Nevertheless, access to the original FID of the spectrum can mitigate some of the difficulties in ways that are illustrated in three major groups:

(i) Poor S/N in ^1^H NMR spectra of small-sized samples. In order for the ^13^C-satellites to ‘rise’ above the noise level, a good quality ^1^H NMR spectrum of a ‘strong sample’ is required such that the signal due to the natural abundance of ^13^C in the sample exceeds the amplitude of random noise. With limited sample, this can be challenging, but as mentioned elsewhere in this review, the data content of the time-dependent periodic function that constitutes the FID is a fixed product of S/N and resolution: one can trade one for the other, to some extent, by judicious reprocessing. Careful use of apodization functions prior to FT of the FID may regain S/N at the expense of resolution (line width) to reveal ^13^C satellites that are invisible from first inspection and in printed documents such as PDF files in traditional ESI† format. As loss of resolution is almost always inconsequential for measuring ^1^*J*_CH_, except for very weakly dispersed signals, this can be an effective way to tease out important information from FID data made available in digital format.

(ii) Spectral overlap or complex multiplet structure. ^13^C satellites that exhibit complex multiplet structures, due either to overlaid homonuclear coupling (^*n*^*J*_HH_ with *n* = 2, 3, *etc.*), or symmetry-related reasons, may completely ‘disappear’ beneath the noise or be obscured by nearby ^1^H signals. Fortunately, only one half of the ^13^C satellite doublet signal needs to be observed as the ^1^*J*_CH_ is reconstructed from twice its separation from the dominant centroid ^12^C–^1^H signal (ignoring the slight isotope shift of the former). Here, a caveat should be stressed: the sample should be sufficiently pure that spurious impurity signals are not mistaken for genuine ^13^C satellite signals. Regrettably, with very noisy spectra, ‘there is no such thing as a free lunch’: little can be done if apodization of the FID, even at an extreme level prior to FT, does not result in reliable appearance and identification of the ^13^C satellites. In this case, salvaging the ^1^*J*_CH_ may only be achieved by re-recording the ^1^H NMR with a more concentrated sample, in which case it is far preferable to record the coupled HSQC.

(iii) Line-shape. In order to separate the ^13^C-satellites from the base of the dominant ^12^C–^1^H signal, good NMR signal line shape is required, especially at higher fields.

For the foregoing reasons, readily measurements of ^1^*J*_CH_ from ^13^C-satellite signals is most practical from ^1^H NMR signals where signal complexity does not exceed singlet or doublet splitting. Here, the low-abundance ^13^C–^1^H couplets can be exploited best, delivering valuable new information on electronic environment, hybridization and ring strain for molecular structure determination of an NP. All this, from no more than a re-processed ^1^H NMR spectrum, accessed from archived digital FID data. An enhanced HSQC experiment for an accurate and more rapid assessment of one-bond proton-carbon coupling constants has been reported very recently.^236^

A variety of 2D NMR methods have been developed that enhance the utility of C,H-coupling information in NP research, covering both direct (^1^*J*_C,H_) and longer-range (^≥2^*J*_C,H_) coupling relationships. Examples are the ASAP variant of HSQC^237^ and the establishment of NOAH supersequences^238^ for accelerated acquisition, non-uniform sampling (NUS)^239^ and CRAFT 2D processing^240^ techniques for enhanced resolution, as well as LR-HSQMBC and HSQMBC-TOCSY for improving the detection of long-range correlations.^49^,^50^

### 6.2 New analysis of published data by optimal processing of the FID

NMR data can provide a wealth of information regarding a given chemical structure and much of this information is frequently overlooked. For instance, coupling constants (*J*) provide key information, especially in configurational aspects. Consequently, valuable details about structural identity are often misinterpreted and/or lost. In fact, deep analysis of a ^1^H NMR spectrum often obviates the acquisition of further experimental data and enables a more efficient use of the NMR spectrometer. In this regard, the availability of the raw NMR data plays a key role in both the verification of interpretation and the extraction of new information that otherwise is lost.

**Fig. 32:**
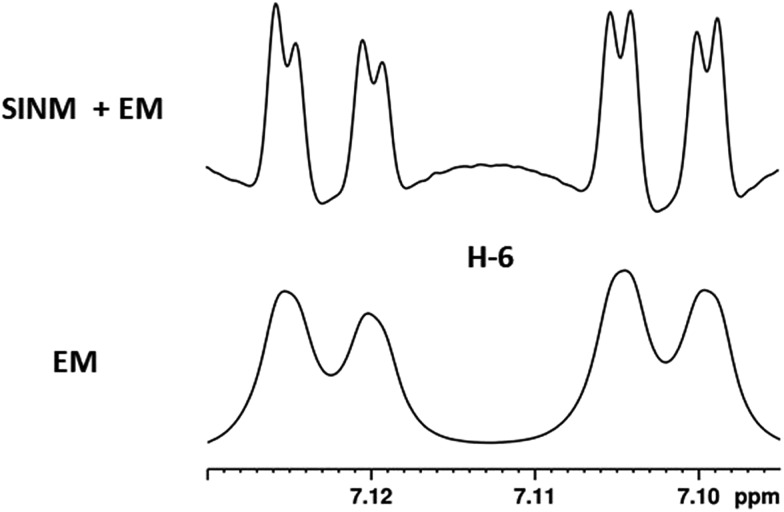
Comparison of ^1^H NMR spectra processed with default settings (*i.e.*, EM with an LB value of 0.3 Hz) *vs*. the use of line shape enhancement (*i.e.*, SINM plus EM with an LB value of 0.3 Hz) for H-6 of butein (**100**) at 7.11 ppm.

This affirmation can be illustrated by the measurement of long-range (^4–5^*J*) coupling constants such as the ones between a hydrogen nucleus of an aromatic ring and those of a side chain. To access this information, FIDs should be multiplied by resolution-enhancing window functions such as Gaussian or sinebell. This is enabled by the availability of the digital NMR raw data. This approach has been used by Lima *et al.* (2015 and 2016),^241^,^242^ Pederoso *et al.* (2008),^243^ Amoah *et al.* (2015),^244^ and da Silva *et al.* (2015),^245^ for establishing the connectivity of aromatic and side chain moieties of several NPs. In the case of butein (**100**),^242^ for example, the shifted sinebell multiplication (SINM) followed by an exponential multiplication (EM) of the FID with a Lorentzian line broadening factor of 0.3 Hz instead of the simple EM (default setting on most NMR spectrometers; see also Fig. 5) prior to Fourier transformation revealed a small additional coupling constant (*J* = 0.5 Hz) correlating H-6 with H-α (Fig. 32). This finding is supported by the reciprocal analysis of the signal of H-α. Thus, the molecular connectivity between the aromatic ring with the double bond side chain in butein could be established based only on the ^1^H NMR spectra without the need of two-dimensional (2D) NMR experiments.
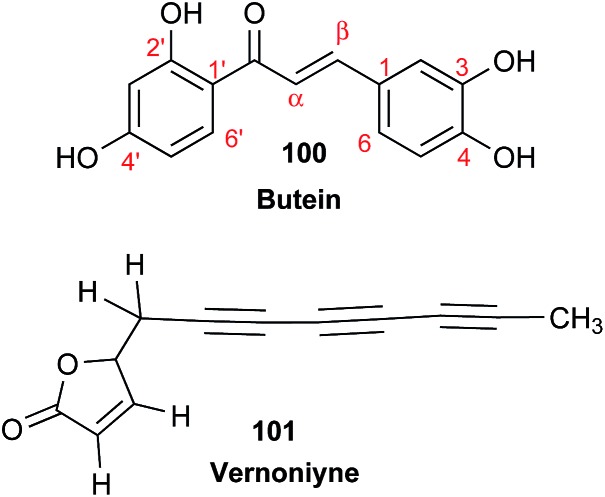


**Fig. 33:**
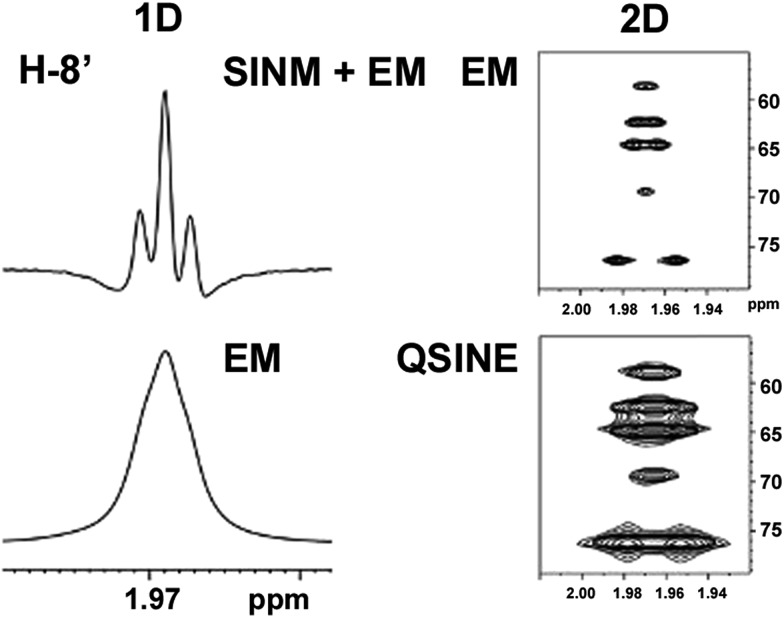
Comparison of ^1^H NMR spectra processed with default settings (*i.e.*, EM with an LB value of 0.3 Hz) *vs.* the use of line shape enhancement (*i.e.*, SINM plus EM with an LB value of 0 Hz) for H-8′ of vernoniyne (**101**) at 1.97 ppm. Comparison of typical long-range ^1^H–^13^C correlation map processed with 1 K per 512 data (*i.e.*, without zero-filling) in F2 and F1, respectively and QSINE as window functions in both dimensions and higher processed using EM of 0.0 Hz on both dimensions and zero-filling to 4 K per 1 K in F2 and F1, respectively. This is just a simple example, there are many other advanced ways to process 2D correlation maps.

Furthermore, the processing of the raw NMR data can bring information from even longer conjugated chains. The polyacetylenes found by Buskuhl *et al.*^246^ and Pollo *et al.*^247^ are good examples of this application. In these cases, the employment of enhanced line shape processing permitted the correlation of a long distance coupling (^9^*J*; Fig. 33). Such long-range correlations can only be observed in situations where the electronic density is high, such as on conjugated triple bonds. Thus, advanced raw NMR data processing permitted not only connecting moieties, such as in **100**, through the correlation of H-1′ and H-8′, but also determining the presence of triple bonds in the polyacetylene structures as in vernonyine (**101**).

The same strategy can be used in 2D NMR correlation maps, such as HMBCs. The original file, containing the raw NMR data is of great importance once it allows counter level editing, which permits observation of a correlation or lack of one. The advanced processing of HMBC allowed the unequivocal establishment of the ^13^C NMR chemical shift assignments from C-2′ to C-7′ from the long-range ^1^H–^13^C correlation of H-1′ and H-8′ in these polyacetylenes (Fig. 33).

NMR-based techniques^248^ have enormous potential for NP investigation since they provide unique and comprehensive information for structure determination and dynamic of chemical compounds. Therefore, advanced NMR processing strategies can be valuable on those spectra acquired directly from raw material as in gel-like systems through HR-MAS NMR spectroscopy,^249^,^250^ because in these cases the spectral resolution is naturally lower due to restricted molecular mobility.

Nevertheless, the quality of the results from advanced NMR data processing depends on spectra being acquired with sufficient signal-to-noise ratio (S/N). This requires an appropriate number of scans and high time-domain resolution (at least 64 K data points). Additionally, the 1D spectra and *n*D correlation maps need to be processed using a large number of zero-filling (at least 128 K in 1D and 4 K per 1 K in 2D).

### 6.3 In-depth analysis of ^1^H and ^13^C NMR data of smenospongidine

Smenospongidine (**102**), a biologically active quinone sesquiterpenoid, was isolated from the sponge *Smenospongia* sp. by Kondracki and Guyot in 1989.^251^ The authors proposed the structure of **102** based on analysis of HRMS, ^1^H NMR and ^13^C NMR data, but omitted carbon chemical shift data from the manuscript. In 1992, Rodríguez *et al.* published the first tabulated ^13^C NMR data of **102**.^252^ In the intervening years, **102** was isolated from various sponge sources^253^–^258^ each reporting close agreement to the published data. In 2002, an enantioselective total synthesis of **102** was reported by Ling *et al.*, which, in addition to the usual statement about good agreement with published data, was accompanied by ESI† with ^1^H and ^13^C NMR spectra of the synthesized molecule.^259^ Recently, the Williams' group also isolated **102** from *Dactylospongia elegans* and found significant discrepancies between their spectral data, and previous reports. Herein, they summarize the discrepancies and report corrected data for **102**. This case story demonstrates the value of depositing raw NMR data by showing how errors (omission, assignment, typographical, *etc.*) propagate through the literature when forced to rely on reproduced, tabulated, or listed data. Moreover, it points out the difficulties in locating original NMR data decades after publication. Although **102** is a specific example, the problems are nonetheless widespread and persistent in the literature.
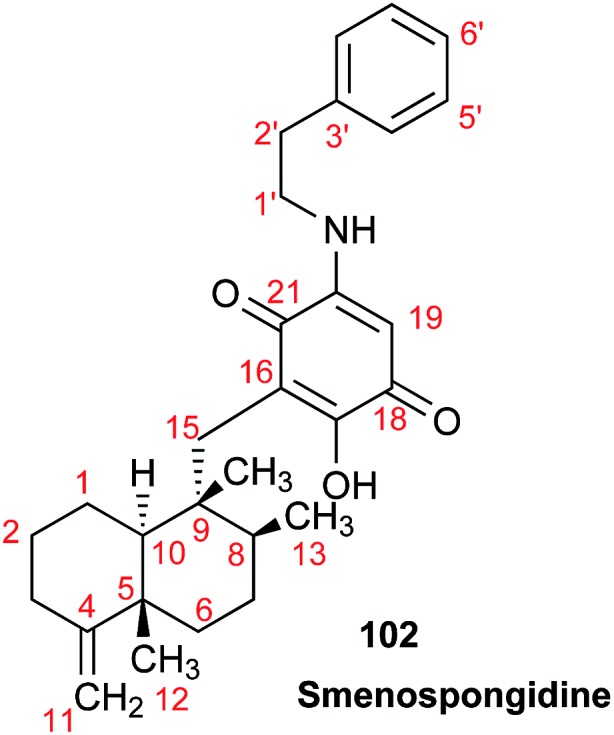


The spectral data of **102** obtained from this study are tabulated (Table 4) against those reported by Rodríguez *et al.*, who did not report signals for the non hydrogen-bearing carbons C-10, C-16 and C-18 in **102**. Aside for that, the only major difference (>2 ppm) between the two ^13^C NMR data sets occurs at C-20 (150.0 *vs.* 154.7 ppm), with Williams' value of 150.0 being more consistent with their data for the C-5 epimer of **102**. The partial ^1^H NMR data reported in that manuscript has two main inconsistencies. First, a singlet reported at 5.41 ppm assigned as the hydroxyl hydrogen may instead be the olefinic hydrogen H-16 in the quinone ring. Second, a doublet at 0.77 ppm assigned to methyl hydrogens (H-13) is more characteristic of H-10, an axial methine hydrogen at the *trans*-decalin junction in quinone-containing analogs of **102** with identical configuration.^251^ With access to the original spectra, these issues of unreported or possibly misassigned signals are easy to resolve. For example, the last issue (H-10 *vs.* H-13) could possibly be distinguished by the integrals, multiplicity (d *vs.* dd) or the magnitude of observed coupling as the axial methine H-10 should display a larger *J* value (>10 Hz), due to coupling with the neighboring axial hydrogen (H-1), than the typical 7 Hz observed from methyl doublets. It should be noted that it is highly unlikely that even contemporary spectra exhibit adequate resolution or sufficient peak-picked expansions to resolve the matter when disseminated as ESI† material in the currently customary PDF format.

**Table 4:** Comparison of ^1^H and ^13^C NMR data of smenospongidine (**102**) in CDCl_3_

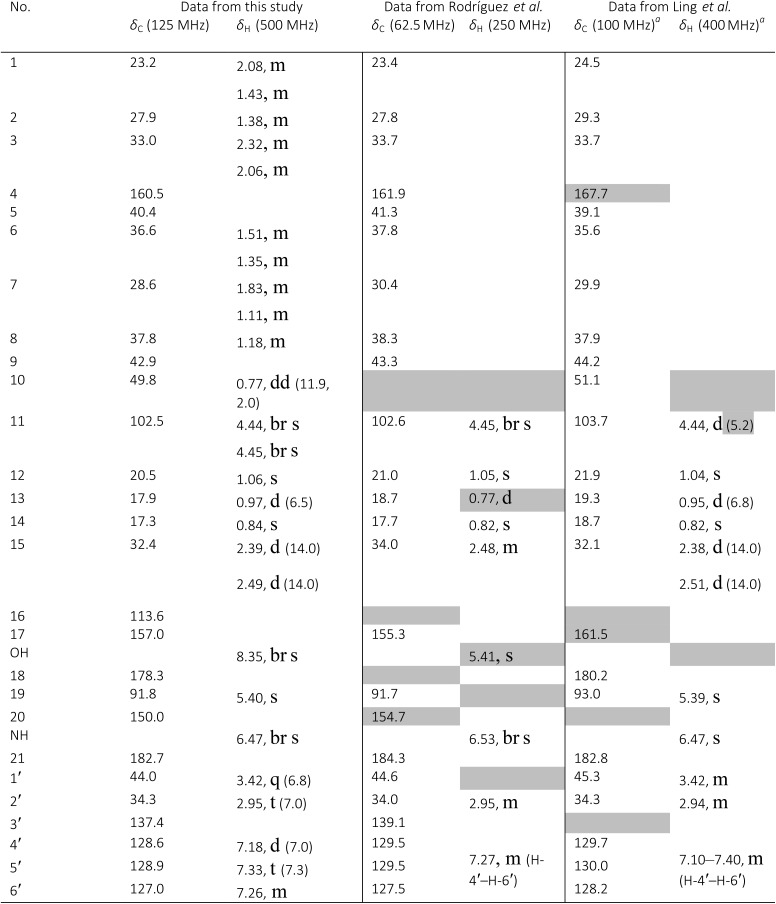

^*a*^Assignments were made by matching the hydrogen and carbon nuclei with the closest reported chemical shift values. None of the reported chemical shift values could be assigned to carbon nuclei at positions 16, 20, and 3′, whereas the signals at 69.0, 65.9 and 31.0 ppm were deemed extraneous.

To resolve these issues and clarify the identity of Williams' sample of **102** with only hydrogen and carbon data of the sample on hand, the reported synthesis was repeated. As is typical, no specific hydrogen or carbon assignments are reported in the manuscript describing the synthesis of **102** for the listed chemical shifts, so they have been assigned as seemed best for this comparison (Table 4) with the major differences highlighted in gray. Unfortunately, this data raised more questions. Their listed ^1^H NMR data on S19 does not include the signal for H-10, the hydrogen at the A/B ring juncture possibly misassigned by Rodríguez *et al.*, but does include a signal at 0.97 ppm (*d*, 3H, *J* = 6.0 Hz), here assigned to H-13; a signal missing altogether from Rodríguez *et al.*'s paper. Despite the inclusion of ^1^H NMR spectra in the ESI,† the presence of these signals could not be conclusively confirmed because of the unavailability of an appropriate expansion of the spectrum. Other issues apparent from the listed ^1^H NMR values are the mischaracterization of resonances here assigned to the terminal exocyclic alkene H-11 (reported as 4.44 ppm, *d*, 2H, *J* = 5.2 Hz) and H-2 (2.94 ppm, 2H, m). The latter resonance should be a triplet as the hydrogens responsible for the signal are adjacent to only two equivalent hydrogens, while the characterization of the 4.44 ppm (d, 2H, *J* = 5.2 Hz) resonance is clearly erroneous, as a *J* value of 1.2 Hz, typical of the coupling between two non-equivalent hydrogens of the exocyclic terminal alkene, can be calculated from peak-picking in the ESI.† The ^13^C NMR spectrum provided in the ESI† and the chemical shift values extracted from the spectrum raise further questions on the interpretation of the NMR spectral data. Twenty-seven unique ^13^C NMR resonances are expected for **102**. The ESI† of Ling *et al.* lists 25 signals for **102**, omitting two carbonyl signals. Of these 25 signals, only 7 out of 12 required sp^2^ carbon signals are reported and the list includes signals at 69.0 and 65.9 ppm clearly inconsistent with the proposed structure as it lacks oxygenated sp^3^ carbons. The ^13^C NMR spectrum with the poor signal/noise included in their ESI† sheds some light on the situation, but also raises questions as it includes the two carbonyl signals omitted from their list. The two carbonyl signals are labeled at 182.8 and 180.2 ppm but both appear between the chemical shifts of 181 and 182 ppm in the ^13^C NMR spectrum perhaps due to peak-picking errors.

There is little question **102** was isolated or synthesized, as published in these articles. The Williams' group has in fact synthesized **102** from ilimaquinone using the method described by Ling *et al.*, and independently confirmed the structure. Throughout the process, the corresponding authors of those reports graciously offered assistance and searched for their original data at Williams' request, but decades later were unsurprisingly unable to locate it. The difficulties of individual labs or departments maintaining NMR records over 40 years are significant. The staff at University of Hawaii, Manoa, receives frequent requests for copies of NMR data generated by the late Paul Scheuer and Richard Moore with a success rate of less than 50%. Most recently, a request for data on the cyanobacterial compound micromide could not be fulfilled due to degradation of the CD backups. The fact remains that our community's reliance on tabulated or summarized data introduces the possibility of a litany of errors into the literature. Availability of raw NMR data would undoubtedly play a major role in curbing propagation of these errors.

## 7 Other nuclei

### 7.1 Fluorine: paramagnetic and diamagnetic effects

Fluorine is commonly used in organic chemistry, especially in medicinal chemistry and materials, because it is both small and much more electronegative than the H and C atoms that make up a good portion of organic compounds.^260^–^262^ Fortunately, ^19^F is 100% naturally abundant and NMR active. ^19^F has a gyromagnetic ratio close to that of ^1^H, and a nuclear spin of ½, but covers a much larger chemical shift range than ^1^H (∼400 ppm for organofluorines), meaning that signals tend to be well resolved.^263^ Unfortunately, the shifts of these fluorines can be difficult to assign if multiple fluorines on a molecule are in similar environments.

The shielding that leads to observed ^19^F shifts arises, in part, from both diamagnetic, and paramagnetic effects. The diamagnetic term is based on the electron density around the nucleus, while the paramagnetic term is based on the excitation of electrons in fluorine's p orbitals (not an issue for ^1^H). Consequently, ^19^F NMR shifts cannot be thought of as reporting on the “nakedness” of the nucleus in question, as ^1^H NMR and ^13^C NMR shifts often are. Computational work by Christe and coworkers confirmed that the paramagnetic shielding is significant, and can be crudely estimated by the computed anisotropic shielding, although this value is dependent on interactions between the fluorine atom and solvent.^264^

These differences between ^19^F and ^1^H/^13^C shielding contribute to the difficulty of assigning ^19^F signals, and associated data reporting issues and errors in assigned structures. *E.g.*, Burdon and co-workers synthesized functionalized perfluoroanthracenes and, based on the ^19^F NMR spectra of the products, decided that they were able to substitute “mainly or entirely in the 2 position”.^265^ Although ^19^F chemical shifts and splitting patterns were discussed in the text, no spectra or FID data were provided. In a subsequent study by Baker and Muir, computational results indicated that the initial experimental data more closely matched computed data for products of substitution at the 9 position, but direct comparisons with the experimental data was not possible and ambiguity about the structures still remains.^266^ This ambiguity could be resolved through a comparison of raw data with that generated from higher level quantum chemical computations. There are many more recent examples in which only ^19^F shifts are reported, with no spectra reproduced or raw data made available. It is hoped this situation will change soon, especially given the rise in importance of fluorine-containing organic molecules.^260^–^262^

### 7.2 Fluorine and its role in ADME

A growing area of research interest in the NP community is the generation of “non-natural NPs” by using synthetic biology approaches.^267^,^268^ The idea is to use the privileged scaffolds^269^ afforded by nature, and modify them to incorporate moieties and/or atoms not commonly found in NPs.^270^–^273^ In particular, the incorporation of an F atom is highly desirable, likely due to its positive impact on the biodynamic properties of biologically relevant molecules. Such analogues may affect one (or more) properties, such as protein ligand recognition and interaction, absorption, distribution, metabolism, elimination and toxicity (ADME-Tox).^262^,^274^–^276^ Moreover, as true NPs, organofluorine compounds are exceedingly rare, with less than ten reported.^276^ Hence, the way in which this atom affects a NP's biological activity and/or spectroscopic properties is rarely explored.

The chemical properties of ^19^F, including the atomic radius, electronegativity and polarizability of the C–F bond^262^,^275^ all contribute to its use in a suite of fields (*i.e.*, pharmaceutical industry, organic materials, and agrochemicals).^277^ In addition, the magnetic properties of the ^19^F nucleus, outlined make this nucleus an important tool for studying relevant biological processes particularly *via* the use of NMR, in the study of structure and function of biomolecules, enzymatic mechanisms, metabolic pathways, and ligand protein recognition.^278^,^279^

Some NP groups are striving to incorporate a fluorine atom.^271^–^273^,^276^,^280^–^284^ While most NP chemists are quite adept at analyzing NMR data, there are some spectroscopic properties of the molecule that change, sometimes dramatically, upon incorporation of ^19^F. As such, having the raw NMR data available serves to educate this research community on how to work with this nucleus in structure elucidation. For example, due to the nuclear spin of ½, the ^19^F nucleus couples to ^1^H and ^13^C, yielding signals with characteristic splitting patterns, many of which can be analyzed to further verify (or refute) a potential structure. Moreover, due to the high gyromagnetic ratio, the dipolar couplings are stronger, giving origin to enhanced ^1^H–^19^F NOE effects. Finally, the coupling constants (*J*_CF_) for ^13^C–^19^F are quite large (up to 250 Hz), providing information about the location of the F atom and the connectivity of adjacent atoms.^278^,^285^ In fact, these large *J*_CF_ couplings are very helpful in structure elucidation, akin to using HSQC data to assign how a ^13^C signal can be correlated with its attached ^1^H signals.^280^,^286^ Additionally, relatively simple experiments, such as a ^1^H decoupled ^13^C experiment, will display splitting due to the ^13^C–^19^F coupling, and upon first inspection, such data may be quite foreign, especially to a student. In summary, with respect to incorporating ^19^F into a NP, some changes to the NMR spectra are modest, while others can be quite profound and/or even unanticipated; access to the raw NMR files would facilitate a more thorough evaluation and dissemination of such data.

**Fig. 34:**
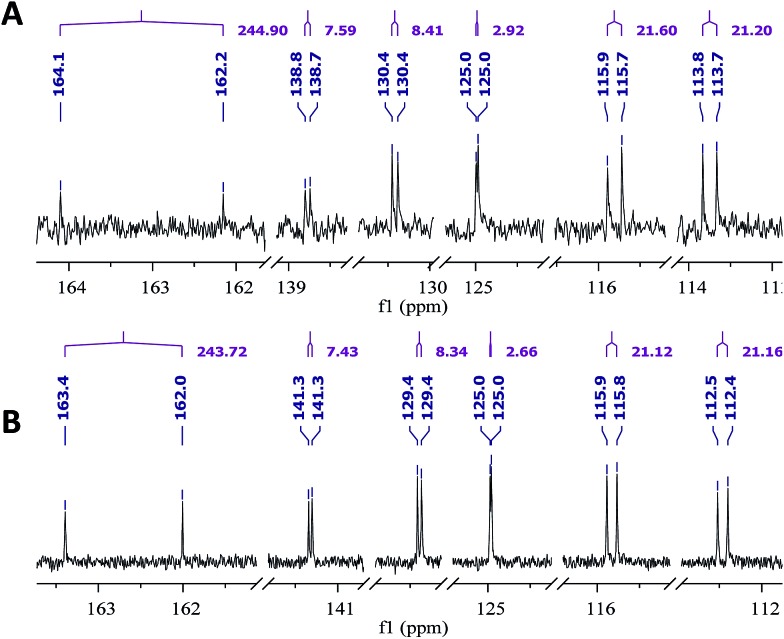
The ^13^C NMR spectrum of the aromatic region of (A) *m*-F-l-phenylalaninol standard (125 MHz in MeOH-*d*_3_), and (B) *m*-F-phenylalaninol in *m*-F-Pheol-alamethicin F50 (175 MHz in MeOH-*d*_3_). The prominent doublet (with a *J*_CF_ of ∼243 Hz) indicated the point of attachment of the ^19^F in the molecule. The other aromatic ^13^C signals all display doublets due to long-range coupling to this ^19^F.

**Fig. 35:**
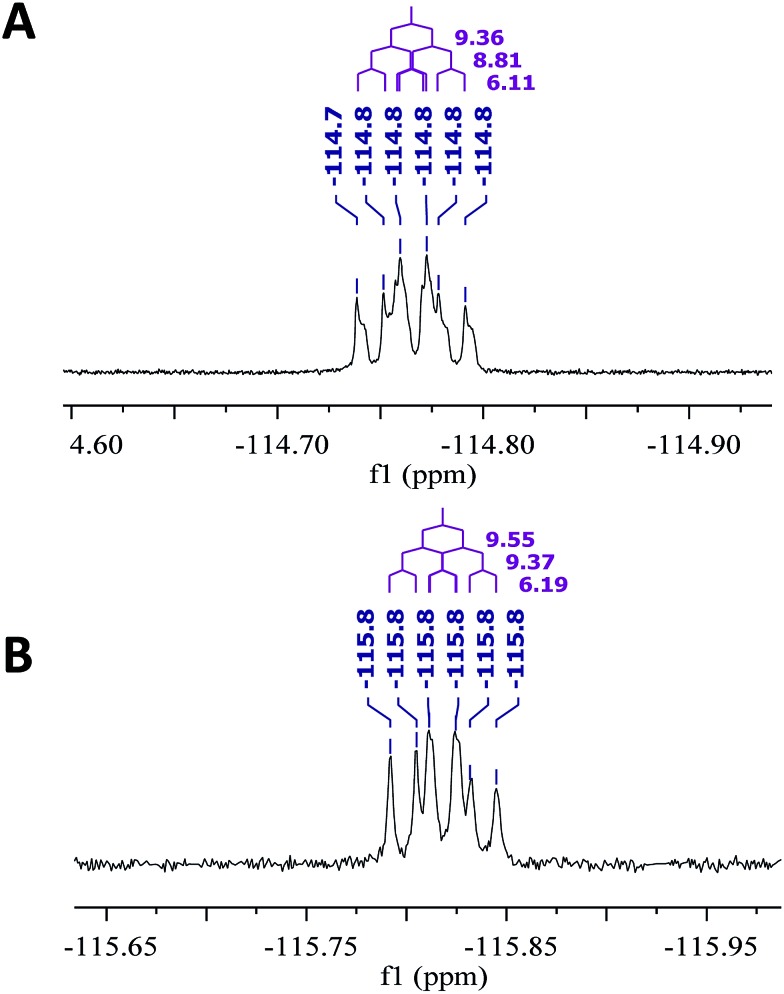
The ^19^F NMR spectrum of (A) *m*-F-l-phenylalaninol standard, and (B) *m*-F-phenylalaninol in *m*-F-Pheol-alamethicin F50. The observed coupling constants are for *J*_HF_. Running the ^19^F NMR experiment is a straightforward way to verify its incorporation. Both spectra were obtained at 470 MHz in MeOH-*d*_3_.

A recent example highlights the value of ^19^F NMR in structure elucidation, where two fluorinated peptaibols (analogues of alamethicin F50) were biosynthesized *via* a site directed building incorporation approach.^280^ In that study, *Trichoderma arundinaceum*, a well know alamethicin F50 producer, was fed with fluorinated building blocks (*o*/*m*/*p*-F-DL-Phe), and the biosynthesis of the fluorinated analogues was monitored *via in situ* MS and ^19^F NMR. The structure elucidation of the fluorinated analogues was carried out using a set of spectroscopic techniques, including ^1^H, ^13^C, and 2D NMR data. The incorporation of fluorine in the final product was confirmed by ^19^F NMR, analysis of the prominent ^13^C–^19^F *J*_CF_ values in the ^13^C spectrum, and by comparison of these data with those obtained for the synthesized standards (Fig. 34 and 35). The close match between the ^19^F and ^13^C NMR data of the synthesized monofluorophenylalinols (MW 165) and that of these moieties within the large peptaibols (MW > 1900) is remarkable. While members of this research team have performed thousands of NMR experiments over the years, the ^19^F NMR experiment was somewhat foreign. However, those data were extremely straightforward to analyze, and it is easy to envision deriving value from sharing those raw NMR files.

As noted previously, fluorine containing secondary metabolites are extremely rare in nature.^276^,^287^,^288^ Thus, when they are reported, thorough peer review is needed to insure the validity of the structure, (another compelling argument for the sharing of raw NMR data). A recent report highlights where some knowledge about ^19^F NMR would have likely prevented a mistake in the literature.^289^ The organofluorine compound [3-(3,5-di-*tert*-butyl-4-fluorophenyl)propionic acid] was reported isolated from a *Streptomyces* sp. TC1,^289^ which suggested the existence of an enzyme capable of mediating an aryl fluorination reaction. This report attracted the attention of two different groups, who *via* synthesis of the putative fluorinated natural product, and based on the analysis of ^1^H, ^19^F and ^13^C NMR spectra, both demonstrated the absence of fluorine in the secondary metabolite.^290^,^291^ While those follow up studies essentially refute the initial study, perhaps a more thorough analysis of the NMR data at peer review, including examination of raw NMR data, would have prevented the need for such research.

These examples show the importance of a detailed analysis of the NMR data, both when striving to generate fluorinated analogues and if/when naturally occurring organofluorine compounds are reported. A solid understanding of the NMR properties of the ^19^F nucleus is needed to rationalize the structure elucidation, and the raw NMR files would serve to both document and disseminate these information, possibly giving fodder for more detailed analysis as more advanced tools are developed.

### 7.3 The complex ^19^F NMR spectrum of 4,4-difluorinated proline

Amino acids and peptides are important lead compounds in drug discovery. However, such compounds usually undergo some form of structural modification before they can be considered viable drugs. Fluorination is one way to achieve this.^292^ The benefits of fluorination in terms of ADME properties are well known, and have been outlined in the previous section. In addition, fluorination can potentially enhance the target-binding properties of lead compounds, through conformational control.^293^ For example, fluorinating the 4-position of the amino acid, proline, can effectively stabilize either the *exo*- or the *endo*-pucker, depending on the fluorine configuration (an example of the “fluorine gauche effect”).^294^ This conformational biasing of proline has been exploited in the design of collagen mimetics,^295^ enzyme inhibitors,^296^ and organocatalysts.^297^

**Fig. 36:**
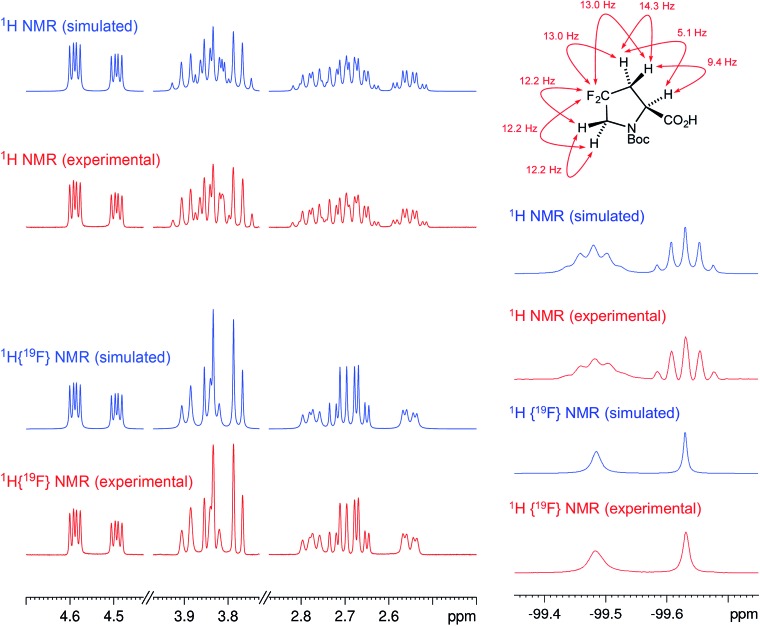
Partial ^1^H, ^1^H-decoupled ^19^F, ^19^F, and ^19^F-decoupled ^1^H NMR spectra of *N*-Boc-4,4-difluoroproline (**103**), showing all of the ring-attached atoms in each case. Twin sets of signals are observed due to the presence of Boc rotamers. The indicated *J*-values (top) correspond to the major rotamer of **103**.

Surprisingly, the closely related scaffold 4,4-difluoroproline has been little studied. The derivative **103** (Fig. 36) has previously been synthesized, and the ^1^H and ^19^F NMR spectra of this compound have been recorded,^298^ but these NMR data were reported only in condensed form with most signals simply described as multiplets, and no raw NMR data were made available at the time of publication. It would seem to be worthwhile to undertake a full analysis of the NMR spectra of **103**, in order to ascertain all of the *J*-values and thereby gain information on the conformational behavior of this compound.^299^
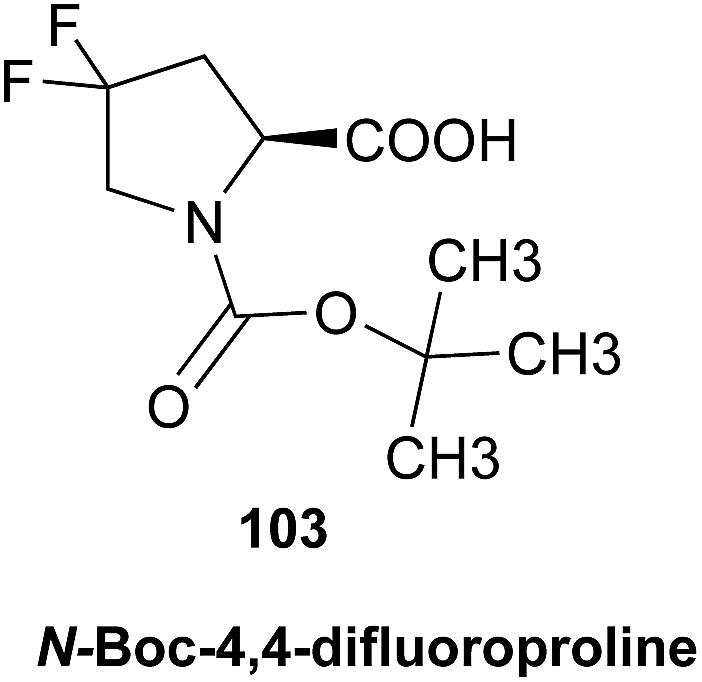


Accordingly, the Hunter group recently synthesized **103** following a published protocol,^298^ and re-acquired the ^1^H and ^19^F NMR spectra (Fig. 36). The spectra are complicated by the presence of Boc rotamers, giving twin sets of signals and possibly explaining why a full analysis was not reported previously.^298^ With raw data now in hand, Hunter and co-workers performed an in-depth analysis of the spectra through DAISY simulations, and this revealed an unusual pattern of *J*-values of **103** (Fig. 36). The two diastereotopic fluorine atoms of **103** have identical chemical shifts; hence, the fluorine atoms do not couple to one another, and together they cause each of the signals corresponding to the four vicinal hydrogens to be split into a higher multiple of an *n* + 1 triplet. Nearly identical sets of *J*-values are observed for both rotamers of **103**. Finally, Hunter and co-workers validated their analysis by also acquiring ^1^H-decoupled ^19^F and ^19^F-decoupled ^1^H spectra (Fig. 36), which were also found to be accurately simulated using the same *J*-values.

This elucidation of the *J*-values of **103** (Fig. 36) is a first step towards understanding the conformational behavior of this potentially valuable fluorinated building block.^299^ This information may inform the ongoing development of drugs and bioprobes that contain conformationally-biased proline residues.

### 7.4 Nitrogen: an underrepresented nucleus in the structural investigation of natural metabolites

Nitrogen containing metabolites occur naturally in essentially all terrestrial and marine organisms, and many of these compounds exhibit important biological functions related to their *N*-substitution. Unlike other nuclei, and despite the importance of nitrogen in natural metabolites, the ^15^N NMR of these compounds is rarely reported. In most, the nitrogen is a biologically essential element. Therefore, while nitrogen plays an important role in NP chemistry and biology (*e.g.*, labeling of non-proteinogenic amino acids), the NMR-detectable stable isotope ^15^N represents a spin ½ nucleus which has very low natural abundance of only 0.35%.^300^ Lowering NMR sensitivity even further, ^15^N has a gyromagnetic ratio of only about 1/10^th^ of that of ^1^H. These intrinsic properties make ^15^N difficult to observe directly. However, its enormous chemical shift dispersion of *ca.* 800 ppm offers a powerful source of structural information. Additionally, the orientation of the nitrogen lone pair of electrons is sensitive to the chemical and magnetic environment of the rest of a molecule. This has large effects on the observed coupling constants with nearby hydrogens. These coupling constants can have relatively large values, both negative and positive in a Karplus relationship, and can be used as evidence to distinguish two identical planar structure that only differ in the orientation of the lone pair of electrons.

As NMR hardware, software, and experimental techniques have advanced, it has become possible to detect ^1^H–^15^N correlation of sub-milligram samples of NPs by using inversed-detected pulse sequences. Martin and Hadden^301^,^302^ as well as Marek *et al.*^303^ have provided excellent general guidance in their comprehensive reviews. While ^15^N chemical shifts are often determined indirectly using ^1^H detected HSQC and/or HMBC experiments to enhance sensitivity, this approach is limited in terms of precision and often also accuracy (lack of reference marker). While DEPT and INEPT based experiments for direct detection can overcome this limitation, they are not widely used and pose specific sensitivity challenges for nitrogen atoms that do not bear a hydrogen. A third approach for ^15^N detection is to use the CIGAR-HMBC experiment introduced by Hadden *et al.*^304^ and modified by Kline and Cheatham.^305^ By sampling a range of ^15^N–^1^H coupling constants in a single spectrum, the CIGAR-HMBC sequence minimizes the risk of missing key correlations.

Importantly, as new techniques emerge and become part of routine operations, preservation of the raw data also becomes increasingly important, as a means of safeguarding the valuable structural information of the ^15^N spectra. As such, raw data sharing of this heteronucleus is not only about the documentation of experimental information, but more importantly a means of expanding the utility of (^15^N) NMR in structural analysis and, thereby, enhancing the reproducibility of NP and chemical science.^306^ Additional rationales for the importance of preserving raw ^15^N NMR data relate to the methods, precision, and accuracy of ^15^N chemical shift reporting, the value of structural information encoded in ^15^N NMR spectra, and the relevance of the more abundant ^14^N nucleus for explaining ^1^H NMR spin–spin coupling networks.

### 7.5 Nitrogen: chemical shift referencing, accuracy, and precision

Unlike ^1^H and ^13^C chemical shifts, which are reported relative to TMS as the accepted reference, (frequently *via* residual solvent signals), ^15^N NMR has no widely accepted single compound that serves as the universal reference standard in both the small molecule and the biomolecular NMR communities. Currently, most reports reference ^15^N chemical shifts *via* liquid ammonia (NH_3_/NH_4_OH), either *via* direct measurement or by application of a series of frequency conversion factors. The benefit of referencing with NH_3_ is that the resonance appears in the high-field portion of the ^15^N spectrum, avoiding resonance overlap in the more populated lower field. This approach explains why almost all reported *δ*_N_ are positive numbers. However, liquid NH_3_/NH_4_OH is typically used as an external reference, and temperature will affect the calibration result by as much as 40 ppb per degree, which compares unfavorably with the 4 ppb per degree variation of TMS in organic solvents.^307^ This is one main reason why other reference compounds are used. Varying in solubility and the effort required for sample recovery, nitromethane (90% in CDCl_3_; IUPAC recommendation for both ^14^N and ^15^N), nitric acid, ammonium chloride, formamide, or ammonium nitrate are often used in NP research. Two ^15^N resonances exist in ammonium nitrate, and both are used as reference signals. Notably, in the NP literature, almost all ^15^N chemical shifts have been determined from the indirect dimension, *via* inverse (^1^H) detection. Collectively, this explains the substantial variation of reported *δ*_N_ values as a result of inconsistent referencing. Accordingly, the reported *δ*_N_ should be considered approximate values, and the availability of raw data is one element that could help resolve this situation.

Another significance of raw data in ^15^N NMR relates to the accuracy of *δ*_N_ values, which are affected by the following factors:^308^ (a) the magnetic susceptibilities of the solutions from which the compared *δ*_N_ values originate are typically not identical. (b) The nature of the lock substance introduces a systematic variation/error. When using D_2_O, ND_3_NO_3_ or similar NMR solutes to lock the field frequency ratio, line-widths will broaden, which causes *δ*_N_ variations in the range of 0.1 ppm. (c) The temperature will affect chemical shifts not only in ^15^N but also other nuclides. For ^15^N, 0.4 ppm variation will be observed in two experiments when they have 10 K difference in temperature.

### 7.6 Nitrogen: NMR structural information encoded in ^15^N NMR spectra

Although the ^15^N chemical shift range is almost four times wider than that of ^13^C, the most interesting range of ^15^N resonances of NPs falls into the 20 to 420 ppm window. Notwithstanding the very low inherent sensitivity, it is also difficult to generate excitation pulses that are short enough to cover a 300+ ppm spectral window effectively, especially at the relatively low observation frequency of ^15^N. On the other hand, with the exception of oligo-(cyclo-)peptides, NPs often contain only one or very few ^15^N nuclei. For these collective reasons, the F1 range of a 2D NMR experiment is preferably limited to a narrow spectral window. In order to achieve this goal, raw data become invaluable as researchers can search available data(bases) and/or perform *in silico* prediction with increased precision provided raw data is available. Martin and co-workers have demonstrated several such cases, using both an NMR database and a commercial algorithm to predict ^15^N chemical shifts with only 2 ppm error, while most unfavorable result produced a variation as large as 50 ppm difference.^309^ Even long-range ^1^H–^15^N coupling constants are accessible through this method, with analogous limitations of currently achievable accuracy. As predictor algorithms depend on spectra derived from (manually) interpreted data, this method will become more accurate if the calculations could be based on raw NMR data.

Consideration of ^15^N coupling constants adds another layer to structural elucidation of nitrogen-containing molecules. Although ^1^H–^13^C heteronuclear long-range couplings are generally uniform, the same cannot be said about the corresponding ^1^H–^15^N couplings. This is mainly due to the effect of the lone pair electrons of nitrogen. The direction of the C–H bond of a hydrogen that exhibits a long-range coupling to ^15^N can have a significant impact on the value of the ^1^H–^15^N coupling constant. When the C–H bond direction is synclinal to the orientation of the lone pair, the ^1^H–^15^N couplings tend to be stronger and its long range HMBC correlations are detected readily. In contrast, when the C–H bond direction is anticlinal, the couplings will be much weaker and are more difficult to observe in HMBC experiments. The large variation of long-range ^1^H–^15^N couplings makes it more difficult to observe all ^1^H–^15^N correlation in a single HMBC experiment, because of the challenge in optimizing the magnetization transfer delay between ^1^H and ^15^N. So far, a universal approach has not been established. In current practice, the coupling values are either predicted *in silico*,^309^ or two different coupling values are chosen as distinct magnetization transfer delays implemented into two HMBC experiments.^310^

While ^15^N is the primary isotope related to the acquisition of nitrogen NMR spectra, it should be pointed out that the prevalent nitrogen isotope, ^14^N, also plays a role in NMR spectra of natural metabolites, namely in the spectral interpretation of ^1^H NMR spectra. One recent example is the observation of a ^1^H–^14^N coupling in the ^1^H NMR spectrum of ambiguine N isonitrile,^311^ a hapalindole alkaloid (**104**). This was discovered when analyzing preserved raw data *via*^1^H iterative full spin analysis (HiFSA) with the PERCH software tool, an algorithm based on quantum mechanical calculations and iterative fitting procedures.^62^
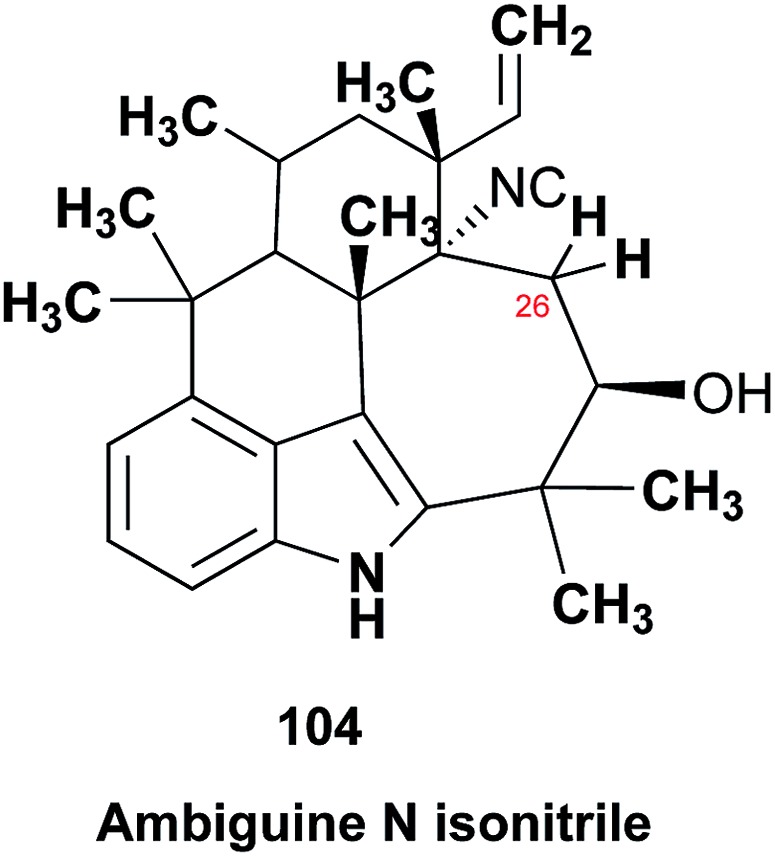


The signal of the axial hydrogen, H-26a shows an unexpected and rather complex splitting pattern. Only when considering heteronuclear coupling, was it possible to explain the involvement of H-26a in a ^3^*J*-coupling with the ^14^N nucleus of the isonitrile group. The coupling of the hydrogen with ^14^N, representing a spin-1 nucleus, leads to an additional signal splitting to (pseudo-)triplets with a relative ratio of 1 : 1 : 1. After including the ^14^N spin-particle and its coupling into the spin simulation, a fully matched spectrum was obtained. Moreover, the coupling constant of H-26b with the isonitrile nitrogen could be determined to be as small as 1.13 Hz, which was required to achieve convergence during the HiFSA iteration. It is a reasonable hypothesis that similar evidence is prevalent to other nitrogen-containing small molecules. Access to raw heteronuclear NMR data would much facilitate the analysis of high quality original ^1^H NMR data of N-containing NPs. However, most current databases do not support this kind of data mining, because the stored data is interpreted information rather than raw data. Recently developed repositories such as Protein Chemical Shifts^312^ are no exception. Other typical examples of obscured NMR information is the use of “multiplets” (m) to describe signals with more than two or three spin–spin couplings. This particularly affects signals with small couplings, such as the long range ^1^H–^14^N couplings, which otherwise could help exploit the C–H orientation in three bond anticlinal or synclinal arrangements relative to the lone pair electrons.

Collectively, the above points clearly support the importance of raw NMR data for heteronuclear NMR in the nitrogen domain, encompassing both ^14^N and ^15^N effects.

### 7.7 Phosphorus: ^31^P NMR in natural product structural investigations

The structural nature of phosphorus present in naturally occurring molecules may be identified by ^31^P NMR spectroscopy.^313^–^317^ The ^31^P nucleus being 100% abundant, having a broad chemical shift range of ∼800 ppm, and with a receptivity 377 fold that of ^13^C,^317^ represents a sensitive tool for deducing the structural nature of phosphorus when present. The two main parameters associated with ^31^P NMR that are used for assessing structural content are: (1) ^31^P chemical shift which is typically used to identify the type of phosphorus (*e.g.*, phosphate, phosphonate/phosphonic acid, or phosphinate/phosphinic acid); and (2) ^31^P coupling constants to both ^1^H and ^13^C. The latter information serves to pin down the “local neighborhood” in which the phosphorus-containing structural element resides. The information deduced from the ^1^H/^31^P *J*-couplings, is useful for assessing 2- or 3-bond relationships including configurational criteria, and may be extracted directly from the 1-D ^1^H NMR data with the assistance of HiFSA. Selective heteronuclear decoupling of the ^31^P contribution to the ^1^H spin system would facilitate the HiFSA interpretation of the ^1^H NMR spectrum. Moreover, the use of ^1^H homonuclear-2D-*J*-spectroscopy can also be used as orthogonal NMR methodology where, provided the digital resolution along the F2 dimension is optimized, the F2 projection results in a ^1^H-decoupled ^1^H NMR spectrum but one which retains the ^1^H/^31^P *J*-coupling information. Examination of the fully coupled ^31^P NMR spectrum produced by gated decoupling of ^31^P will also reveal the ^1^H couplings to phosphorus. Since most phosphorus-containing small molecule NPs contain but a single phosphorus atom,^318^–^323^ this represents a very straight forward approach to spectral analysis. The coupling of ^31^P to the ^13^C signals in the molecule further defines the structural environment of the phosphorus substituent and can be evaluated directly from the proton-decoupled ^13^C spectrum. If the NMR spectrometer is so equipped, decoupling of ^31^P from ^13^C can be achieved which unambiguously defines which carbon signals are coupled to ^31^P, including very small couplings which may reside within the linewidth of the carbon signal. Large couplings will be collapsed and removal of small couplings will lead to a sharpening of resonance lines with concurrent reduction in the linewidth-at-half-height (*w*_1/2_). Because of the large difference in the observation frequency between ^13^C and ^31^P nuclei in a molecule with only one phosphorus atom, with broadband ^1^H decoupling, spectral interpretation of the ^31^P coupling to the ^13^C is first order. The coupling of ^31^P to ^13^C can also be deduced from a ^31^P-detected ^31^P,^13^C-HMBC or ^31^P,^13^C-HSQMBC experiment. If, however, there is more than one phosphorus in the molecule and the phosphorus atoms happen to be spin coupled, and have the same or similar chemical shifts, the spin pattern observed for the proton-decoupled ^13^C spectrum arising from coupling to ^31^P will exhibit higher order spin coupling effects (virtual coupling). Examination of ^1^H fully coupled ^31^P NMR spectra can reveal the number and kind of aliphatic groups attached to the phosphorus. For example, the proton-coupled spectrum of the ^31^P signal of trimethyl phosphate exhibits a 10-line pattern arising in this case from spin coupling to nine equivalent protons (*n* + 1). This provides additional structural hooks.

Finally, it should be noted that the relatively high sensitivity of the ^31^P nucleus make it attractive for the establishment of quantitative ^31^P NMR (qPNMR) methods. This enables the determination of impurity profiles with high selectivity, as has been demonstrated for phosphonomycin, **105**, (now fosfomycin), a broad spectrum antibiotic discovered from a *Streptomyces* species in 1969.^324^ It is used parenterally as the sodium salt, or orally as the calcium or more commonly, as the tromethamine (trishydroxymethylaminomethane) salt. In either of the first two cases the only significant degradation product arises from opening the epoxide ring to give a mixture of the (1*S*,2*S* and 1*R*,2*R*) diols **106a** and **106b**, collectively referred to as “impurity A”. The lack of UV absorbance and the high hydrophilicity of these compounds confounds the usual HPLC quality analysis procedures and Jiang *et al.* have developed a quantitative ^31^P NMR (qP NMR) method circumventing these problems.^325^
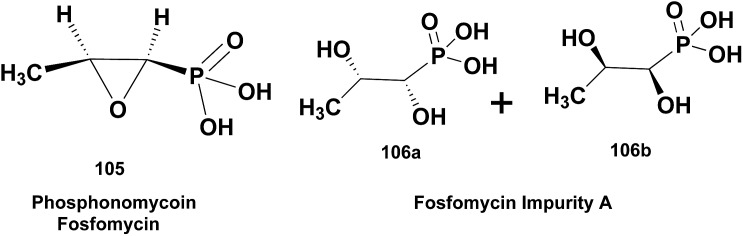


Considering that the majority of investigated NPs are devoid of phosphorus, it is even more important to realize that (selective) ^31^P derivatization and subsequent qP NMR has great potential to advance the analysis of complex NPs. One smart concept targeted at expanding the utility of ^31^P NMR to oxygenated NPs involves the *in situ* labeling of labile hydrogens (aliphatic as well as phenolic and carboxylic hydroxyl groups) with a phosphitylation reagent. Using 2-chloro-4,4,5,5-tetramethyl-1,3,2-dioxaphospholane (Cl-TMDP) as reagent, the proof of concept was demonstrated for the analysis of lignins, which consists of condensed and uncondensed polyphenols.^326^ Recently, this method has been developed further into a simultaneously qualitative and quantitative ^31^P NMR method for the analysis of complex mixtures of condensed tannins (proanthocyanidins, such as **75**) in *Acacia* and *Schinopsis* species.^327^ The method takes advantage of the large ^31^P chemical shift dispersion of the derivatized groups, the structural information from HSQC spectra of the derivatized materials, and the favorable sensitivity and selectivity of qPNMR. Collectively, this allowed the comprehensive characterization of complex proanthocyanidins from crude mixtures, including the quantification without the need for identical calibrants, both of which represent major phytochemical challenges. Considering the chemical complexity of such analytes, the availability of raw ^31^P data will predictably advance the knowledge base for interpretation of qualitative and quantitative ^31^P NMR spectra.

In the context of the raw data focus of the present review, it should finally be pointed out that ^31^P NMR reference spectra have the potential to inform subsequent studies aimed at solution structures of drugs binding to molecular targets. One example is the complex of the antibiotic, nisin, and a shortened version of the bacterial cell wall precursor lipid II (3LII), which show differences in the ^31^P chemical shifts of the free *versus* nisin-bound forms of 3LII, as a result of intramolecular hydrogen bonding.^328^ In the same study, overlaid ^15^N HSQC spectra were also employed to map nisin binding.

## 8 Databases

### 8.1 Database introduction

Both the quality and resolution of NMR spectra have improved since the introduction of the first Varian commercial spectrometer, the HR-30, in 1952. However, most of the contributors to this review never experienced the beginnings of NMR before computer screens replaced the long scrolls of paper sheets that were used. The digitization of NMR did simplify tasks such as peak-picking, integration, phase correction, and apodization functions. It also enables the sharing of spectra over networks.

But this digitization also came with adverse side effects. As every manufacturer developed new features, used different digitization technologies, and different acquisition methods, the complexity of the file formats increased. Current multi-vendor NMR software accommodates up to 20 raw data formats, not counting the different flavors of each of these formats. In addition to these differences, the storage methods are different: some use a single file for the acquisition, others require multiple files, whereas even others require a particular directory structure to be functional. Current conversion solutions often involve juggling between several file formats and software tools in order to obtain input data that is compatible with a given NMR software. Such an elaborate process is detrimental to the integrity of both data and associated metadata, as it implies conversion between different floating point and/or integer encoding schemes and introduces rounding errors. The Holy Grail is software that will convert all of the other formats into readable files without the loss of data. Unfortunately, no such software exists.

As long as a user can access a repository of data, and dig in archives (implying the storage support remains active), and can still open those files, everything should be fine, except when it comes to sharing results. As this typically is done through publications, it opens the question of how NMR results are actually reported. Most frequently, as tables, which just may include HSQC, HMBC, and NOE correlations, but sometimes just as text (listings of chemical shifts, coupling constants, multiplicities, and assignments). Recently, it has become customary to include printouts of spectra as ESI.† As this has been limited to PDF format, these published spectra have been “filtered” through various convoluted conversion processes such as screen captures, lossy bitmap compression and/or presentation software, and other operations that involve format changes and/or are associated with degradation of information. The result is often a small, highly pixelated bitmap picture, which hides the details needed to examine a proposed structure (coupling constants, satellites, purity). Beyond data sharing, lurks the problem of the minimal information required to elucidate and describe unambiguously a structure. While publication platforms differ in their requirements, NMR users would benefit greatly from initiatives similar to those developed in the Mibbi project,^329^ aimed at producing minimal reporting guidelines for the biological and biomedical investigations.

Recently, pharmaceutical companies, manufacturers, software companies, universities, and others joined the Allotrope foundation effort (allotrope.org). The foundation's objective is to develop a single universal data format, linking the three main scientific productions: raw data, results and evidence. The single file using the Allotrope Data Format (ADF), contains the original data file, and the treated data (in a standardized but still evolving form). Further, it provides a way for the equipment, people, processes, geographical locations, and projects to be linked and described. The foundation develops standardized vocabularies and data descriptions. With the use of ontologies and semantic web technologies, all of these elements can be linked to other resources (online databases or internal repositories) and annotated appropriately. This joint effort aims to unify current analytical data, including NMR data, and allow NMR records to survive the test of time, crashing hard drives, and the confusion of a myriad of formats.

### 8.2 The urgent need for spectral repositories and automation support for peer-reviewing of spectral data

Structure elucidation of organic compounds starts with experimental data, transformed into spectra, and translation into present/absent structural fragments. The final step combines these fragments into a structure proposal fitting the given constraints derived from the experimental data. This process can fail and lead to a wrong structure, because this sequence can be broken at any point.^19^,^121^,^330^,^331^ In order to detect wrong conclusions, it is often necessary to trace back to the raw data.

Furthermore, there is a major need for validated reference materials of authenticated chemical structures in order to build spectral databases that can fully support the process of upcoming structure elucidation problems. As long as the scientific community relies on non-validated reference materials with potentially wrong structures, conclusions derived remain uncertain. The unwanted consequence of this domino effect is that the impact of non-validated results increases, rather than decreases, by contributing to an increasing number of potentially wrong structure proposals.

The following two case studies exemplify this tight relationship between research quality and the availability of raw data: (i) aglalactone isolated from *Aglaia elaeagnoiea*, and (ii) the identical NMR-data published for orientanol A and eryvarin A. The wrong structure proposal for aglalactone, (**35**)^118^ has been revised to **36** in a subsequent paper.^122^ The following situation has not been corrected. Orientanol A (CAS-RN: 190381-82-9; C_21_H_24_O_7_, **107**)^332^ was published by Tanaka *et al.* for the first time having a 2,3-dihydroxy-3-methylbutyl side chain showing ^13^C chemical shift values of 27.1, 78.6, 72.9, 26.1 and 25.0 ppm. In a later paper^333^ published by the same group, a new isoflavonoid named eryvarin A (CAS-RN: 302928-70-7, C_21_H_22_O_6,_**108**)^333^ was described having nearly identical carbon chemical shift values (9 positions differing by 0.1 ppm each). The ^1^H chemical shift values are also identical within 0.02 ppm; and the coupling constants are within the range of the digital resolution of a standard ^1^H NMR experiment. It is interesting to note that even the labile hydrogens of both OH-groups in **108**, are identical to those in **107**. Formally, **108** is created from **107** by cyclization of the side-chain and elimination of H_2_O. Despite this cyclization, the chemical shift values of the carbons and hydrogens in the sidechain of **107**, which is now converted into a six-membered dihydropyran ring system in **108**, remain unchanged. Table 5 compares the ^13^C NMR experimental values of the five carbons within the sidechain against their expectation ranges and the expectation ranges of the 2 possible cyclization products. Table 5 also shows that the experimentally determined chemical shift values fit best to the sidechain product named orientanol A (**107**), whereas the dihydropyran-derivative **108**, is a reasonable alternative structure to the given data under the assumption that the resonances at 78.6 and 72.9 ppm have been misassigned despite measuring 2D NMR-spectra. The spectral data of eryvarin A have been repeated (compound **7** in ref. 334). There is no claim that either orientanol A or eryvarin A is the correct structure proposal to this set of spectral data, but the severe inconsistency in the underlying data material is clearly visible. It is also clear (Table 5) that the alternative ring closure structure (**109**) is not viable.
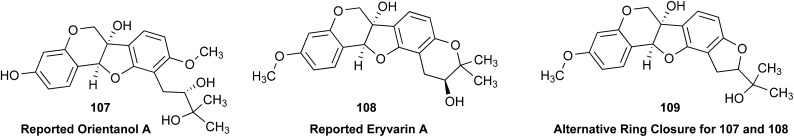


**Table 5:** Experimental and predicted ^13^C NMR data for possible structures of orientanol and eryvarin. Either orientanol A (**107**) or eryvarin A (**108**) (with an assumed assignment error) seem to be reasonable structure proposals to the given ^13^C NMR data, because all five signals are located within or near their expectation ranges, whereas the possible dihydrofuran cyclization product, **109**, shows large deviations. Experimental values within ±1.5 ppm of the expected range: green cell, ±3 ppm: yellow cell; more than ±3 ppm: red cell

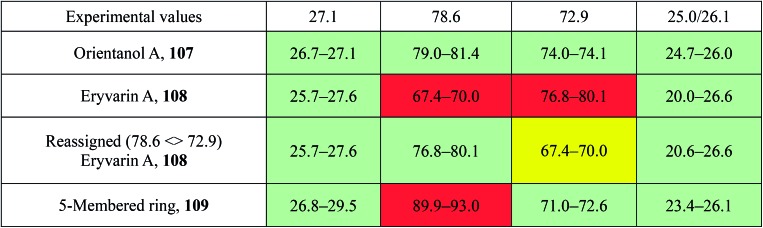

This example demonstrates the urgent need to deposit raw spectral data in an electronic format in a repository in order to reinvestigate the whole process of structure elucidation starting at the very beginning and allowing chemists to follow the whole chain of decisions. It is also clear that there is not always an absolute solution to the interpretation from any one source of data. However, the availability of raw data offers a means of clarifying such a discrepancy.

The CSEARCH-database (nmrpredict.orc.univie.ac.at/) consists of some 700 000 ^13^C-NMR spectra and a sophisticated software-package. The examples given here have been found, when searching for identical spectra published by at least one common author occurring in different literature citations associated with different structures.

### 8.3 Databases for dereplication

The number of known NPs has grown, and continues to grow at a remarkable rate, swelled by over a century of research into terrestrial and marine plants, animals and microbes. A significant proportion of these NPs were first described in the scientific literature prior to, or in the formative years of NMR-enabled structure elucidation. Notwithstanding the many impressive feats of structure analysis achieved in those early years, where available, published accounts of NMR data from this era predating modern high field and 2D NMR spectroscopy, are limited to tabulation of ^1^H NMR (and later, possibly ^13^C NMR) resonances. By necessity, NMR assignments were often either incomplete or tentative, with some resonances described merely as broad singlets or unresolved multiplets, and overlapping resonances collectively listed as “envelopes”, or not listed at all. In an effort to enhance NMR characterization, early authors sometimes published accompanying unannotated images of NMR spectra, although these rarely offered added insights into molecular structure. The limited NMR characterization of legacy known NPs remains an enduring problem to this day, especially as current and future researchers seek to re-investigate these structures, as a prelude to exploring their chemical and biological properties. For example, modern researchers typically re-isolate a known natural product, and use this as a launchpad to explore a rare and unusual structure class. The justification for such an investment is based on the view that known NPs represent a legacy resource of considerable value, capable of accelerating the discovery and development of new molecular products (*e.g.*, as pharmaceuticals and agrochemicals).

Illustrative of just such an investment, during a search for new antifungals the Capon group recently isolated a large and structurally complex (C_56_H_102_N_3_O_15_) natural product from cultivation of a sheep-feces-derived Streptomycete. Based on a preliminary spectroscopic analysis they determined that this metabolite was most likely the guanidyl polyketide macrolide, amycin B (**110**), first reported last century from a Greek soil-derived *Streptomyces* spp.^335^ The amycins are a remarkable class of natural product that include niphimycin/scopafungin,^336^ copiamycin,^337^ the azalomycins^338^,^339^ and guanidylfungins,^340^ neocopiamycin A,^341^ malolactomycin A,^342^ RP 63834,^343^ the shurimycins,^344^ RS-22s,^345^ the kanchanamycins,^346^ and the primycins.^346^ Despite being known as NP antifungals for over 50 years, chemical knowledge of amycin B (**110**), and other members of this structural class remains limited to planar structures, supported by modestly annotated and tabulated 1D NMR data.
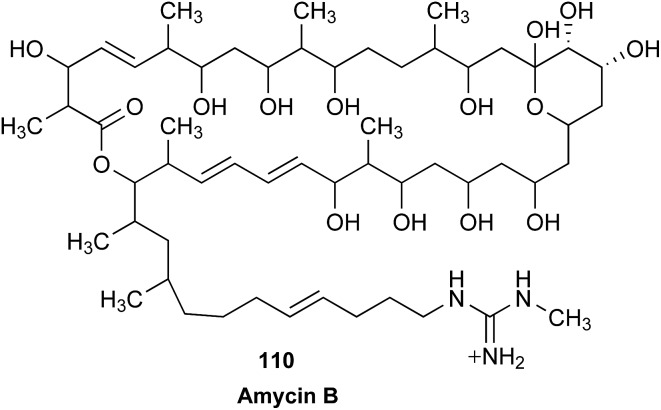


**Fig. 37:**
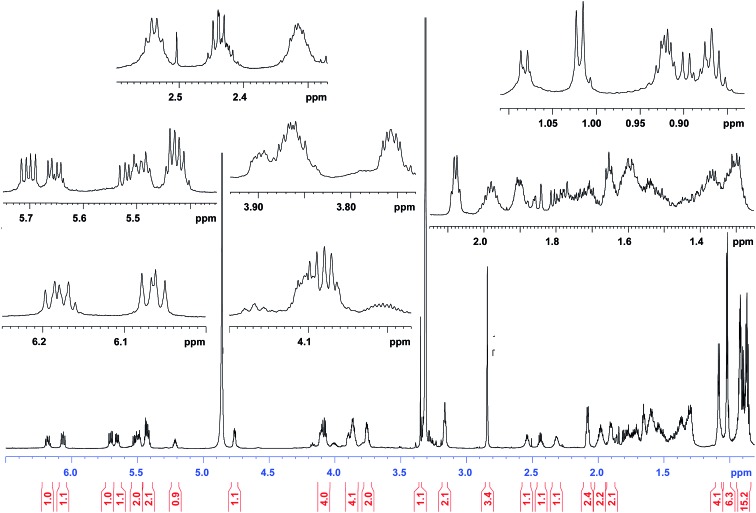
^1^H NMR (900 MHz, methanol-*d*_4_) spectrum for amycin B (**107**).

This is not an uncommon occurrence. There is without doubt a great deal unknown about a great many known NPs. To explore the antifungal potential of amycin B and related NPs it was first necessary to confirm (and if possible complete) existing structure assignments. Whereas the 1D and 2D NMR data acquired on a re-isolated sample of **110**, was an excellent first step (Fig. 37), lack of access to comparable data for other members of this structure class severely limited the scope of these investigations. This dilemma is compounded by the fact that the original authentic samples of these and most other known NPs are generally lost, and commercial sources are largely non-existent (Fig. 37).

A possible solution to this problem lies in the observation that modern NP researchers routinely detect, isolate, characterize and identify known NPs, and in doing so acquire and analyze high quality NMR data, often vastly superior to published data (as evidenced by the reisolation of **110**). However, as the constraints of modern scientific publishing preclude the reporting of known NPs, this NMR data languishes as unpublishable output in the archives of individual laboratories, companies and institutions, albeit a very valuable resource. With modern NMR data comprising electronic files that are readily shared, processed, and analyzed by any number of free and commercial softwares, there is a very strong case for establishing a global NP NMR data repository. This repository could accept, register, curate and facilitate free worldwide access. In due course, scientific journals could make uploading and registering of NMR data a condition of manuscript submission, much as is already the case for X-ray crystallographic and genetic sequence data. The same could apply to (post)graduate NP research theses, which are typically rich in such data. In this scenario, researchers uploading data could be acknowledged on a per data set basis, the registered entry could be cited by future researchers, thereby forstering a collegial culture of international, interdisciplinary, and intergenerational recognition.

### 8.4 The importance of raw data in databases

This Raw Data Initiative is a continuation of previous efforts^1^ to initiate the creation of a repository for raw NMR data dedicated to small molecules, especially those of biological origin. One major goal is to minimize the time taken to identify known molecules and therefore to avoid the duplication of structure determination efforts. Current practice involves extraction, isolation/purification, time-domain NMR data acquisition, conversion of these into the frequency domain, extraction of spectral parameters (chemical shifts and coupling constants) and proposal of a chemical structure by deductive reasoning. A search of available databases provides a high probability that the compound is novel or known. Even in the latter case the task is not finished until a search of the literature confirms that the spectral data of the isolated compound matches that of the known. The preservation of FIDs opens the doors for re-processing with other parameters and/or algorithms, which subsequently allows extraction of more or better spectral parameters from the same data. The same reasoning should encourage the community to constitute reference samples repositories, thus permitting the recording of new raw data sets by means of emerging methodologies. Non-Fourier transformation of FIDs are pertinent in many situations, especially for multi-dimensional NMR data processing, in contexts such as non-uniform sampling, covariance analysis or the extraction of relaxation and diffusion parameters. Even transformed data in the direct dimension may benefit from processing methods like reference line deconvolution. The possibility of reprocessing time domain data therefore warrants the possibility of obtaining better spectral data, better estimations of spectral parameters and finally more reliable structure determinations.^347^

The Institute of Molecular Chemistry of Reims, France, is putting together a focused library of raw, time domain NMR data and transformed data that is linked with enhanced structure files, *i.e.*, Structure Data Format (SDF) files in which atoms may be arbitrarily tagged. These tags are connected to chemical shifts values and paired to coupling constant values. Data was obtained from a small library of glucosinolates and of their desulfated derivatives using the PERCH software,^62^,^348^–^352^ in a process that is similar to the constitution of the MetIDB database. Such a protocol is the only one that ensures a realistic transposition of a 1D ^1^H NMR data from one static field value to another for comparison purposes.

The same research group has also designed a dereplication workflow based on ^13^C NMR data and is used for the analysis of complex plant extracts.^353^ The ^13^C NMR spectra of the samples produced by Centrifugal Partition Chromatography fractionation are binned and the bin contents are classified according to the resemblance of their chromatographic profiles. Sets of chemical shifts with similar profiles constitute keys to the search for known compounds in a locally developed and enriched database that links structures and ^13^C NMR chemical shifts. The latter are obtained by prediction from structures by means of commercial software. The availability of raw NMR data reference compounds would contribute immensely to the progression of efficient dereplication tools.

### 8.5 The breadth of databases and their use by chemists

The past 10 years have witnessed the development of a global network for data sharing of spectroscopic and chromatographic data in metabolomics.^354^,^355^ The principles behind this data sharing network have been established over the past 20 years by the molecular biology community in areas such as genomics and proteomics.^356^ Since the field of metabolomics is populated by a considerable number of analytical chemists there is now hope that the principles of free and open data sharing will be more widely adopted by the quite conservative chemistry community. This hope is supported by initiatives like the FAIR data movement,^357^ which advocates for research data being Findable, Accessible, Interoperable, and Reusable (FAIR), in order to properly support the scientific methods. These are gradually being adopted by major funding agencies such as the European Commission, the National Science Foundation, the National Institutes of Health and many other funding organizations. The fundamentals of metabolomics data sharing are not very different from those in NP chemistry. Data in Metabolites and other databases are composed of a core of raw data surrounded by metadata aiding in the interpretation of the experiment. Whereas the raw data consists of NMR, MS, and chromatographic data, the metadata covers the whole range necessary to understand how the study was conducted: the species under investigation, which organism part the metabolite was isolated from, which instruments were used, and which parameters were used.

Every data point in the above-mentioned metadata is backed by a term in a commonly used ontology, and the question which type of metadata needs to be reported is dictated by the minimum information standard. For metabolomics these minimal information (MI) standards were created by the metabolomics standards initiative around the year 2007.^358^ Most of the minimum information principles established by the MSI are directly applicable to the NP community. Additionally, information from the 2015 initiative to establish the minimum information about a biosynthetic gene cluster are relevant.^359^ The molecular biology community, in establishing databases like MetaboLights and the metabolomics workbench, has laid the technological foundation for the archives necessary to establish a raw data NMR sharing in NP chemistry. MetaboLights, for example is completely based on open source technology, open data standards and open data formats, and community-based reviews on the topic have appeared.^360^

Following the establishment of MetaboLights, the COSMOS initiative^361^ has complemented the set of open data formats for mass spectrometry such as mzML with a sister format for the representation of raw NMR data, nmrML, which has been established very recently.^362^ With clear signals by the major NMR instrument manufacturers for the support of this new open format, nmrML has the power to replace the age-old JCAMP as a usable open data format in NMR spectroscopy. The computational frameworks to hold and describe raw data and metadata are equally in place. The ISA format, for example, is widely used across domains in molecular biology and metabolomics in particular.^363^ It is capable of holding all the necessary metadata of an investigation, study and its underlying assays (ISA = Investigation, Study, Assay), in a spreadsheet-like format, backed by a wide range of ontologies, including the NMR term ontology established as part of the nmrML work.

There are indeed few differences in describing an NMR-based metabolomics experiment and describing the isolation and identification of a NP, the most important of which might be the fact that the latter is hopefully based not on mixtures but spectra of pure compounds. In summary, it is anticipated that this work, embedded in a large, worldwide community interested in metabolomics data management over the past 10 years will be instrumental in establishing a network and movement for NMR data sharing in NP chemistry. Several publishers have already embraced open data sharing for the articles published in their journals, often at additional burden for the researcher, and dedicated data publications^364^ in certain journals are a viable alternative to the typical reports about the isolation of NPs found in more traditional outlets.

### 8.6 Raw NMR data formats

The recently introduced mzML, nmrML, sibling formats for MS and NMR, respectively, aim at integrating two analytical techniques that are essential for metabolomic analysis. The utility of NMR and the importance of open data formats in advancing the contextualization of metabolomic data in pharmacognosy research has been highlighted by some of the authors.^365^ While well-intended at the time of implementation, existing implementations of the IUPAC JCAMP-DX spectroscopy standard into NMR software vary in adherence to the standards and inter-operability. In practice, JCAMP data are not fully compatible between the various software platforms. More comprehensive data standards exists, such as the ISA^363^ and ADF (; allotrope.org) formats that are fully capable of including NMR metadata and even provides the ontology of nmrML, but their broader implementation is pending and complicated by their broader scope.

One very recent effort approaches the standardization of NMR data format for instruments and software through a process driven by an extensive consortium of manufacturers of analytical equipment and its user base, representing a variety of fields in research and application: the Allotrope Foundation (allotrope.org) has developed a universal raw data format, the Allotrope Data Format (ADF), which also accomodates the storage of derived results. Another effort, co-led by one of the present authors,^366^ is the NMReDATA initiative (; nmredata.org)^367^ and combines forces from the NMR scientific community consisting of individuals, software manufacturers, and the journal, Magnetic Resonance in Chemistry (MRC). One tangible recent result is that MRC requires the dissemination of digital NMR spectra and data for assignment articles submitted since early 2018. Moving forward, the MRC editors also intend to require that authors supply raw NMR data as a means of result verification.^368^

The establishment and widespread implementation of universal data formats in science is a major challenge. Creation of the actual formats and their acceptance are both evolutionary processes, which can be predicted to take time. In fact, progress may depend more heavily on the success of consensus building mechanisms than on the scientific mechanics of the actual format definition, for which the above initiatives have already paved the way. As this process continues to unfold, it is important to realize that the data produced by NMR instruments already represent a “native” form of raw NMR data and are readily available for use. One key message of this Raw Data Initiative is that there is no reason for procrastination. Archiving and dissemination of raw instrument data is feasible and practiced by an increasing number of scientists. Albeit somewhat proprietary, the single FID/SER files and pre-defined folder structures can be read by many software tools, even when produced by older hardware, and transcription to the future raw data standard(s) will almost certainly be straightforward *via* automated conversion tools.

## 9 Clinical uses

### 9.1 Expanding raw data concepts from chemistry to clinics: moving from NMR to MRS

Thirty years ago, the idea of bringing NMR into a clinical setting using magnetic resonance spectroscopy (MRS) generated considerable excitement. Despite this enthusiasm, MRS is still not widely used, in part due to financial considerations (billing for time on scanners), lack of expertise in radiology departments, vendor reluctance to develop new software, and possibly foremost, because the number of metabolites identifiable is small. Today, the main use for MRS is to distinguish a brain lesion as a tumor or non-tumor. The most common metabolite signals used to identify a tumor are a decreased *N*-acetylaspartate/choline and increased lactate-to-lipid ratios.

Reflecting the belief that the power of MRS for chemical imaging of the brain and other organs is enormous, raw MRS data is being revisited with machine learning and other mathematical tools, beginning with a large database of pediatric brain tumors. Starting with this particular database is important as recent technical improvements in MRS allow the identification of other tumor-specific markers that can help classify tumor biochemistry,^364^,^369^–^372^ and by progress in applying new analytical tools to increase the number of identifiable signals. Using newly developed normalization and other mathematical tools,^373^ proof of concept has been generated for the identification of >90 signals from brain MRS data of pediatric concussion subjects.^374^ The MRS fingerprints enabled differentiation of healthy children from those with concussions. The tools were applied to processed, post-FT data, neglecting the imaginary numbers in the data, so that pre-processed data represents a largely untapped source of information. While optimization of the data analysis tools is work in progress, the results already suggest the value of revisiting raw MRS data, which are often not stored and, thus, lost.

Current approaches to MRS data analysis carry assumptions about which chemicals contribute to an *in vivo* spectrum. However, these are incomplete or even flawed. Importantly, they diminish the capability of detecting metabolic features that are not inserted *a priori* in the underlying MRS models. Future work will group patients into clinically relevant subgroups (responders *vs.* non-responders to certain therapies) and look for common chemical signals, thereby bypassing any assumptions. If successful in the long-term, this research will provide readily obtainable (noninvasive 30 min scan on any state-of-the-art MR scanner) metabolic signatures at the time of diagnosis that lead to personalized therapy.

Collectively, the availability of raw MRS data is crucial for the ability to extract new insights from existing measurements that are performed daily, on a routine basis. Similar to NMR in chemical analysis, raw MRS data contain a plethora of untapped information, which can be unraveled. Notably, because NMR and MRS share the same underlying nuclear resonance mechanisms, insights derived from chemical NMR analysis could potentially inform clinical MRS applications, and *vice versa*. Similar prospects for the utility of raw NMR data disseminated *via* an open database concern other forms of *in vivo* NMR spectroscopy, including a 1D ^1^H or ^31^P experiments aimed at the chemical analysis of tumor and other pathological tissues. The ability to quantitatively assess contributions from certain identified metabolites can provides valuable information for subsequent patient treatment and open opportunities for individualized medicine.

## 10 Conclusions & outlook

### 10.1 Decades of manual mining prove the concept

Comparing the development of mass spectrometry (MS) and NMR spectroscopy in terms of databases and computational tools clearly indicates that data simplicity has been the main driver of the use of such data: both the ions in MS and the singlets of ^1^H broad band decoupled ^13^C NMR data can be represented as *x*,*y*-pairs (MW or chemical shift, respectively, and intensity) and, thus, can readily be transformed into search algorithms. As coupling or other connectivity/spatial relationship information is invovled in nearly all other 1D and 2D, NMR spectra are inherently more complex and mostly evade a simplistic treatment.

However, the development of the CSEARCH database (http://nmrpredict.orc.univie.ac.at/) for the systematic mining and use of ^13^C NMR data can serve as an excellent example of the information content of NMR spectra in general. Especially for ^13^C NMR, the chemical shift value of a given carbon atom is highly characteristic of its chemical environment in a given molecule. In fact, deviations are so small (in the low ppb range) that even the absolute configuration of monomeric building block in oligomeric compounds can be achieved^183^,^184^ and subtle differences in the diastereoisomerism of closely related congeners can be recognized.^60^ Importantly, for general applications, ^13^C NMR enables structural dereplication with extremely high degree of certainty, provided that adequate acquisition conditions are employed to ensure comparability of the data sets (*e.g.*, concentration range, solvent, temperature). The CSEARCH database clearly highlights both, the dereplication of ^13^C NMR and the necessity to make NMR raw data accessible to the scientific public. The database has been built over decades by transferring tens of thousands of assigned NMR data sets in combination with the structures derived, from peer reviewed journal sources into a digital format. From this starting point, data comparison, and shift value statistics and shift value-structure motif correlations were made possible. Taken together these two contributions, which do cover more than a decade of scientific progress, prove that improvements in NMR data handling, data interpretation and data presentation are still needed. It must not be overlooked, that the mere presentation of processed NMR spectra in the ESI,† as advocated by many scientific journals, was only a first step forward. The shortcoming of printed ESI† has been a subject of discussion in other scientific communities, such as genetics.^375^ It does not sufficiently address the problem, since both spectral overlap and low resolution graphics usually allow no unequivocal analysis of spectral identity or special spectral features. Raw NMR data, especially for 2D NMR spectra, are usually a few megabytes only and even desktop-grade IT infrastructure will allow its swift dissemination. The “soft revolution” in NMR technologies allows the processing of such data independent of the instrument platform involved in their recording. Hence, raw NMR data deposition is needed urgently, for the following four key reasons. (i) It is vital to present raw NMR data of substances isolated from natural sources or synthesized in a total synthesis approach aiming to verify a structure hypothesis. This obligation is especially important if a new NP discovery claim is made. (ii) It is equally important to present raw NMR data of substances/substance mixtures administered in pharmacological *in vivo* and *in vitro* studies; especially if isolates from natural sources are investigated. Only if certified material (with NMR data) is utilized can this obligation be waived. (iii) Raw NMR data should also be made available for substances/substance mixtures that are utilized as calibrants in quantitative measurement campaigns; especially if isolates from natural sources are investigated. Only if certified material (with NMR data) is utilized this obligation can be waived. Finally, (iv) industry, especially outfits that bring measurement platforms to clinical use (under FDA or IVD-CE clearance), monitor drug (metabolites) and/or raw materials used for calibrant production by NMR spectroscopy should always provide the respective raw data.

### 10.2 The urgent need for public dissemination of raw NMR data

**Fig. 38:**
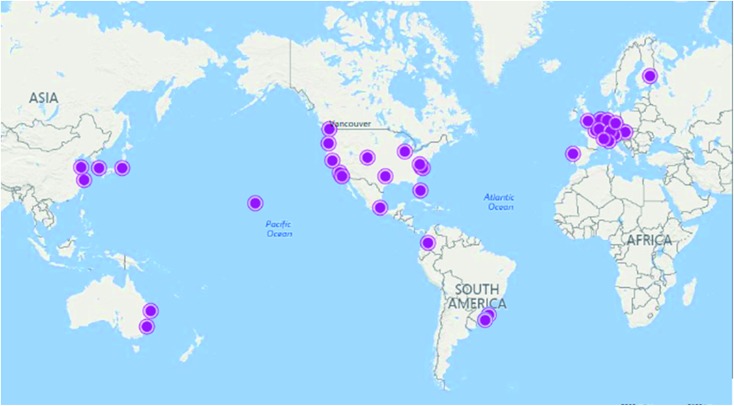
Support for the call for disseminating raw NMR data comes from the global natural product research community, as shown by the locations of the authors who contributed to the present study.

This review aims to increase the awareness of NP scientists, and the entire chemistry community, to the advantages of modern software tools to extract meaningful data from raw FIDs and avoid the loss of information in the process of NMR data interpretation and documentation. This urgent call for a change in scientific dissemination practices is supported by a global group of scientists, who contributed to the present review (Fig. 38). Specifically, this group advocates universal depositing of these FIDs in free access databases, institutional repositories, or at least in investigator-initiated ESI† files so that the FIDs will be available to scientists worldwide, not the least to manuscript reviewers. Establishing the public sharing of raw NMR data as a standard practice will create both obligations and opportunities that engage authors, reviewers, and readers equally. This creates a mutually beneficial push–pull relationships that can only enhance the integrity of science and reduce the occurrence of incorrect published structures,^19^,^22^,^23^ especially when considering that researchers essentially serve in all three roles simultaneous, *i.e.*, as authors, reviewers, and readers.

Although a number of databases exist, there is no universally accepted format, especially for crucial FID-associated metadata, such as, solvent, temperature, concentration, instrument, field strength, and charge (*i.e.*, pH or more likely pD) for spectra of compounds with ionizable groups. This review reports on at least two examples where conclusions have not yet been reached because spectra of the same (or not) compound have not been identical, almost certainly because spectra were taken of samples with different degrees of ionization. In general, this is particularly a problem with peptides. Fortunately, the reporting standardization including metadata aspects may be addressed as IUPAC has put together a Project Task Force to address just this problem. A global, universally accepted database is an enormous task. Its feasibility will depend on an adequate combination of international coordination, funding, and sustainable mechanisms, most likely required by “first world” countries. Historically, funding and sustainability have restricted most existing databases. The rise of distributed databases, linked data, and data-interoperability consortia could provide alternative monolithic data-silos that are difficult to maintain. In fact, these approaches are more likely to ensure viability, accessibility, and achievement of overall project scope for a global, universally accepted database containing raw NMR data. The availability of metadata for raw NMR data (FIDs) becomes even more crucial for experiments that involve randomly generated parameters, such as the randomized t_1_ sampling schemes in non-uniform sampling (NUS) 2D NMR experiments. Collectively, metadata is a vital part of the raw NMR data and responsible for making data sets fully transparent.

The case is made here that ^1^H NMR data alone, if mined thoroughly, can go a long way to overcoming most of our current problems. Correct structure elucidation is an absolute necessity for the development of bioactive leads. The prevailing mantra is that absolute structure determination is only achieved through X-ray diffraction or total synthesis. The former can have difficulty in distinguishing O and NH and the latter is both resource intensive and not infallible, as is evidenced by the interesting case of elisabethin A, isolated in 1998 from a West Indian Sea Whip and assigned the structure **111**.^376^
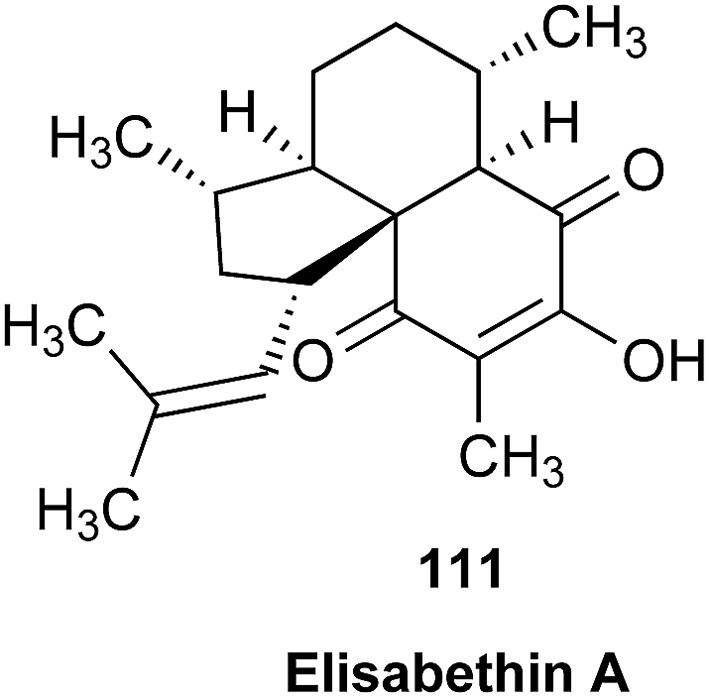


In 2004, the total synthesis was claimed,^377^ to be contradicted almost immediately,^378^ as beautifully summed by David Whitehead at ; www2.chemistry.msu.edu/courses/CEM958/FS04_SS05/whitehead.pdf. The structure of elisabethin A could well be that claimed by the original authors, but it has not yet been confirmed by synthesis. It seems likely that the synthetic product is a diastereomer of **111**. This case supports the claim that there is no such thing as absolute structure proof. Nonetheless careful analysis of all of the data embodied in a simple, but accurate ^1^H NMR spectrum can lead to a structure, in which there can be high confidence, even when the spectrum has been acquired on microgram quantities of highly pure material.

The availability of raw NMR data would also serve as a catalyst for the increasing number of studies that utilize quantum chemical calculations for the purpose of structure elucidation. The computation of chemical shifts and coupling constants using quantum chemistry is now regularly included as a key component in the assignment and revision of the structures of complex NPs. While theoreticians have been developing methods for computing these values for decades, many organic chemists first became aware of the power of such approaches through Rychnovsky's reassignment of the structure of hexacyclinol (**112** to **113**).^379^ A variety of reviews have compiled examples (there are many) of similar studies,^380^–^382^ and Hoye and co-workers have even provided a tutorial for carrying out such studies.^383^ Several representative examples have been discussed in this review. The combination of experimental data and quantum chemical calculations has the potential to revolutionize structure determination, both its speed and accuracy.
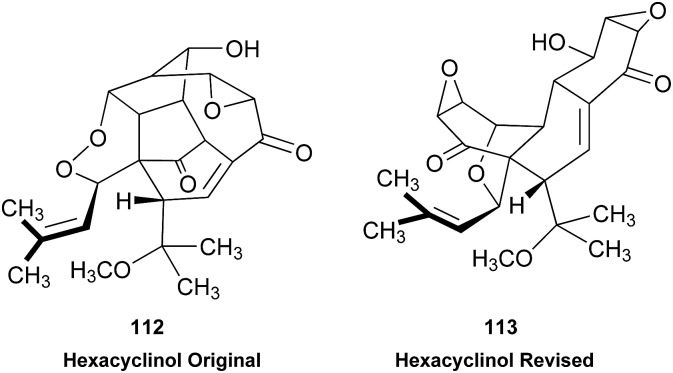


### 10.3 Evolution of raw NMR data repositories

The term “database” is used frequently to refer to gathered digital content of any kind, stored in a binary or text format, using a more or less pre-defined structure, *e.g.*, that of a relational or NoSQL database. For the purpose of this discussion, the “container” is defined as a database, the content as (raw NMR) data, and the combination of the two as a repository. Moreover, as the term “data” has a very broad meaning, in the present review, data refers to the combination of experimental data (*i.e.*, the information/data obtained during the NMR experiment, in particular the FID) as well as the metadata that are necessary to reproduce the experiment (*e.g.*, field strength, pulse sequence, solvent, concentration, temperature, *etc.*). Often, additional information is important or helpful, but not strictly necessary to reproduce an experiment. Such data can be referred to as ESI† and encompasses, *e.g.*, patient information from the clinical trial that produced the NMR samples that enables statistical analysis.

The establishment of a (raw NMR data) repository encompasses two principal steps: (i) definition of the information that is intended to be stored, including which experimental and ESI† is required and/or optional; (ii) conception, structure, and IT aspects of the repository itself. Both choices are critical as they have implications for the maintenance and evolution of a repository, especially when it is intended for long-term service. Migration of information from one database (container) to another is typically possible, with effort depending on the database technology. Despite this basic flexibility, it is not possible to recover information that has not been stored to start with. While this may sound trivial, it highlights in fact key points of the present article: (a) diligence and inclusion are paramount; (b) data which has not been stored in the past –as is the case with the majority of the experimental NMR spectra acquired since the inception of FT-NMR – is irrecoverable; and, therefore, (c) building of such a repository is a timely and urgent task.

Another conclusion from the general portability of a database is that, as long as the stored information is in definite format and structure, annotated, and accessible, the container itself is irrelevant to the data. However, the container is most relevant for the users as it is what scientists are interacting with. The availability of modular and publicly accessible APIs is mandatory to make the data meaningful. Under these conditions, the development of the repository, its data structure, and its storage technology can be handled separately, as long as the scope of each aspect has been defined. This will ensure that the scientific community, including NP researchers, can build the tools that can be integrated into the repository and are best suited for the particular needs of an application.

Some of the essential properties of a global repository are that it (i) provides the user with the ability to upload information and obtain a unique and permanent identifier (such as a DOI [Digital Object Identifier]) that points to it; (ii) ensures efficient access to the data, *e.g.*, *via* batch downloading and programmable interfaces (APIs); (ii) guarantees the long-term availability of the data. The last point has been addressed very recently by the Organisation for Economic Co-operation and Development (OECD), which has developed recommendations for sustainable business models that balance policy regulation and incentives and can assist researchers, policy makers, and funders involved in repositories.^384^ A global repository should be able to deliver a permanent identifier, such as a DOI, for each deposited object including the NMR experiment, the relevant molecule(s), assignments, linked publications, *etc.* Any objects based on information that are already stored in the repository can be used to generate a permanent hyperlink-like structure that connects, *e.g.*, assignment to the associated spectra and publications to assignments.

In addition to these fundamental functions, and depending on the particular research area, databases may offer more “intelligent” functionality to the repository, such as advanced browsing or interfaces for novice users. The NP community has a high demand for tools for dereplication and identification, including separation, isolation, structure elucidation, and metabolic profiling. As such tools evolve according to community needs and will most likely remain under permanent development, it is necessary to separate their design and maintenance from the construction of the repository. At the same time, the repository should foster an enabling environment for projects that advance NP research.

As the use of software tools in NMR analysis is becoming increasingly critical, all data resulting from software output should also include a permanent hyperlink that points to the version and ideally to the underlying code that produced the output. For instance, almost all FIDs recorded today are subject to digital filtering, and an error in such a central component of the software/hardware workflow could have confounding consequences, especially if the underlying algorithm is undocumented. This again emphasizes the importance of storing NMR data in an as unmodified form as possible (“raw”), similar to what is customary in digital photography. Depositing original, raw NMR data and obtaining a unique identifier for them is also the most straightforward approach.

Major efforts towards the development of repositories for raw NMR data have already been expanded. The following list compiles several of them, in no particular order: NMrb was launched in 2004 as a repository of raw NMR spectra for biosciences,^385^ and apparently has disappeared; SPECTRa (; https://spectradspace.lib.imperial.ac.uk:8443/handle/10042/25) was a project for the sharing of raw NMR data, but is inactive since 2008; NMRShiftDB (; http://nmrshiftdb.org); SDBSWeb of the National Institute of Advanced Industrial Science and Technology, Japan (; http://sdbs.db.aist.go.jp) allows downloading of peak-picked data, assignments, and bitmap images; Chemspider (; www.chemspider.com) is a free but not open database of chemical compounds that provides NMR raw files as subsidiary data for a limited number of compounds; The Human Metabolome Database (; http://www.hmdb.ca/)^386^ focuses on human metabolites and contains raw NMR data for selected compounds; Biological Magnetic Resonance Data Bank (; http://www.bmrb.wisc.edu/)^387^ seeks to provide qualitative and quantitative NMR data (processed, assigned; not raw) of biological macromolecules and metabolites; the Open Spectral Data Base (; http://osdb.info/)^388^ is an open source project intended to be extended, enhanced, and used for open science data sharing by its users; ; C6H6.org is an open source project, built using recent technologies and running inside a web browser, offers means to store, share, analyze, and interact with raw NMR data.

### 10.4 Action items for implementation

A very recent initiative by the National Center for Complementary and Integrative Health (NCCIH/NIH) has solicited information from the research community regarding the development of an open-access NMR data repository (NOT-AT-17-015 at https://grants.nih.gov/grants/guide/notice-files/NOT-AT-17-015.html). Recognizing the repository gap for NMR data and the particular importance of NMR as an analytical technique for NP research, the initiative has been seeking input on a comprehensive list of topics which exemplify the breadth of parameters: purity standards for single chemical entities, value of spectra from complex mixtures and nuclei other than ^1^H and ^13^C; nomenclature (spectra, structures) and other key data standards; minimum metadata requirements; harmonization of publication standards; association with other analytical such as LC, UV, and MS; analytical tools required to achieve the most value; minimum size and diversity for maximum usefulness; key features and functionality from different perspectives including users, data contributors, and/or the research community.

Considering the overwhelming evidence of the cases presented in this review for urgent need for raw NMR data, an improvement of the situation can be achieved by taking action at several different levels, as follows:

#### 10.4.1 Organized data storage

At the very minimum, all generated NMR data that has been identified as being valid and/or is essential for a given project should be placed into a secure storage, with backup, so it can be retrieved later, *e.g.*, for deposition in a repository. Considering the immense value of raw data for originally unintended and confirmatory (meta) analysis, systematic data storage organized at the level of research groups, centers, or organizations strongly supports what the scientific community and funding agencies increasingly acknowledge as “good laboratory and research practices”.

#### 10.4.2 Active dissemination and publication

Current publication mechanisms, in particular classical journal and book publications, should implement or at least actively support the active dissemination of raw NMR data, along with every published article and book. Naturally, authors should retain ownership of their data. Already existing means of internet-based mass dissemination enabled global dissemination by individual, but coordination of individual activities and sustainability remain as challenges.

Independently from, or in parallel to, classical publications outlets, authors can take immediate action by depositing raw NMR data into publicly accessible repositories. Institutional (*e.g.*, university and research institution based) and global (*e.g.*, Harvard Dataverse; ; dataverse.harvard.edu) solutions exist already for this purpose and offer sufficient flexibility to share raw NMR data today, while allowing for their inclusion into a global repository envisioned for the future.

#### 10.4.3 Unified global repository

Envisioned collectively by all authors of this review, the ultimate action item is the implementation of a global, ideally unified repository for raw NMR data. In its ideal implementation, such a repository will integrate the collective experience, the most suitable features, as well as all available data from existing repositories and projects, as outlined and summarized in Section 10.3. As rationalized in the same section above, an evolutionary design should be employed when building this new, open-access repository, aimed at fulfilling all the key features.

Such a unified repository should be all-inclusive with regard to the type of collected NMR data and avoid any bias towards certain approaches regarding the utility and/or future applications of the data. Importantly, the repository should support equally all methods for NMR-based structural dereplication such as 1D ^1^H, 1D ^13^C, 2D HSQC, and any hybrid approaches.

Notably, the foremost feature of the envisioned global repository is long term sustainability, as it represents the quintessential challenge research operations in general and databases in particular for environments that depend on extramural funding and lack independent revenue streams. The achievement of sustainability will greatly benefit from trans-institutional, trans-agency, trans-societal, international consortia and processes that actively involve (NMR) data-producing scientists.

#### 10.4.4 Global coordination

As with previous initiatives in other fields, the perhaps second most critically decisive factor for success in moving forward is to reach consensus in the global scientific community. Majority consensus is a requirement for broad acceptance, and reaching such a general agreement requires a balanced process that addresses and prioritizes all parameters and involves stakeholders broadly and equitably.

Predictably, the designation of actual sharing mechanisms and data formats are more likely to produce controversial discussion than the identification of wish-list features. Whether the establishment of the sharing mechanisms is driven by a (predictably) lengthier consensus process or a balanced group of representative experts, the utilization of existing resources is a lesser consideration than the modularity of the chosen approach and, foremost, that lack of any further delay.

The pre-determined data formats of current NMR instruments have evolved and are widely supported by third party software tools. While they likely will be replaced, or at least be used in parallel with, standardized and open formats, they still represent a good start for data sharing, and there is no reason to wait for the development of standards as data can be shared right now.

#### 10.4.5 Utility follows availability

As is typical for situations where a major change in the modus operandi needs to be implemented to achieve progress, certain levels of activation energy and patience are required before a stream of (major) benefits can follow. Accordingly, it shall be emphasized that, while the raw data (FID) archive proposed by this Raw Data Initiative is both a prerequisite and a major undertaking, it still represents a minimalist version of a greater NMR data collection for all kinds of purposes, including dereplication. A subsequent evolutionary development of methods is still needed to make (additional) use of the raw data, producing new insights and/or accelerating current processes. For example, while making a 1D ^1^H NMR FID publicly available today provides a highly conclusive means of dereplication, it still requires a tool that translates the non human-readable FID into a human readable spectrum. While more rapid and/or human intuitive methods can be developed in subsequent steps to achieve the same goal, they will most likely depend on the same and/or additional raw NMR data. Collectively, this re-emphasizes the recent call for making NMR information accessible for both humans and computers.^366^

### 10.5 Raw NMR and other data enhance the future of natural product research

#### 10.5.1 Raw data sharing as enabling technology

This review identified a multitude of rationales, why and how raw NMR data can provide useful and/or unprecedented insights. Presented cases exemplify the importance of sharing raw NMR data in NP research and fall into seven areas of broader impact: (i) the enhancement of the integrity of structure elucidation, which has major implications on downstream activities; (ii) the ability to document the purity status of a given material and enable future meta-analysis and/or refinement of the evaluation; notably, the (semi-)quantitative evaluations are often feasible even if the more rigorous conditions of quantitative NMR (qNMR)^29^,^389^–^391^ were not satisfied during data acquisition, and even if no internal calibration was used (feasibility of the 100% qNMR method);^29^,^390^ (iii) the enhancement of the accuracy and general capabilities of dereplication methods, thereby addressing one of the major challenges in metabolomics; (iv) enhancement of the amount of information obtained from NMR spectra and the speed of the mining process, by employing newly emerging software tools that depend on the availability of larger data sets; (v) the catalysis of developments in the field of the less studied NMR-accessible nuclei, offering new opportunities for the study of NPs and their analogues, of these fields; (vi) the promotion of the proven capabilities of existing repositories; (vii) the prospects of extending NP and metabolomic knowledge into clinical applications, *e.g.*, by using magnetic resonance spectroscopy (MRS). In all these instances, the raw NMR data can serve as the common denominator for progress.

#### 10.5.2 Learning from experience

In both analytical and NP chemistry, experience has shown that classical bulk analysis methods such as microanalytical and (mixed) melting point determinations are much more sensitive to minor impurities than many of the contemporary spectroscopic methods. Notably, despite a clear trend away from the classical methods employing chemical degradation, towards modern spectroscopy, the actual demand on the level of purity required for the meaningful bioactivity evaluation of NPs and other chemicals has not changed when considering the rigor and reproducibility of research outcomes. In this regard, raw NMR data can play important roles, *e.g.*, in the documentation of the constitution of a compound or material, and/or by potentially enabling the retrospective determination of the purity of previously investigated materials.

#### 10.5.3 Value of open science

As shown in this review, shared raw NMR data can generate new insight that otherwise is impossible to achieve. This value proposition can be transferred to other types of raw data, and has already been recognized for several, such as gene sequences, MS data (*via* GNPS) and X-ray diffraction data.

It is highly likely that the availability of a global repository of raw NMR data will potentiate productivity. Representing a tangential aspect of the call for raw NMR data sharing, recognition of the immense value of the information contained in raw (NMR) data triggers questions regarding intellectual property and data ownership. Notwithstanding the potential impact of the answers, which likely will vary by project, institution, and other factors, the body of evidence compiled in this review demonstrates that, at least from a scientific point-of-view, open sharing of raw data can generate an extraordinary amount of added scientific value. This benefit can apply to both, the sharing and the receiving scientists.

In the context of potential mutual benefit, the present findings provide support for the principles of Open Science, which seeks to enhance the accessibility of scientific research, data, and dissemination to the various levels of a society, including amateurs and professionals. While consideration of the benefit of access to shared resources *vs.* the desire of individual entities to profit is an open-ended discussion, the widely acknowledged complexity of research questions and endeavors, as well as global experience with multi-disciplinary research teams and approaches, indicate that availability and access to larger and more varied data sets bear major potential in advancing research outcomes.

## 11 Conflicts of interest

The authors declare the following competing financial interest(s): M. N. is founder of NMR Solutions Limited. Craig M. Williams is a consultant to EcoBiotics Ltd. The other authors declare no competing financial interest.

## 12 Note added after first publication

This article replaces the version published on 13th July 2018, which contained errors in the reference details of ref. 83–85.

## Supplementary Material

Supplementary information
