# Supplementary material for: The value of universally available raw NMR data for transparency, reproducibility, and integrity in natural product research
Source: Nat Prod Rep. 2018 Jul 13;36(1):35–107. doi: 10.1039/c7np00064b (PMC6350634; doi:10.1039/c7np00064b)
Supplement: Supplementary file 1 [file NP-036-C7NP00064B-s001.pdf]

Electronic Supplementary Material (ESI) for Natural Product Reports.
This journal is © The Royal Society of Chemistry 2018

The Value of Universally Available Raw NMR Data for Transparency, Reproducibility, and Integrity in Natural Product Research

James B. McAlpine,[a*] Shao-Nong Chen,[a] Andrei Kutateladze,[b] John B. MacMillan,[c] Giovanni Appendino,[d] Andersson Barison,[e] Mehdi A. Beniddir,[f] Maique W. Biavatti,[g] Stefan Bluml,[h] Asmaa Boufridi,[i] Mark S. Butler,[j] Robert J. Capon,[j] Young H. Choi,[k] David Coppage,[c] Phillip Crews,[c] Michael T. Crimmins,[l] Marie Csete,[m] Pradeep Dewapriya,[j] Joseph M. Egan,[n] Mary J. Garson,[o] Grégory Genta-Jouve,[p] William H. Gerwick,[q,r] Harald Gross,[s] Mary Kay Harper,[t] Precilia Hermanto,[u] James M. Hook,[u] Luke Hunter,[u] Damien Jeannerat,[v] Nai-Yun Ji,[w] Tyler A. Johnson,[c] David G. I. Kingston,[x] Hiroyuki Koshino,[y] Hsiau-Wei Lee,[c] Guy Lewin,[f] Jie Li,[r] Roger G. Linington,[n] Miaomiao Liu,[i] Kerry L. McPhail,[z] Tadeusz F. Molinski,[aa] Bradley S. Moore,[q,r] Joo-Won Nam,[ab] Ram P. Neupane,[ac] Matthias Niemitz,[ad] Jean-Marc Nuzillard,[ae] Nicholas H. Oberlies,[af] Fernanda M. M. Ocampos,[e] Guohui Pan,[ag] Ronald J. Quinn,[i] D. Sai Reddy,[b] Jean-Hugues Renault,[ae] José Rivera-Chávez,[ah] Wolfgang Robien,[ai] Carla M. Saunders,[aj] Thomas J. Schmidt,[ak] Christoph Seger,[al] Ben Shen,[ag] Christoph Steinbeck,[am] Hermann Stuppner,[al] Sonja Sturm,[al] Orazio Taglialatela-Scafati,[an] Dean J. Tantillo,[aj] Robert Verpoorte,[k] Bin-Gui Wang,[ar] Craig M. Williams,[o] Philip G. Williams,[ac] Julien Wist,[ao] Jian-Min Yue,[ap] Chen Zhang,[aq] Zhengren Xu,[ag] Charlotte Simmler,[a] David C. Lankin,[a] Jonathan Bisson,[a] Guido F. Pauli[a*]

Supporting Information

Table of Contents

part	title	page
SI1	Spectroscopic Data of the Fluorinated Amino Acids	SI-2

SI1. Spectroscopic Data of the Fluorinated Amino Acids

^{1}H (600 MHz, $CDCl_3$): Major isomer (~55%): 4.589 (dd, *J* = 9.2 and 5.2 Hz); 3.844 (dd, *J* = 13.1 and 12.2 Hz); 3.778 (dd, *J* = 13.1 and 12.2 Hz); 2.716 (ddd, *J* = 14.3, 13.1 and 9.2 Hz); 2.659 (ddd, *J* = 14.3, 13.1 and 5.2 Hz); 1.500 (s); Minor isomer (~ 45%): 4.494 (dd, *J* = 9.2 and 5.2 Hz); 3.896 (dd, *J* = 13.1 and 12.2 Hz); 3.829 (dd, *J* = 13.1 and 12.2 Hz); 2.778 (ddd, *J* = 13.7, 13.1 and 9.2 Hz); 2.659 (ddd or dq, *J* = 13.7, 13.1 and 5.2 Hz); 1.450 (s)

^{1}H-decoupled ^{19}F (600 MHz [^{1}H], $CDCl_3$): Major isomer: -99.470 (s, $w_{1/2}$ ~ 17.7 Hz); minor isomer: -99.632 (s, $w_{1/2}$ ~ 9.5 Hz)

^{19}F (600 MHz [^{1}H], $CDCl_3$): Major isomer: -99.470 (p, $^{3}J_{H\text{-}F}$ ~ 13.1 Hz); minor isomer: -99.632 (p, $^{3}J_{H\text{-}F}$ ~ 13.1 Hz)
